# CCL4 contributes to aging related angiogenic insufficiency through activating oxidative stress and endothelial inflammation

**DOI:** 10.1007/s10456-024-09922-y

**Published:** 2024-05-13

**Authors:** Ting-Ting Chang, Liang-Yu Lin, Ching Chen, Jaw-Wen Chen

**Affiliations:** 1 Department and Institute of Pharmacology, National Yang Ming Chiao Tung University, Taipei, Taiwan; 2 School of Medicine, National Yang Ming Chiao Tung University, Taipei, Taiwan; 3 Biomedical Industry Ph.D. Program, National Yang Ming Chiao Tung University, Taipei, Taiwan; 4 Cardiovascular Research Center, Taipei Medical University Hospital and Taipei Medical University, Taipei, Taiwan; 5 Division of Endocrinology and Metabolism, Department of Medicine, Taipei Veterans General Hospital, Taipei, Taiwan; 6 Division of Cardiology, Department of Medicine, Department of Research, Taipei Medical University Hospital, Taipei, Taiwan; 7 Division of Cardiology, Department of Medicine, Taipei Veterans General Hospital, Taipei, Taiwan; 8 Cardiovascular Research Center, National Yang Ming Chiao Tung University, Taipei, Taiwan; 9 Department and Institute of Pharmacology, School of Medicine, National Yang Ming Chiao Tung University, Taipei, Taiwan; 10 Department of Research, Taipei Medical University Hospital, Taipei, Taiwan

**Keywords:** Aging, CCL4, Inflammation, Oxidative stress, Vascular dysfunction

## Abstract

Aging is a natural process associated with chronic inflammation in the development of vascular dysfunction. We hypothesized that chemokine C-C motif ligands 4 (CCL4) might play a vital role in aging-related vascular dysfunction. Circulating CCL4 was up-regulated in elderly subjects and in aged animals. CCL4 inhibition reduced generation of reactive oxygen species (ROS), attenuated inflammation, and restored cell functions in endothelial progenitor cells from elderly subjects and in aged human aortic endothelial cells. CCL4 promoted cell aging, with impaired cell functioning, by activating ROS production and inflammation. CCL4 knockout mice and therapeutic administration of anti-CCL4 neutralizing antibodies exhibited vascular and dermal anti-aging effects, with improved wound healing, via the down-regulation of inflammatory proteins and the activation of angiogenic proteins. Altogether, our findings suggested that CCL4 may contribute to aging-related vascular dysfunction via activating oxidative stress and endothelial inflammation. CCL4 may be a potential therapeutic target for vascular protections during aging.

**Supplementary Information:**

The online version contains supplementary material available at 10.1007/s10456-024-09922-y.

## Introduction

Aging is a sophisticated natural process accompanied by molecular and cellular decline. During the aging process, excessive reactive oxygen species (ROS) generation and activated inflammation cause further cell damage [1]. In a clinical setting, aging leads to the dysregulation of homeostasis and to susceptibility to chronic inflammatory diseases [2]. The aggravation of chronic inflammation may eventually lead to age-related cardiovascular diseases [3].

Chemokines play a key role in the progression of cardiovascular diseases [4]. Chemokine CC motif ligand 4 (CCL4) is an inflammatory chemokine belonging to the CC chemokine family [5]. Patients with higher CCL4 levels were observed to have a higher risk of stroke and cardiovascular events [6]. Higher CCL4 levels were also observed in patients with type 2 DM and/or atherosclerotic cardiovascular diseases [7–9, 6, 10]. CCL4 could induce ROS production and the adhesion of THP-1 cells to human umbilical vein endothelial cells [6]. The outcomes of the above studies suggest the participation of CCL4 in inflammation-related diseases. Moreover, our recent study indicated that CCL4 inhibition could stabilize plaques and decrease endothelial cell adhesiveness in atherosclerosis [11]. In addition, direct inhibition of CCL4 may improve endothelial cell function and ischemia-induced angiogenesis in various diabetic animals [12]. Interestingly, CCL4 expression has been shown to increase with age in human immune cells [13]. However, it is not known if CCL4 contributes to aging-related vascular damage, especially in vascular endothelial cells.

Given the potential impacts of CCL4 upon various cardiovascular diseases, we hypothesized that CCL4 might play a mechanistic role in aging-related vascular dysfunction. In this paper, we report that circulating CCL4 was up-regulated in elderly subjects. In addition, in vitro CCL4 blockade improved cell function, reduced ROS generation, and reversed senescence, inflammation and angiogenesis signal pathways in primary-cultured endothelial progenitor cells (EPCs) from elderly subjects, and also in aged vascular endothelial cells. Administration of CCL4 directly induced cell senescence and dysfunction accompanied by enhanced ROS generation in human aortic endothelial cells (HAECs). Furthermore, CCL4 was up-regulated in aged animals. Compared with aged wild-type mice, aged CCL4 knockout mice showed improved neovascularization and wound healing, with reduced aging and inflammatory markers, as well as increased expression of angiogenic proteins in aortic and wound tissues. These findings indicate the comprehensive role of CCL4 in aging-related vascular dysfunction and provide a rationale for a potential new anti-aging strategy.

## Materials and methods

### Clinical study

#### Clinical samples

The blood samples were collected from the peripheral veins of healthy volunteers aged between 20 and 30 years (young group) and between 55 and 70 years (elderly group). There are at least 6 subjects in each group. Subjects with diabetes mellitus, significant systemic diseases, who had received major operations in the past 6 months were excluded. Demographic and clinical data were obtained at enrollment. The human study was approved by the institute research committee (IRB No. 2021-02-010AC in Taipei Veterans General Hospital) and was conducted in accordance with the guidelines detailed in the Declaration of Helsinki.

### In vitro study

#### Cell culture

After blood was collected, the total mononuclear cells were separated by using a Histopaque-1077 (Sigma-Aldrich, 10,771, Darmstadt, Germany) and centrifuged at 500 × g at room temperature for 30 min. The mononuclear cells were cultured in an endothelial cell basal medium (EBM-2; Lonza, CC-3156, Basel, Switzerland) with supplements, including hydrocortisone, human fibroblast growth factors, vascular endothelial growth factor (VEGF), R3-insulin-like growth factor-1, ascorbic acid, human epidermal growth factor, gentamicin sulfate-amphotericin, and 20% fetal bovine serum on fibronectin-coated 6-well plates. After 2–4 weeks culture, attached EPCs emerged. The EPCs were in the shape of cobblestones; this kind of shape is the typical monolayer growth pattern of mature endothelial cells.

Primary HAECs (ScienCell, Catalog #6100, Carlsbad, CA, USA) were cultured in fibronectin-coated plates with endothelium cell medium containing 5% fetal bovine serum and 1% of endothelial cell growth supplement in an atmosphere of 95% air and 5% CO_2_ at 37 °C. Different dosages of CCL4 (0.1, 1 ng/mL; R&D Systems, Minnesota, USA) were added to the cells as the cells grown to a certain circumstance. The cells were then cultured at 37 °C and 5% CO_2_ for 3 days after CCL4 stimulation. Edaravone (MCI186) were purchased from Cayman Chemical (Ann Arbor, MI, USA) and was used as a free radical scavenger.

#### Transfection of siRNA

Cells were transfected with ccl4 siRNA and p65 siRNA (Santa Cruz Biotechnology, sc-43,932 and sc29410, Dallas, TX, USA) using Lipofectamine 2000 (Invitrogen, Carlsbad, CA, USA) in culture medium.

#### Cell proliferation assay

Cell Counting Kit-8 (CCK-8; Dojindo Molecular Technologies, Inc., Rockville, MD, USA) was used to evaluate cell viability according to the manufacturer’s instructions. In brief, reagents were added and incubated with cells at 37 °C for 2 h and the absorbance at 450 nm was determined.

#### β-Galactosidase staining

The senescence phenotype was detected using the β-galactosidase staining kit (Merck, Darmstadt, Germany). The number of positive senescence-associated β-galactosidase cells were observed by light microscopy in 10 randomly chosen low-power fields.

#### ROS generation assay

Hydrogen peroxide production from cells was measured using the Amplex Red Hydrogen Peroxide/Peroxidase Assay Kit (Invitrogen, Carlsbad, CA, USA). Briefly, horseradish peroxidase catalyzed the stoichiometric reaction of Amplex Red with hydrogen peroxide (H_2_O_2_), composing of water and resorufin. Then, cells were scraped into phosphate buffer (pH 7.4). After spin-down of cell debris, 50 µL of supernatant was loaded with 50 µL of working solution. Following a 30-minute incubation, fluorescence was measured using a microplate reader with excitation at 540 nm and emission at 590 nm.

#### Migration and tube formation assay

The migration was evaluated by a chamber assay. Cells (1 × 10^4^ cells) were resuspended in the culture medium with 5% FBS. The cells were added to the upper chamber of a 24-well transwell plate with a polycarbonate membrane. Culture medium supplemented with fetal bovine serum was added to the lower chamber, and the chambers were incubated for 18 h. After incubation, the membrane was fixed with 4% paraformaldehyde and stained using hematoxylin solution. The numbers of migrated cells were counted in random high-power (×100) microscopic fields.

The in vitro tube formation assay was performed with an angiogenesis assay kit (Invitrogen, Carlsbad, CA, USA). ECMatrix gel solution was mixed with ECMatrix diluent buffer and placed in a 96-well plate. Then, cells (1 × 10^4^ cells) were placed on a matrix solution with 10% FBS culture medium and incubated for 16 h. Tube formation was inspected under an inverted light microscope (× 40). The average of the total area of complete tubes formed by cells was compared using Image-Pro Plus (Media Cybernetics, Inc. Rockville, MD, USA).

#### Western blot analysis

Total cell or tissue lysates were extracted using a lysis buffer, and proteins were separated in 8–12% (v/v) SDS-PAGE gels. After electrophoresis (Bio-Rad Laboratories, Hercules, CA, USA), the proteins were transferred onto nitrocellulose membranes (Millipore, Darmstadt, Germany). The membranes were incubated with antibodies against CCL4 (Santa Cruz Biotechnology, sc-393,441, Dallas, TX, USA); p-AKT (BD Biosciences, 550,747; NJ, USA); AKT (BD Biosciences, 610,868; NJ, USA); VEGF (Santa Cruz Biotechnology, sc-152; Dallas, TX, USA); SDF-1 (Cell Signaling, 3530 S; Boston, MA, USA); IL-1β (Santa Cruz Biotechnology, sc-7884; Dallas, TX, USA); IL-6 (Cell Signaling, 12,153 S; Boston, MA, USA); TNF-α (Cell Signaling, 3707 S; Boston, MA, USA); p-p65 (Cell Signaling, 3031 S; Boston, MA, USA); p65 (BD Biosciences, 610,868; NJ, USA); SIRT1 (Cell Signaling, 8469 S; Boston, MA, USA); p53 (Cell Signaling, 2524 S; Boston, MA, USA); p16 (Cell Signaling, 80,772 S and 29,271 S ; Boston, MA, USA); xanthine oxidase (Santa Cruz, sc-398,548, Dallas, TX, USA); NADPH oxidase subunit p47 (Santa Cruz, sc-17,845, Dallas, TX, USA); p-eNOS (Cell Signaling, 9571 S; Boston, MA, USA); eNOS (Cell Signaling, 32,027 S; Boston, MA, USA) and β-actin (Merck, MAB1501, Darmstadt, Germany) [14] at 4 °C overnight. The above protein expressions were normalized to that of actin expression.

### In vivo study

#### Animal preparation

Six-week-old male C57BL/6JNarl-Ccl4em1 knock out (CCL4KO) mice were design and purchased from the National Laboratory Animal Center (Taipei, Taiwan). CCL4KO mice were generated in a C57BL/6JNarl genetic background by using the CRISPR/Cas9 system. All mice were genotyped using PCR with specific primers (forward, 5′-TCTCCCTCCTTTCTCTTCCGTG-3′, and reverse, 5′-TCTACTCCCAATGATGGCTGACC-3′). C57BL/6JNarl mice were used as the wild-type (WT) control. Six-month-old mice were defined as the young group and eighteen-month-old mice were defined as the elderly group. Some animals were randomized into the CCL4 neutralizing antibody-injected group (100 µg, intraperitoneal injection; R&D Systems, MAB451, Minneapolis, MN, USA). The monoclonal neutralizing antibody was injected 3 times a week for 2 weeks. The animals were raised under specific pathogen-free conditions and all mice were kept in microisolator cages on a 12-hour day/night cycle in the animal center of National Yang Ming Chiao Tung University (Taipei, Taiwan) according to the regulations of the Animal Care Committee of National Yang Ming Chiao Tung University. The animal study was approved by the Animal Care Committee of National Yang Ming Chiao Tung University (IACUC No. 1101114).

#### Wound healing assay and evaluations of morphological changes in the wound area

The circular full-thickness excisional wounds of 3 mm of diameter were generated with biopsy punch without injuring the muscle. The wounds were recorded using a digital camera (Nikon, Tokyo, Japan) after they were generated. Transverse slices of the wound areas were fixed in 10% formaldehyde, embedded in paraffin, and mounted onto slides for staining. Hematoxylin/Eosin (H&E) stains were used to evaluate the morphological changes in the wound area.

#### Capillary densities, cell proliferation, and collagen deposition levels in the wound area

Sections were de-paraffinized and incubated with a rat-monoclonal antibody against murine CD31 (Abcam, 124432, Waltham, MA, USA) and a rabbit-polyclonal antibody against the murine marker of proliferation Ki67 (Novus, NB500-170, Minneapolis, MN, USA). Antibody distribution was visualized with the avidin-biotin-complex technique and Vector Red chromogenic substrate, followed by counterstaining with hematoxylin. Sections were allowed to dry overnight and stained with H&E and Masson’s trichrome-stained for histological analysis.

#### Matrigel plug neovascularization assay

Mice were injected subcutaneously with growth factor reduced basement membrane matrix (Corning® Matrigel, Glendale, AZ, USA) containing 30 ng/mL VEGF (Peprotech, Rocky Hill, CT, USA) and 50 U heparin (Sigma-Aldrich, Darmstadt, Germany). Plugs were collected after 14 days and homogenized in 500 µL of cell lysis buffer and centrifuged at 6000 g at 4 °C for 60 min. Hemoglobin was detected at 400 nm wavelength by using a colorimetric assay (Sigma-Aldrich, MAK115, Darmstadt, Germany). Also, plugs were harvested for histological and immunohistochemistry analysis.

#### Aortic ring assay

The detailed method and timing of tissue culture have been well indicated in our recent publication [15]. In brief, the aortic rings were cut a 0.5 mm and embedded 1 mg/mL type 1 rat tail collagen matrix (Millipore, Darmstadt, Germany) and incubated for 1 h at 37 °C. Aortic rings were cultured in EBM-2 (Lonza, Basel, Switzerland) containing 2.5% bovine serum (Gibco, Carlsbad, CA, USA), 50 U/mL penicillin and 0.5 mg/mL streptomycin (Sigma-Aldrich, Darmstadt, Germany) and 30 ng/mL VEGF (Peprotech, Rocky Hill, CT, USA) in 24-wells for 7 days. Images were captured using a microscope (× 100).

#### ELISA

The concentrations of CCL4 were analyzed using a CCL4 ELISA kits (R&D, Minneapolis, MN, USA) according to the manufacturer’s instructions.

### Statistical analysis

Given our data’s non-parametric nature, we utilized the median and interquartile for descriptive statistics and the Mann-Whitney U test to identify group differences. The statistical analysis software GraphPad Prism vision 6 was used for the statistical analysis. A p-value of less than 0.05 was deemed statistically significant.

## Results

### Clinical study

#### Increased plasma CCL4 concentrations in elderly subjects

**Fig. 1:**
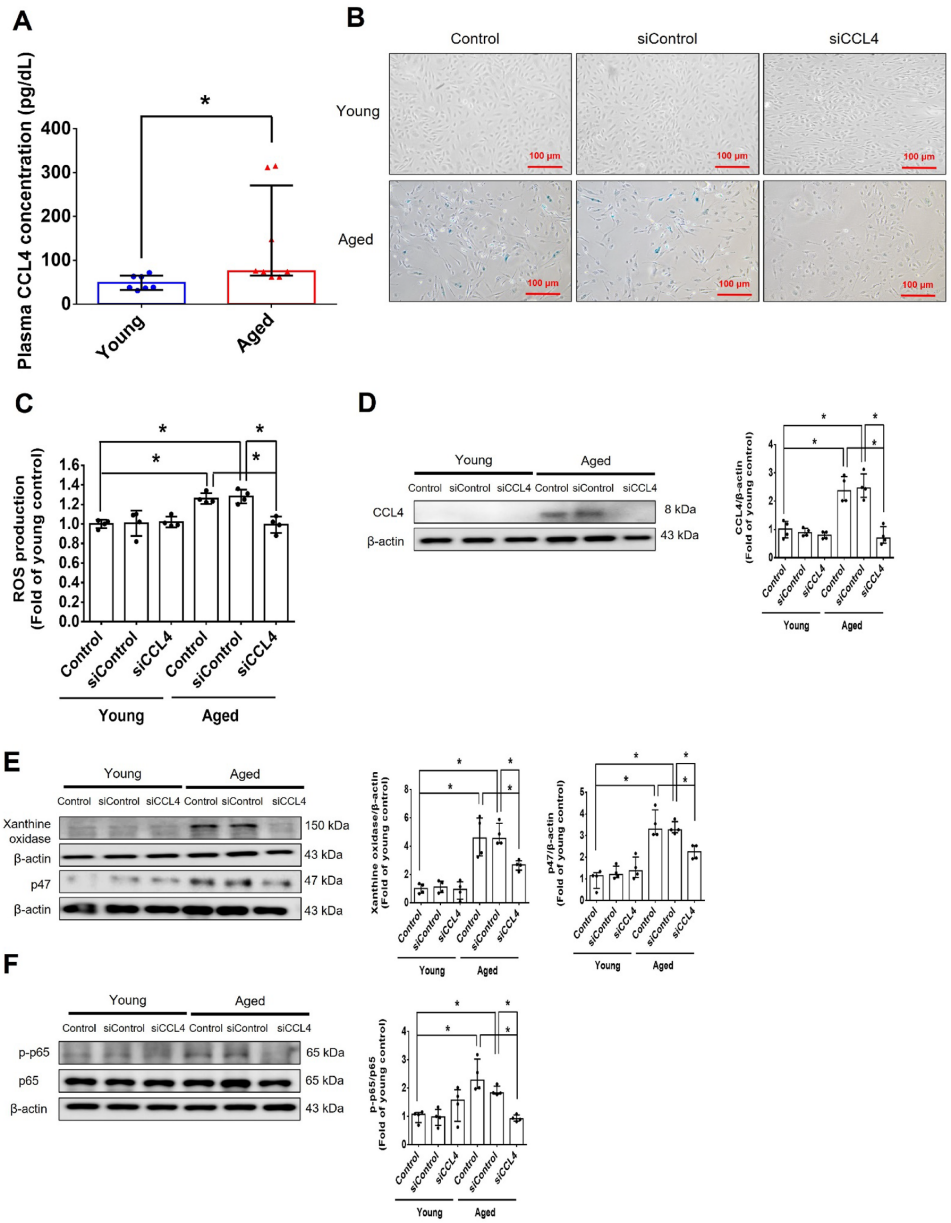

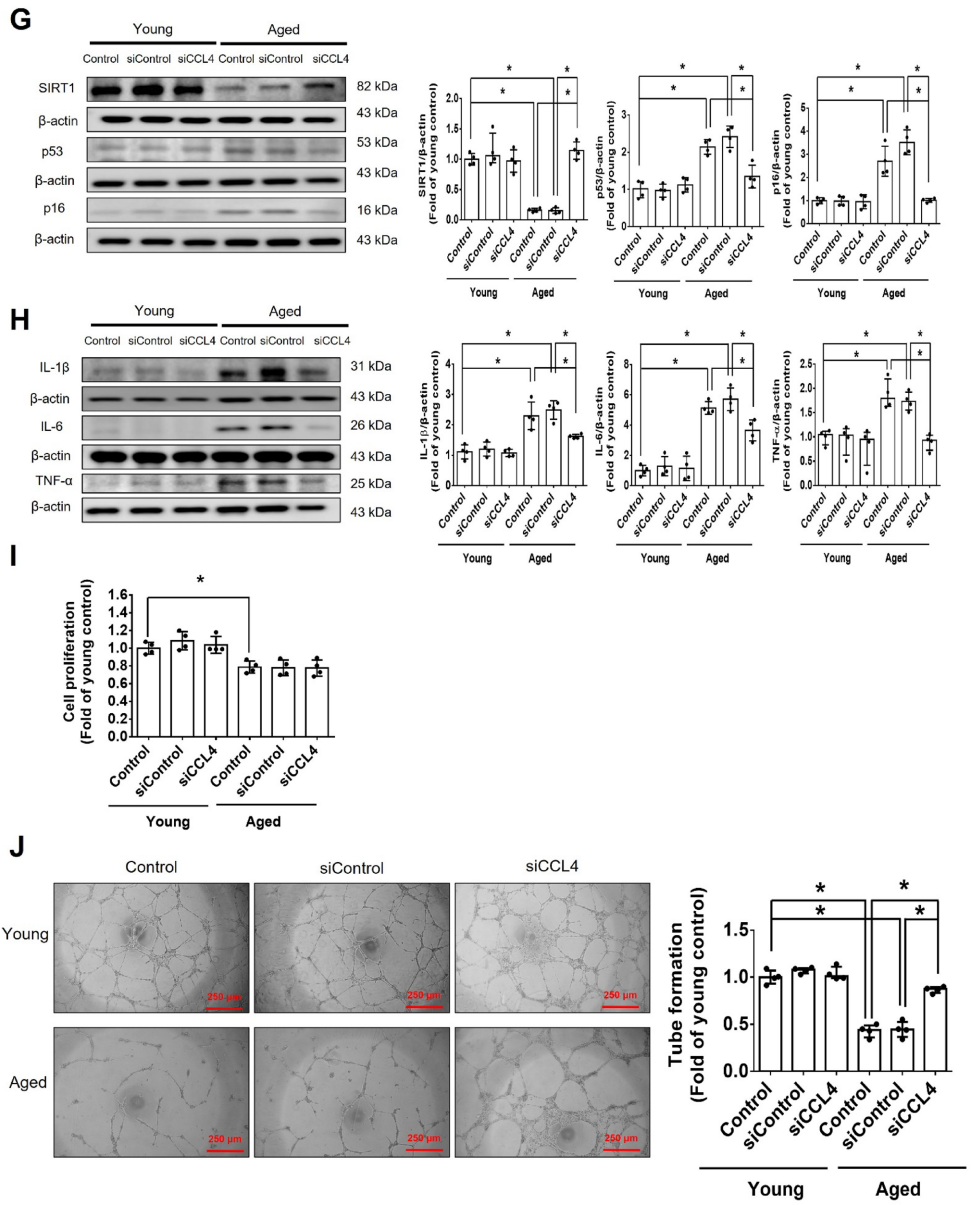

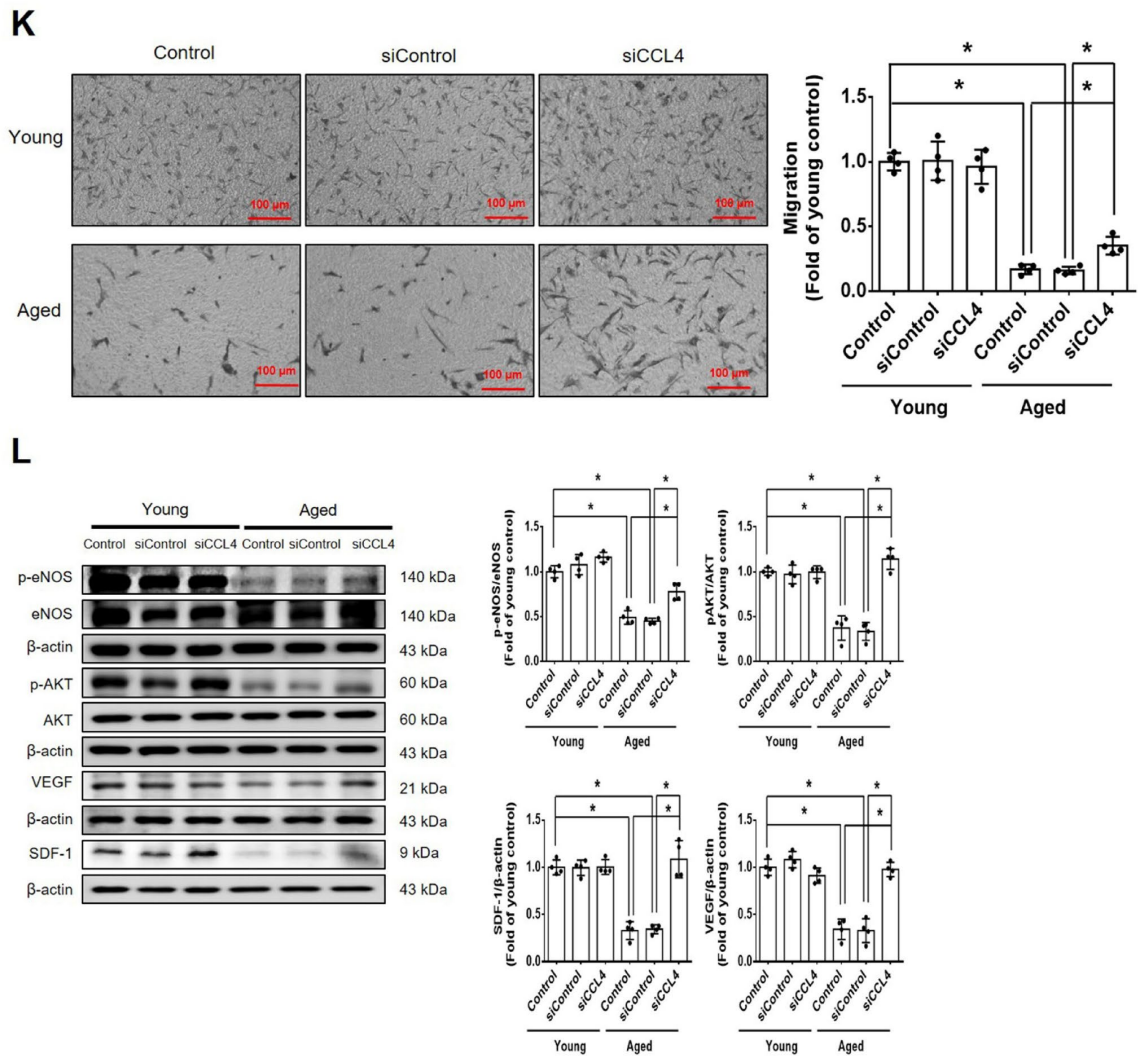
Inhibition of CCL4 reversed cell aging and inflammation in EPCs from aged subjects. **A** Elderly subjects (> 55 years old; *n* = 8) had higher plasma CCL4 concentrations compared to the young subjects (< 30 years old; *n* = 7). **B** Inhibition of CCL4 reduced senescence of EPCs from aged subjects. **C** Inhibition of CCL4 reduced ROS productions in EPCs from aged subjects (*n* = 4). **D** and **F** Western blotting and statistical analysis of CCL4, xanthine oxidase, p47, and p-p65 in primary cultured EPCs (*n* = 4). **G** and **H** Western blotting and statistical analysis of SIRT1, p53, p16, IL-1β, IL-6, and TNF-α expression in primary cultured EPCs (*n* = 4). **I** Inhibition of CCL4 did not affect cell proliferation in HAECs (*n* = 4). **J** and **K** Inhibition of CCL4 improved tube formation and migration abilities in EPCs from aged subjects (*n* = 4). **L** Western blotting and statistical analysis of eNOS, p-AKT, VEGF, and SDF-1 expression in primary cultured EPCs (*n* = 4). *P < 0.05, **P < 0.01

Clinical characteristics of the study population was analyzed **(**Table 1**)**. Plasma was collected from young (< 30 years old) and aged (> 55 years old) subjects. Plasma CCL4 levels were increased in the aged subjects, as compared to the young individuals **(**Fig. 1A**)**, suggesting the potential involvement of CCL4 in the systemic as well as vascular aging process.

**Table 1:** Clinical characteristics of the study population

	Young subjects (*n* = 7)		Aged subjects (*n* = 8)	
	Median	Q1-Q3	Median	Q1–Q3
Male gender (%)	47%		61%	
Age (years)	27	25–29	66**	57–75
Fasting glucose (mg/dL)	90	82–98	100	92–108
Cholesterol (mg/dL)	177	147–207	180	157–203
Triglycerides (mg/dL)	92	78–147	115	60–170
Creatinine (mg/dL)	0.75	0.56–0.94	0.84	0.68–1.0
ALT (U/L)	20.4	12.5–28.3	24.7	10.7–38
AST (U/L)	21.5	6.8–36.2	25.8	18.0–33.6
Oral glucose-lowering drugs	–		–	
Blood pressure drugs	–		33%	
Lipid-lowering drugs	–		28%	

Data are presented as median with interquartile range

The Mann-Whitney U test was used to determine statistically significant differences

*ALT* Alanine aminotransferase *AST* Aspartate aminotransferase.

Data were considered statistically significant when the P-value was < 0.05

**P < 0.01 compared with young subjects

### In vitro study

#### CCL4 inhibition improved cell dysfunction in EPCs from aged subjects

Cell senescence was markedly increased in EPCs from aged (> 55 years old) subjects, compared with EPCs from young (< 30 years old) subjects. The inhibition of CCL4 by siRNA reduced senescence in EPCs cultured from aged subjects **(**Fig. 1B and Fig. S1A**)**. ROS generation was increased in EPCs cultured from aged subjects but was decreased in the siCCL4-treated group (Fig. 1C). The expression of CCL4 was up-regulated in EPCs cultured from aged subjects but was decreased by administration of siCCL4 (Fig. 1D). CCL4 inhibition reduced the ROS production proteins, such as xanthine oxidase and p47 in EPCs cultured from aged subjects (Fig. 1E). Expression of phosphorylated p65 was increased in EPCs cultured from aged subjects but was decreased by administration of siCCL4 (Fig. 1F). Inhibition of CCL4 also reversed aging markers such as SIRT1, p53, and p16 (Fig. 1G), as well as inflammatory proteins such as interleukin (IL)-1β, IL-6, and tumor necrosis factor (TNF)-α (Fig. 1H).

CCL4 inhibition by siRNA did not facilitate cell proliferation in EPCs cultured from aged subjects (Fig. 1I). In functional assays, tube formation and migration abilities were attenuated in EPCs from aged subjects and were notably improved by siCCL4 (Fig. 1J and K). The angiogenic factors, including p-endothelial nitric oxide synthase (eNOS), p-AKT, vascular endothelial growth factor (VEGF), and stromal cell-derived factor (SDF)-1, were reduced in EPCs from aged subjects but were up-regulated in the CCL4-inhibited group (Fig. 1L). Altogether, the above observations indicate that the inhibition of CCL4 could reduce inflammation, restore age-impaired cell functions, and increase the levels of angiogenic proteins in EPCs in aged subjects.

#### CCL4 directly caused cell aging and enhanced ROS generation in HAECs

**Fig. 2:**
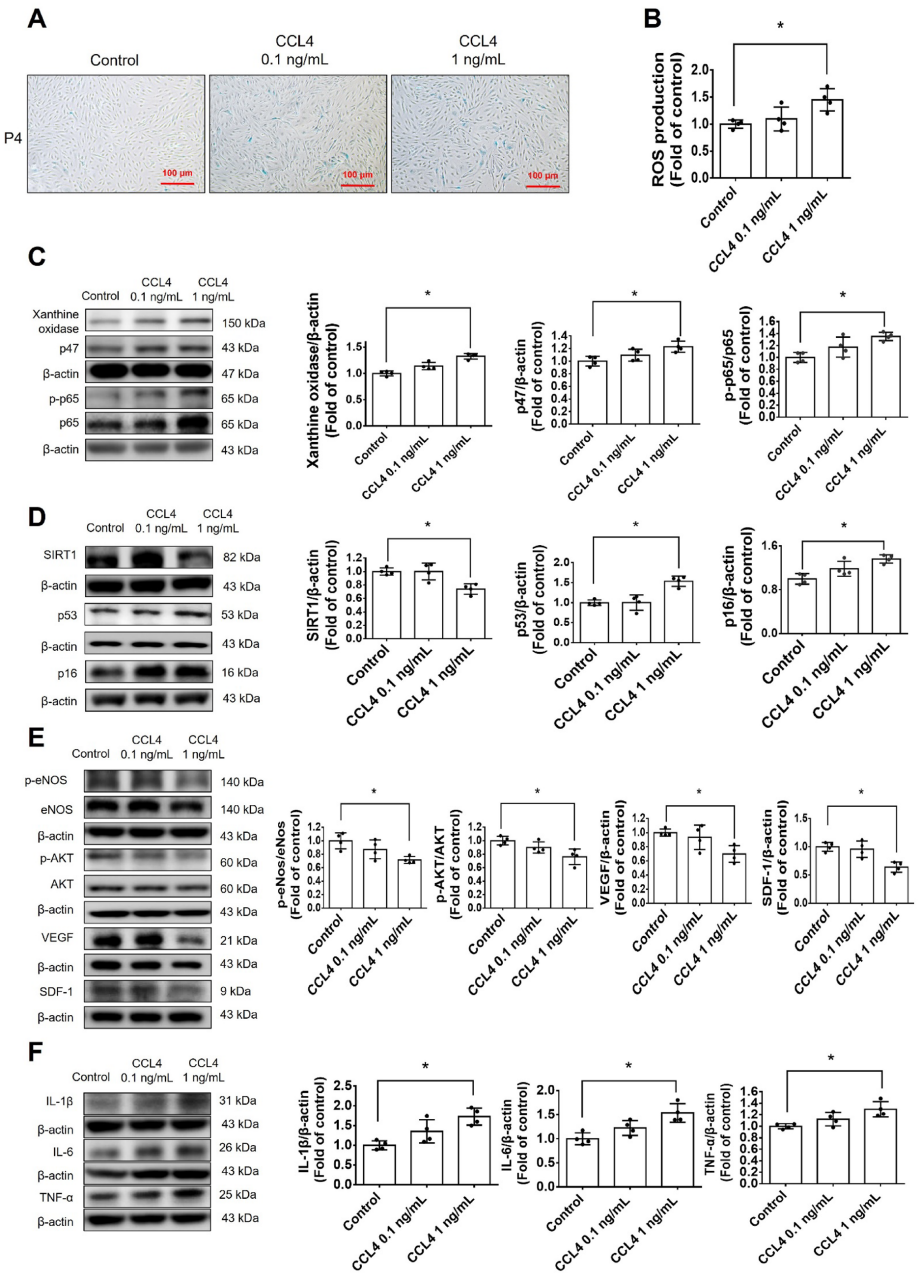
Administration of CCL4 caused cell aging and ROS generation in HAECs. **A** and **B** Cell senescence and ROS productions were increased in CCL4-treated HAECs (*n* = 4). **C** Western blotting and statistical analyses of ROS production proteins, such as xanthine oxidase and p47 (*n* = 4). **D** Western blotting and statistical analyses of aging factors, such as SIRT1, p53, and p16 (*n* = 4). **E** Western blotting and statistical analyses of angiogenic factors, such as eNOS, p-AKT, VEGF, and SDF-1 (*n* = 4). **F** Western blotting and statistical analyses of inflammatory factors, such as IL-1β, IL-6, and TNF-α (*n* = 4). *P < 0.05, **P < 0.01

Cell senescence and ROS generation were significantly increased in young cells (passage 4; P4) treated with CCL4 (Fig. 2A, Fig. S1B, and Fig. 2B). CCL4 directly upregulated the expressions of xanthine oxidase and p47 in HAECs (Fig. 2C). The administration of CCL4 induced aging markers, such as SIRT1, p53, and p16 (Fig. 2D); reduced the levels of angiogenic proteins, such as p-eNOS, p-AKT, VEGF, and SDF-1 (Fig. 2E); and increased the levels of inflammatory proteins, such as IL-1β, IL-6, and TNF-α (Fig. 2F). In brief, these findings confirmed the direct effects of CCL4 on endothelial cell aging, and related inflammatory results.

#### CCL4 inhibition reversed cell aging and reduced inflammation in HAECs

**Fig. 3:**
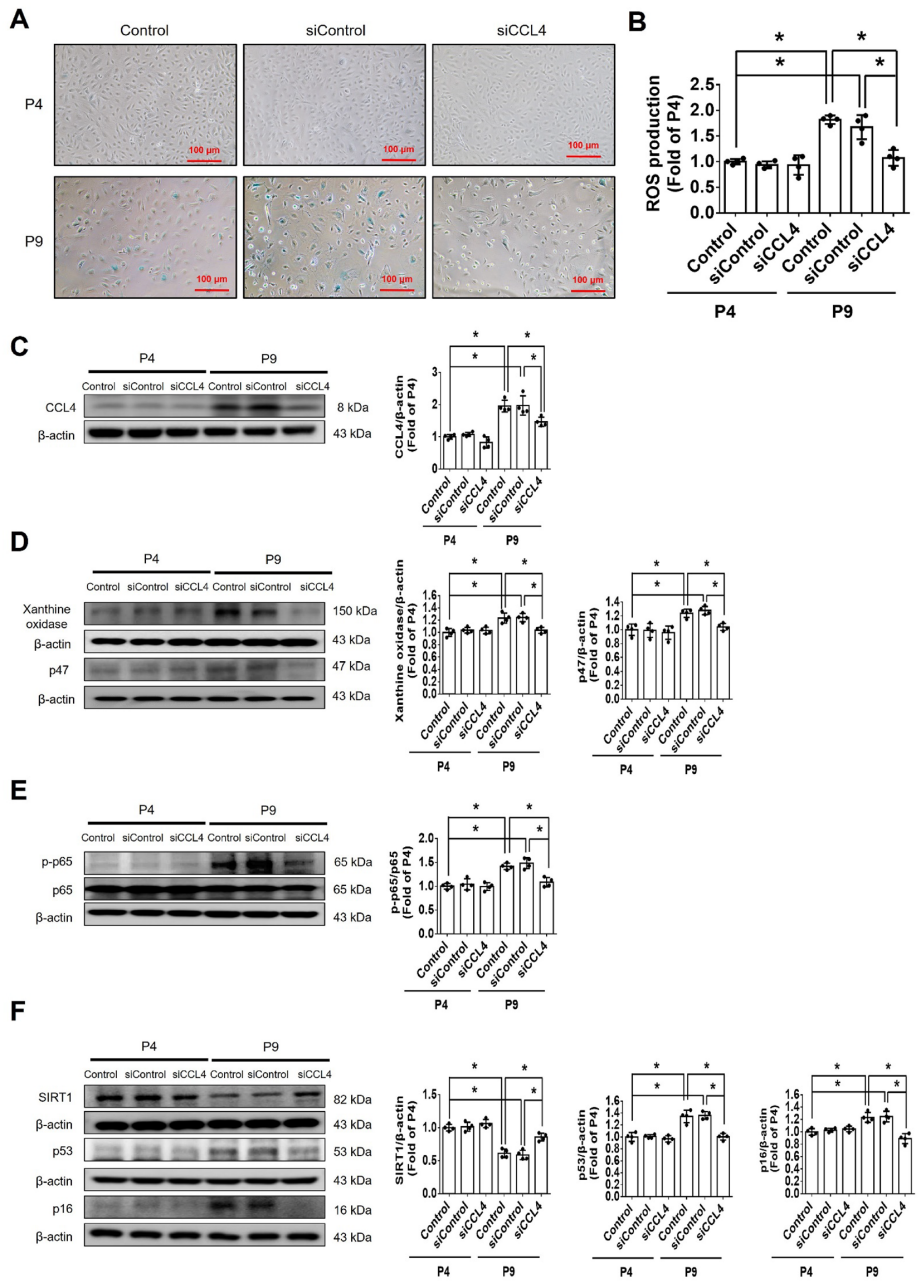

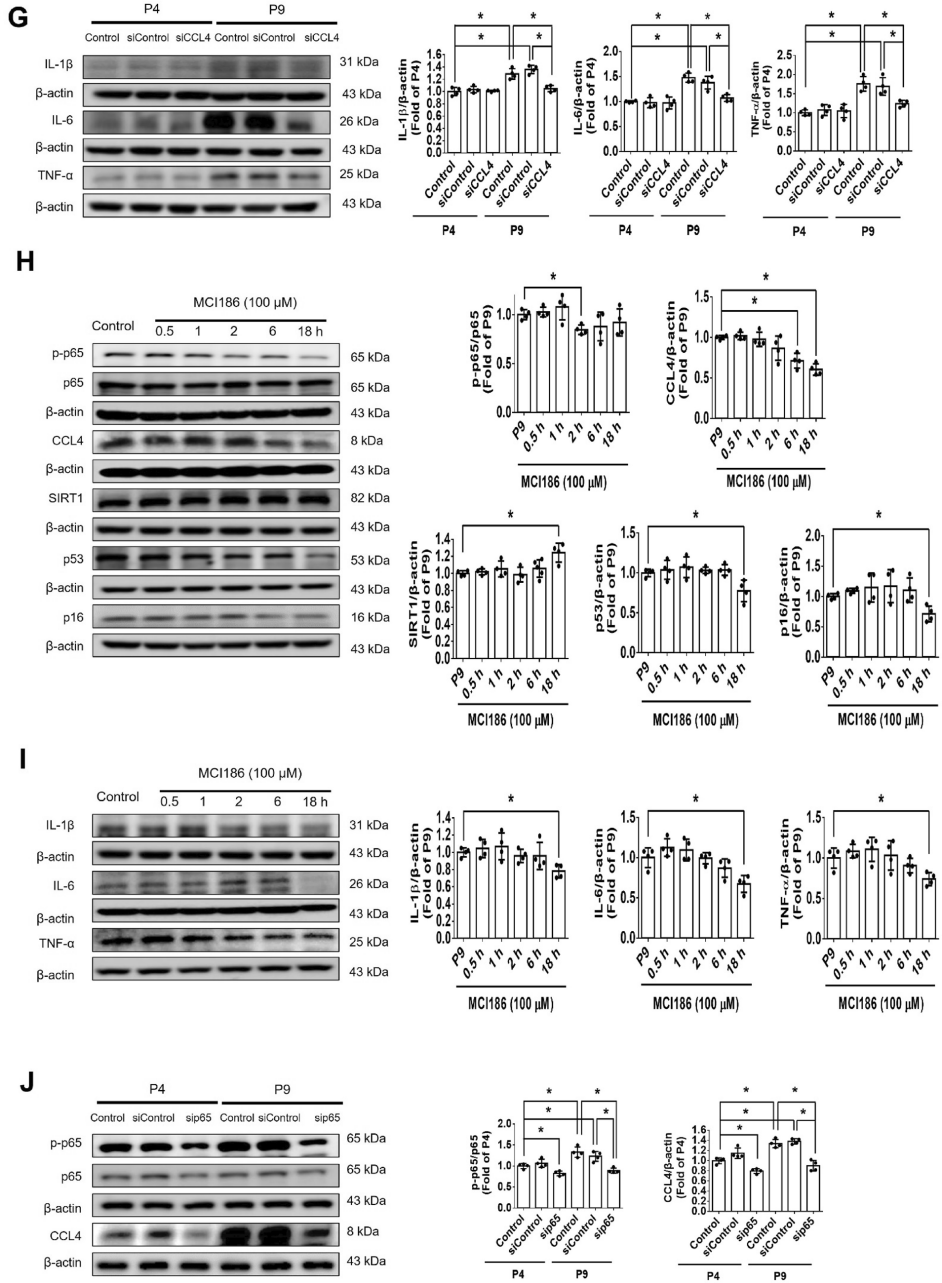
Inhibition of CCL4 reversed cell aging and inflammation in HAECs. **A** Inhibition of CCL4 reduced senescence of aged HAECs (*n* = 4). **B** Inhibition of CCL4 reduced ROS productions in aged HAECs (*n* = 4). **C** Western blotting and statistical analysis of CCL4 in HAECs (*n* = 4). **D** Western blotting and statistical analysis of xanthine oxidase and p47 in HAECs (*n* = 4). **E** Western blotting and statistical analysis of p-p65 and p65 in HAECs (*n* = 4). **F**, **G** Western blotting and statistical analysis of SIRT1, p53, p16, TNF-α, IL-1β and IL-6 expression in HAECs (*n* = 4). **H**, **I** Western blotting and statistical analysis of p-p65, CCL4, SIRT1, p53, p16, IL-1β, IL-6, and TNF-α expression after administration of MCI186 in aged HAECs (*n* = 4). **J** Western blotting and statistical analysis of p-p65 and CCL4 after administration of p65 siRNA in aged HAECs (*n* = 4). P4, passage 4; P9, passage 9; siCCL4, siRNA of CCL4; MCI186, a free radical scavenger. *P < 0.05, **P < 0.01

In vitro experiments were also conducted with human aortic endothelial cells (HAECs) to elucidate the mechanistic role of CCL4 in aging-related vascular dysfunction. Cell senescence was significantly increased in aged HAECs (passage 9; P9) compared to young HAECs (passage 4; P4). The administration of CCL4 siRNA reduced the senescence of HAECs (Fig. 3A and Fig. S1C). ROS generation was enhanced in aged HAECs and was reduced in the siCCL4-treated group (Fig. 3B). The expression of CCL4 was higher in aged HAECs and was decreased by administration of siCCL4 (Fig. 3C). CCL4 inhibition reduced the ROS production proteins, such as xanthine oxidase and p47 in aged HAECs (Fig. 3D). The expression of phosphorylated p65 was higher in aged HAECs and was decreased by administration of siCCL4 (Fig. 3E). Inhibition of CCL4 reversed the aging markers, such as SIRT1, p53, and p16 (Fig. 3F). Moreover, CCL4 inhibition reduced the aging-induced inflammatory proteins, such as IL-1β, IL-6, and TNF-α (Fig. 3G).

To further address the relationship between CCL4 and ROS generation, as well as the downstream signaling pathway of CCL4-induced cell damage, MCI186 was used as a free radical scavenger in the following experiments. Phosphorylated p65 was observed to be down-regulated after the administration of MCI186 for 2 h. The expression of CCL4 was down-regulated after the administration of MCI186 for 6 h. Then, the downstream aging markers, including SIRT1, p53, and p16, were reversed after the administration of MCI186 for 18 h (Fig. 3H). On the other hand, the levels of downstream inflammatory proteins, including IL-1β, IL-6, and TNF-α, were reduced after the administration of MCI186 for 18 h (Fig. 3I). Furthermore, the administration of p65 siRNA down-regulated the expression of CCL4 in aged HAECs (Fig. 3J). Collectively, these findings indicate that CCL4 was up-regulated through the activation of p65, which was induced by ROS, to further amplify the downstream inflammatory proteins, such as IL-1β, IL-6, and TNF-α, in aged HAECs.

#### CCL4 inhibition reversed aging-induced cell dysfunction and increased angiogenic protein levels in aged HAECs

**Fig. 4:**
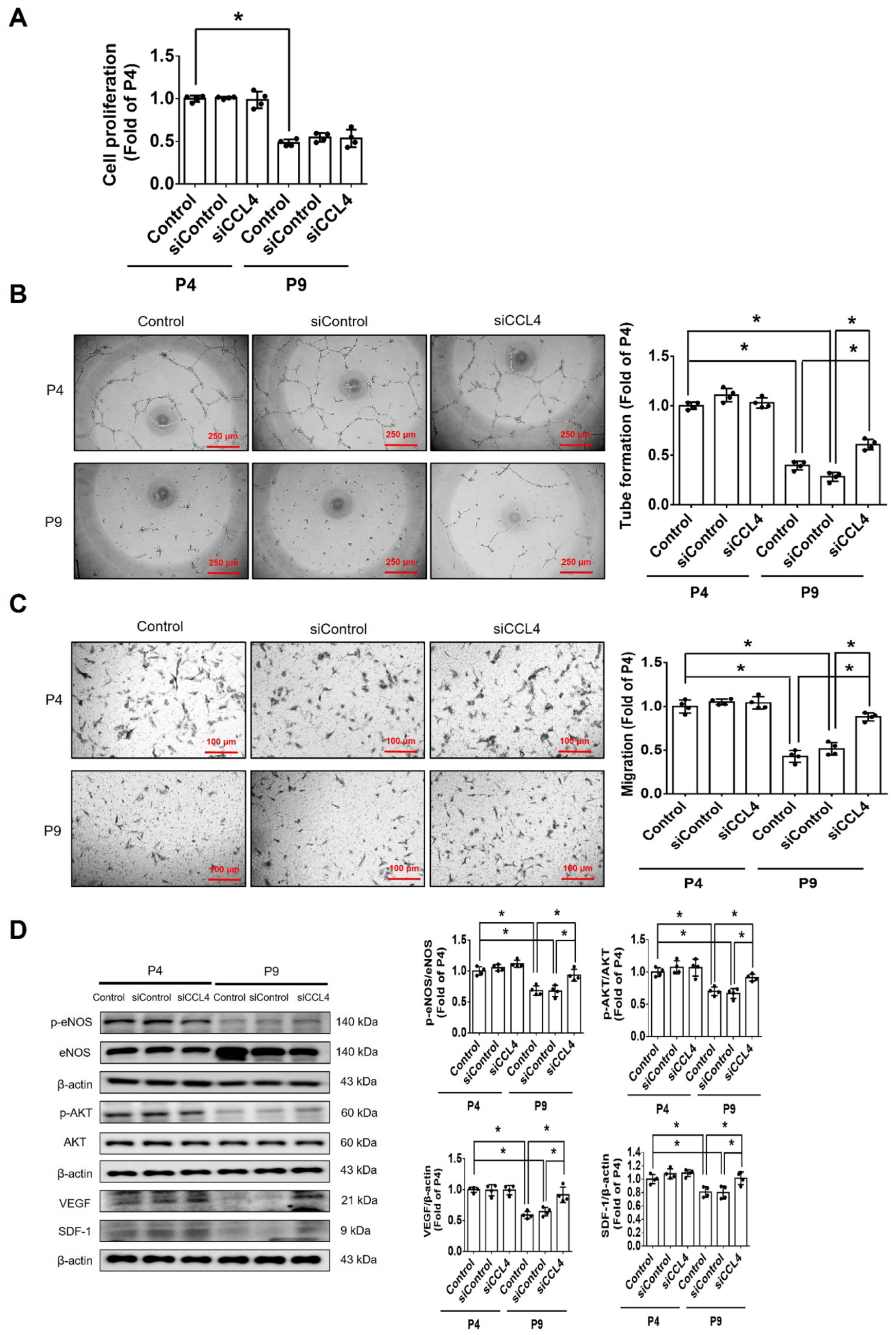
Inhibition of CCL4 reversed aging-induced cell dysfunction in HAECs. **A** Inhibition of CCL4 did not affect cell proliferation in HAECs (*n* = 4). **B** and **C** Inhibition of CCL4 improved tube formation and migration abilities in aged HAECs (*n* = 4). **D** Western blotting and statistical analysis of p-eNOS, p-AKT, VEGF, and SDF-1 expression in HAECs (*n* = 4). P4, passage 4; P9, passage 9; siCCL4, siRNA of CCL4. *P < 0.05, **P < 0.01

Cell proliferation ability was reduced in aged HAECs. CCL4 inhibition by siRNA did not affect cell proliferation in aged HAECs (Fig. 4A). Tube formation and migration abilities were attenuated in aged HAECs and were markedly improved by siCCL4 (Fig. 4B and C). The angiogenic factors, including p-eNOS, p-AKT, VEGF, and SDF-1, were reduced in aged HAECs and were up-regulated in the CCL4-inhibited group (Fig. 4D). The above findings indicate that the inhibition of CCL4 could reverse age-impaired cell functions and increase the levels of angiogenic proteins in aged HAECs.

### In vivo study

#### CCL4 deletion by genetic knockout reversed dermal aging and accelerated wound repair

**Fig. 5:**
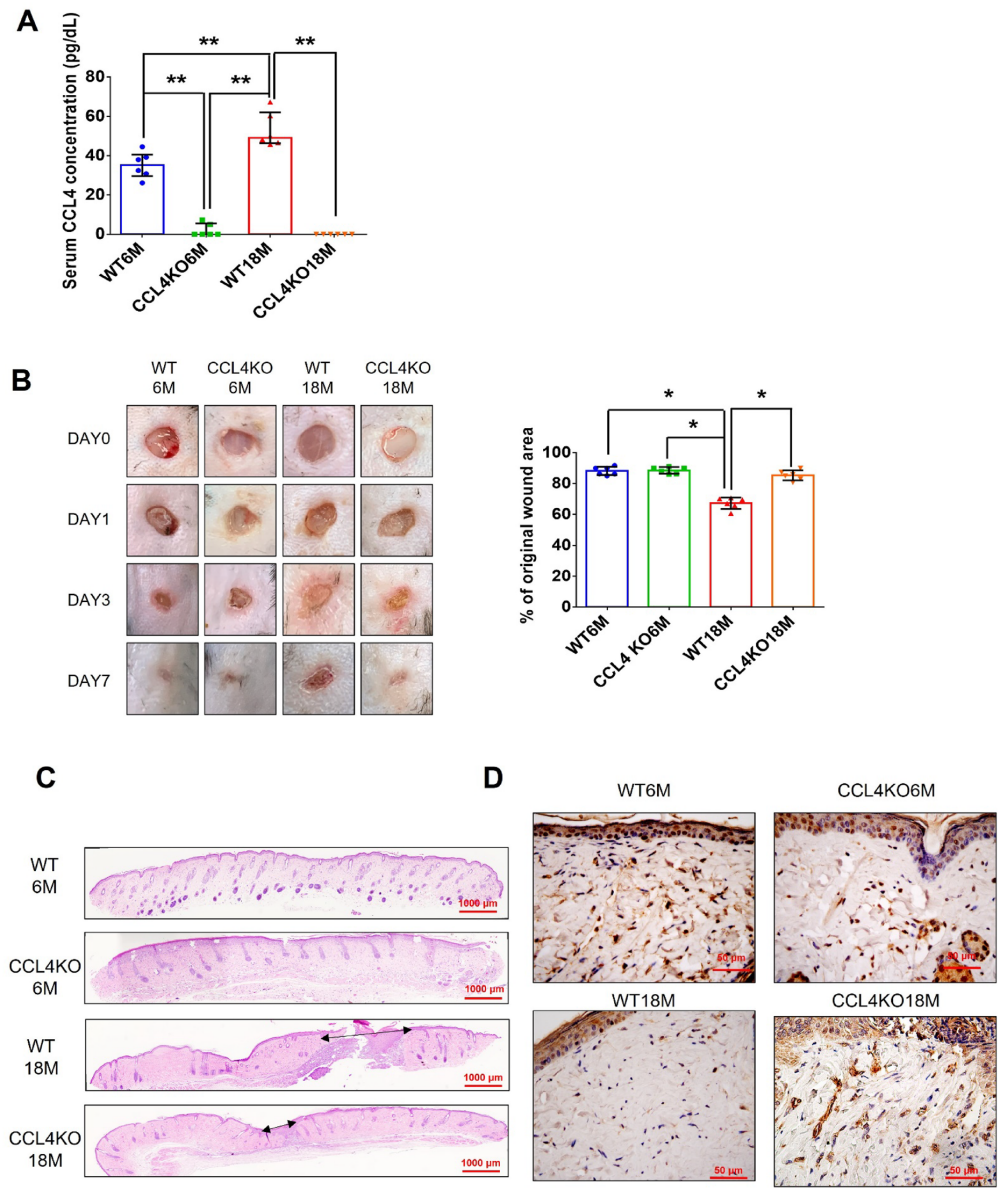

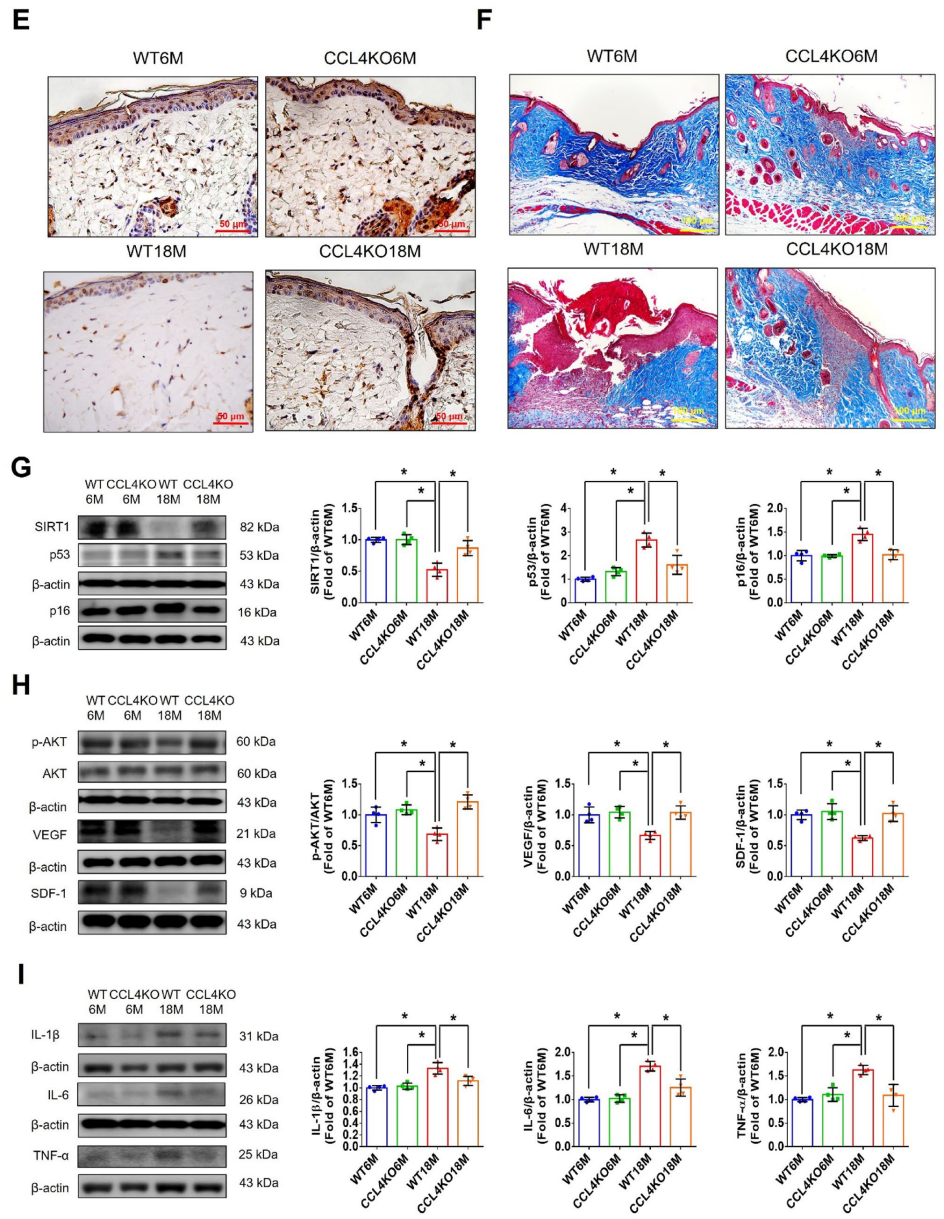
Deletion of CCL4 improved aging-induced delayed wound healing. **A** Aged mice have higher serum CCL4 concentrations compared to young mice (*n* = 6). **B** Representative photographs of wound healing in each group at various time points after wounding. The wound closure results were quantified on days 7 after wounding (*n* = 6). **C** Representative images with H&E staining. **D** and **E** Representative images with immunostaining of CD31 and Ki67. Deletion of CCL4 enhanced both CD31 and Ki67 positive areas in the 18 months old mice compared to the aged wild-type mice. **F** Representative images with Masson’s trichrome staining. Deletion of CCL4 enhanced collagen deposition in the 18 months old mice compared to the aged wild-type mice. **G** Western blotting and statistical analyses of aging factors, such as SIRT1, p53, and p16 (*n* = 4). **H** Western blotting and statistical analyses of angiogenic factors, such as p-AKT, VEGF, and SDF-1 (*n* = 4). **I** Western blotting and statistical analyses of inflammatory factors, such as IL-1β, IL-6, and TNF-α (*n* = 4). WT6M, wild-type mice at 6 months old; CCL4KO6M, CCL4 knockout mice at 6 months old; WT18M, wild-type mice at 18 months old; CCL4KO18M, CCL4 knockout mice at 18 months old. *P < 0.05, **P < 0.01

CCL4 knockout mice were used to confirm the novel role of CCL4 in aging-related vascular dysfunction in vivo. Serum CCL4 levels were up-regulated in aged (18-month-old) wild-type mice (WT18M) compared to the young (6-month-old) wild-type mice (WT6M). Both young and aged CCL4 knockout mice (CCL4KO6M and CCL4KO18M) had rarely circulating CCL4 (Fig. 5A). In the wound healing assay, the aged wild-type mice presented delayed wound repair post-injury compared to the young wild-type group. The aged CCL4 knockout mice showed an accelerated rate of wound closure compared to the aged wild-type mice (Fig. 5B). Improved wound healing in the aged CCL4 knockout mice was also observed in wound sections analyzed by H&E staining (Fig. 5C). Higher CD31 and Ki67 expressions, representing capillary density and cell proliferation, were detected in the aged CCL4 knockout mice compared to the aged wild-type mice (Fig. 5D and E). In addition, higher collagen accumulation in the wound area was detected in the aged CCL4 knockout mice than in the aged wild-type mice (Fig. 5F). Furthermore, the aging protein expressions of Sirtuin (SIRT) 1, p53, p16 were reversed (Fig. 5G); the angiogenic protein expressions of p-AKT, VEGF, and SDF-1 were increased (Fig. 5H); and the inflammatory protein expressions of IL-1β, IL-6, and TNF-α were decreased (Fig. 5I) in the wound area of the aged CCL4 knockout mice compared to that of the aged wild-type mice. In summary, CCL4 deletion exerted anti-dermal aging effects with enhanced angiogenesis and tissue repair in aged mice via the modulation of aging and inflammatory proteins.

#### CCL4 inhibition by genetic knockout improved angiogenesis in aged mice

**Fig. 6:**
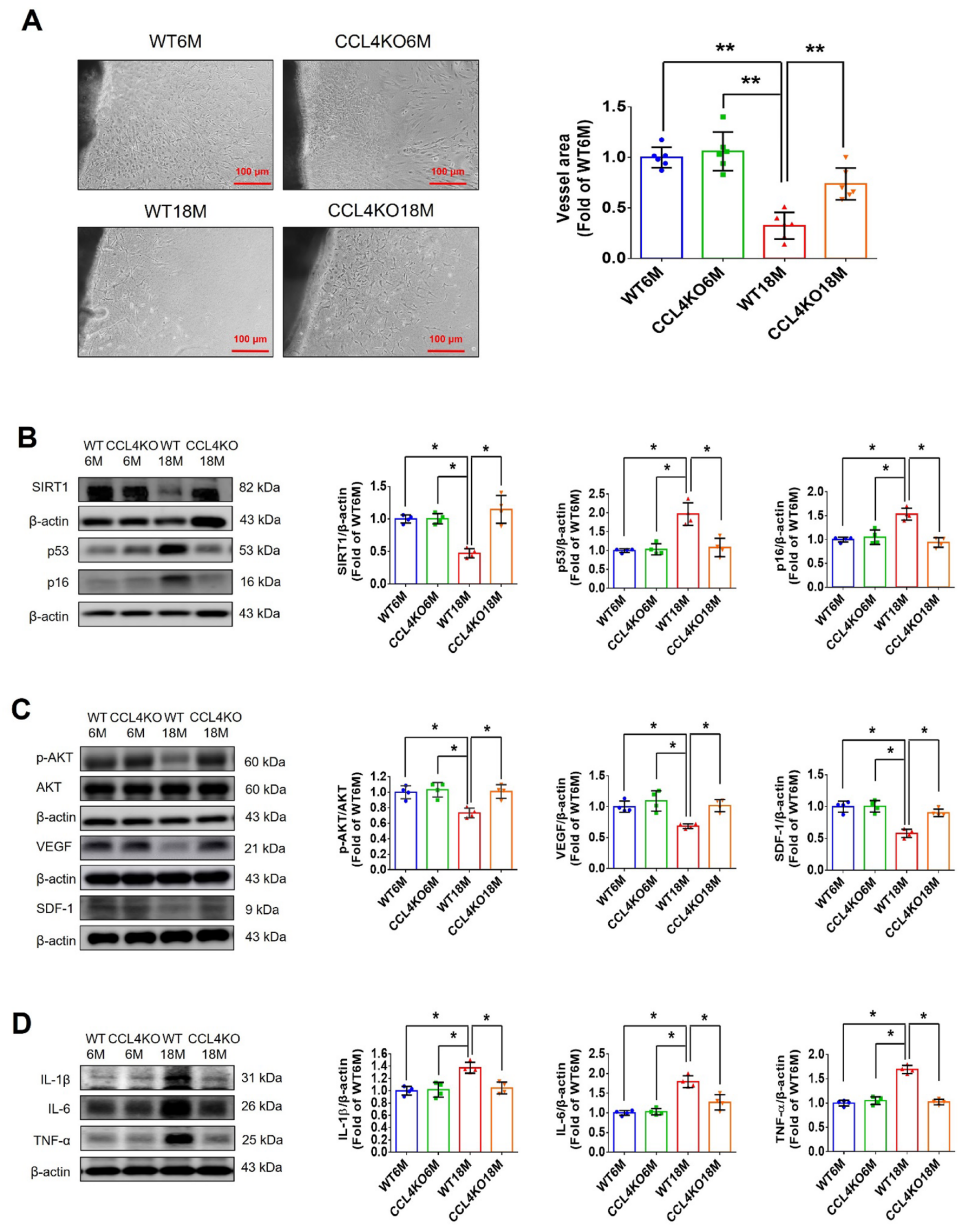

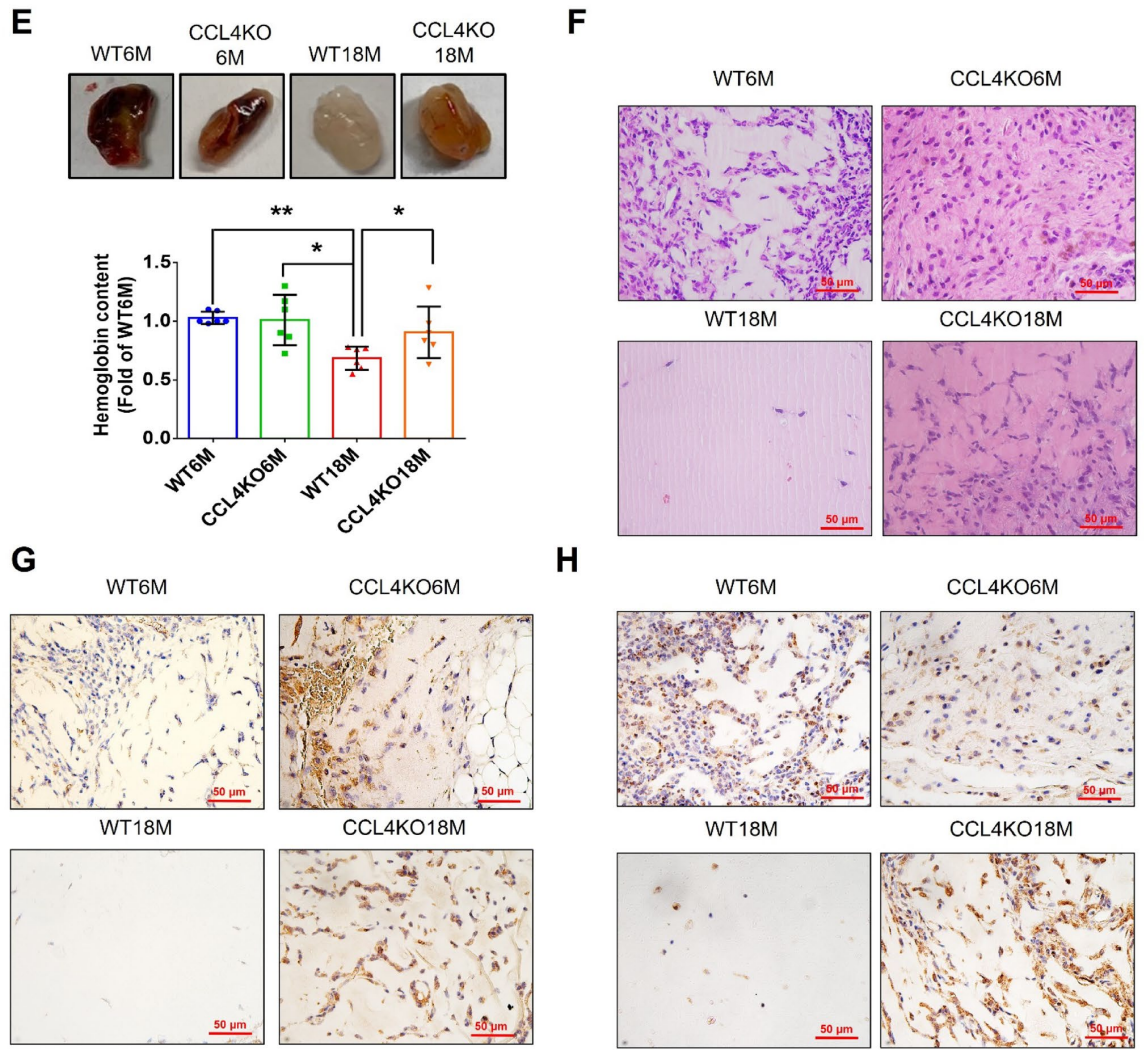
Deletion of CCL4 improved neovascularization in aged mice. **A** Representative images with endothelial sprouting in aortic rings. Aged CCL4 knockout mice had increased sprouting vessel number compared to the aged wild-type mice (*n* = 4). **B** Western blotting and statistical analyses of aging factors, such as SIRT1, p53, and p16 (*n* = 4). **C** Western blotting and statistical analyses of angiogenic factors, such as p-AKT, VEGF, and SDF-1 (*n* = 4). **D** Western blotting and statistical analyses of inflammatory factors, such as IL-1β, IL-6, and TNF-α (*n* = 4). **E** Representative Matrigel plug and the analysis of hemoglobin contents (*n* = 4). **F** Representative images with H&E staining. **G** and **H** Representative images with immunostaining of CD31 and Ki67. Deletion of CCL4 enhanced both CD31 and Ki67 positive areas in the 18 months old mice compared to the aged wild-type mice. WT6M, wild-type mice at 6 months old; CCL4KO6M, CCL4 knockout mice at 6 months old; WT18M, wild-type mice at 18 months old; CCL4KO18M, CCL4 knockout mice at 18 months old. *P < 0.05, **P < 0.01

Aortic sprouting was impaired in aortic rings from aged wild-type mice compared to those from young mice. Aortic rings from aged CCL4 knockout mice had a greater vessel sprouting number compared to those from aged wild-type mice (Fig. 6A). The expressions of aging, angiogenic, and inflammatory markers in aortic tissues were also evaluated. Aortas from aged CCL4 knockout mice had reversed aging proteins (SIRT1/p53/p16) (Fig. 6B), higher levels of angiogenic proteins (p-AKT/VEGF/SDF-1) (Fig. 6C), and lower levels of inflammatory proteins (IL-1β/IL-6/TNF-α) (Fig. 6D) compared to those from aged wild-type mice. In addition, we demonstrated the effect of CCL4 knockout on neovascularization by Matrigel plug assay in vivo. Lower vessel formation and hemoglobin content were observed in the plugs from aged wild-type mice compared to those from young mice. Plugs from aged CCL4 knockout mice showed increased vessel formation and hemoglobin content compared to those from aged wild-type mice (Fig. 6E). An enhanced vessel number in the plugs from aged CCL4 knockout mice was also observed in sections analyzed by H&E staining (Fig. 6F). Higher CD31 and Ki67 expressions in the plug were detected in the aged CCL4 knockout mice than in the aged wild-type mice (Fig. 6G and H). Taken together, CCL4 deletion improved neovascularization in aged mice via the modulation of aging and inflammatory proteins.

#### CCL4 inhibition accelerated wound repair and increased angiogenesis in aged mice

**Fig. 7:**
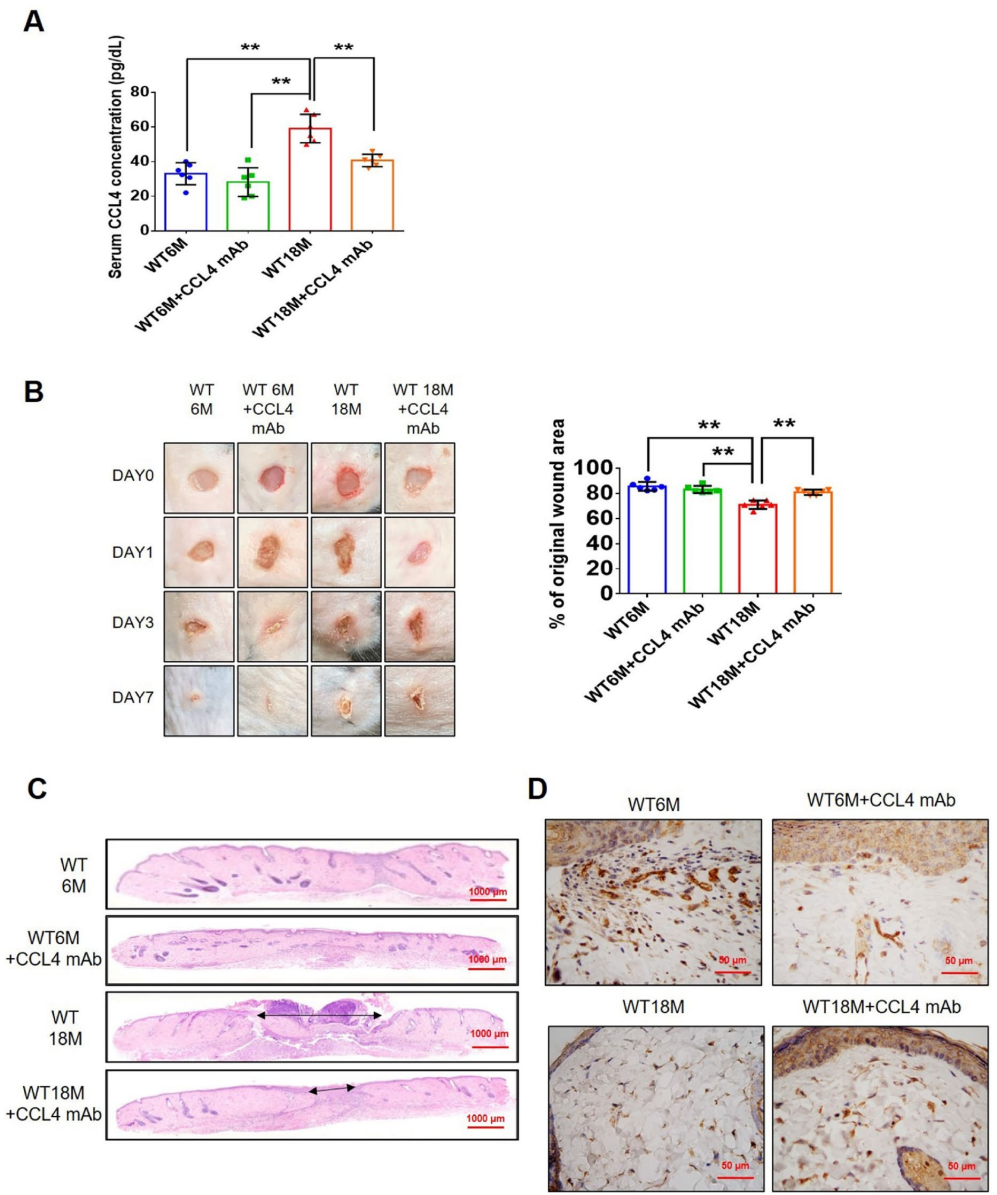

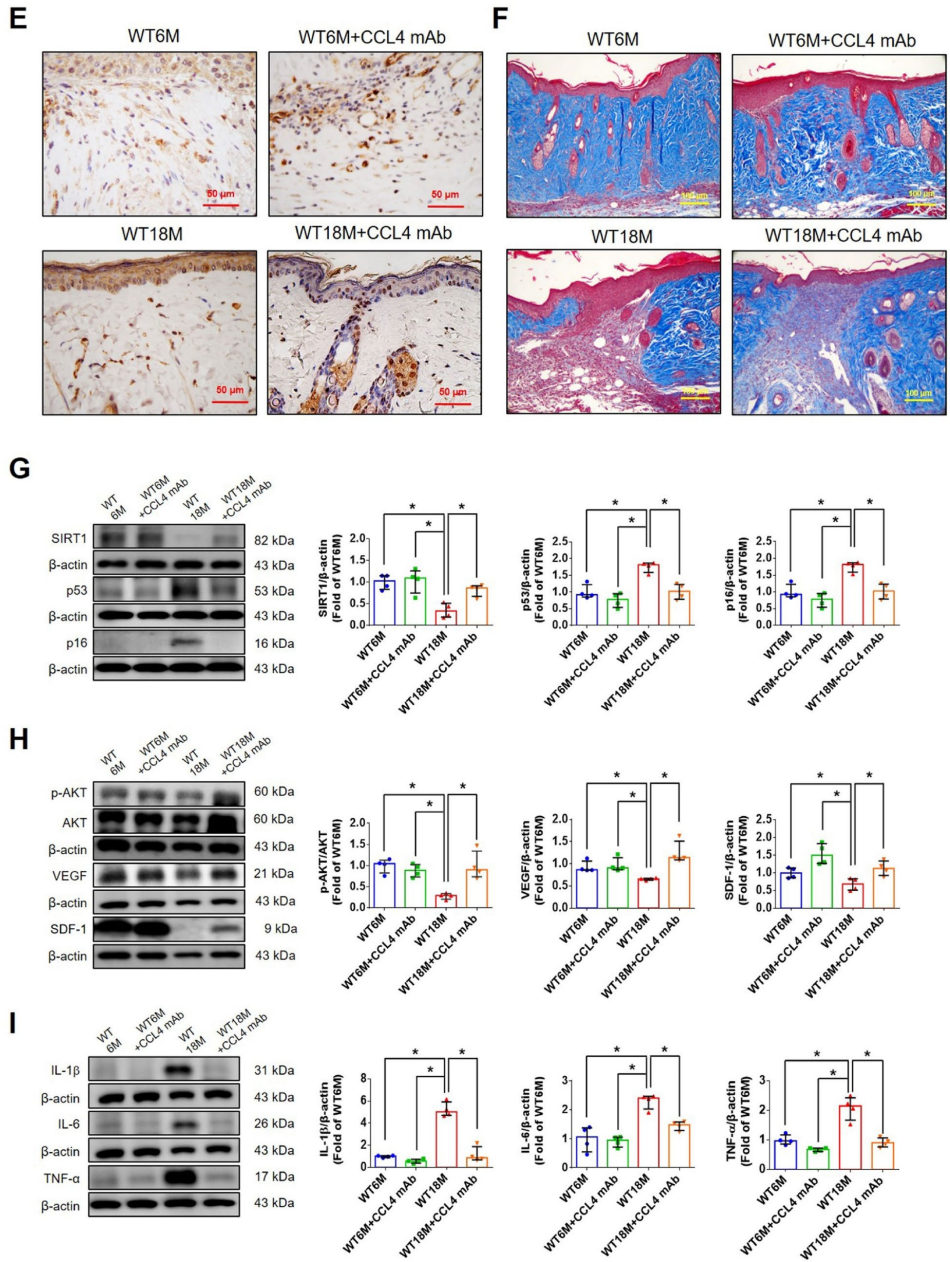

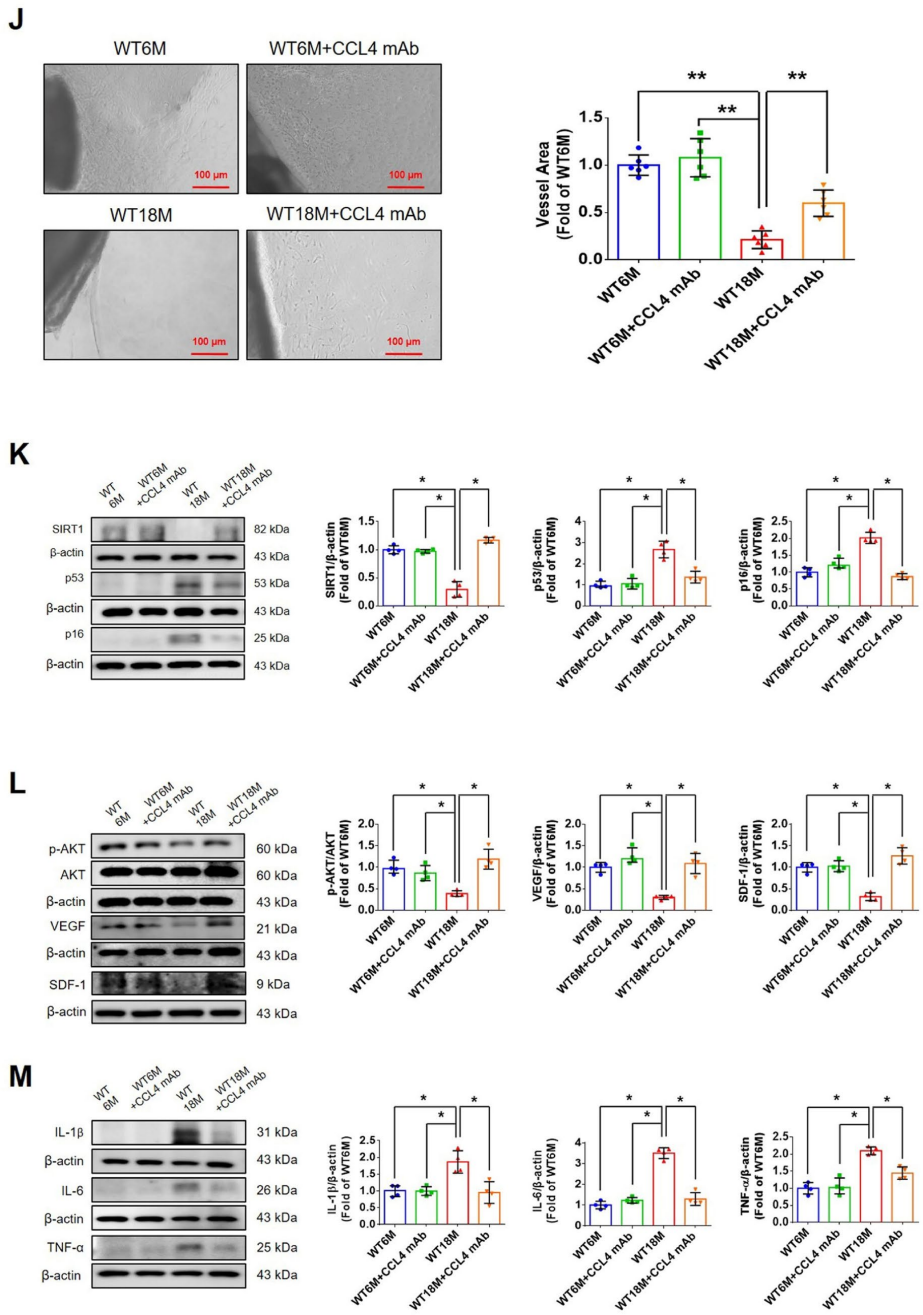

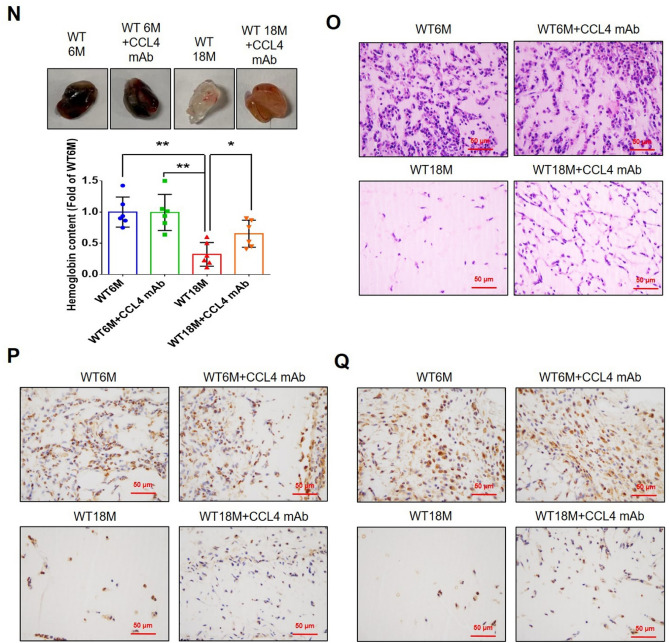
CCL4 inhibition improved wound healing and neovascularization in aged mice. **A** The CCL4 neutralizing antibody-injected group had lower serum CCL4 concentrations (*n* = 6). **B** Representative photographs of wound healing. The wound closure results were quantified on days 7 after wounding (*n* = 6). **C** and **O** Representative images with H&E staining. **D** and **P** Representative images with immunostaining of CD31. **E** and **Q** Representative images with immunostaining of Ki67. Inhibition of CCL4 enhanced both CD31 and Ki67 positive areas in the 18 months old mice. **F** Inhibition of CCL4 enhanced collagen deposition in the 18 months old mice. **G** and **K** Western blotting and statistical analyses of aging factors, such as SIRT1, p53, and p16 (*n* = 4). **H** and **L** Western blotting and statistical analyses of angiogenic factors, such as p-AKT, VEGF, and SDF-1 (*n* = 4). **I** and **M** Western blotting and statistical analyses of inflammatory factors, such as IL-1β, IL-6, and TNF-α (*n* = 4). **J** Aged mice treated with anti-CCL4 antibodies had increased sprouting vessel number (*n* = 4). **N** Representative Matrigel plug and the analysis of hemoglobin contents (*n* = 4). WT6M, wild-type mice at 6 months old; WT18M, wild-type mice at 18 months old. *P < 0.05, **P < 0.01

To determine whether CCL4 inhibition ameliorates aging-induced pathological vascular dysfunction, wild-type mice were treated with anti-CCL4 antibodies. Aged wild-type mice injected with CCL4 neutralizing antibodies had decreased circulating CCL4 levels (Fig. 7A). Aged mice received anti-CCL4 antibodies showed an accelerated rate of wound closure compared to the untreated aged wild-type mice (Fig. 7B). Improved wound repair in the aged mice treated with anti-CCL4 antibodies was observed in wound sections analyzed by H&E, CD31, Ki67, and collagen accumulation analyses (Fig. 7C and F).

Aortic rings from aged mice treated with anti-CCL4 antibodies had a greater vessel sprouting number (Fig. 7J). Tissue from wound area and aortas from aged mice treated with anti-CCL4 antibodies had reversed aging proteins (SIRT1/p53/p16) (Fig. 7G and K), higher levels of angiogenic proteins (p-AKT/VEGF/SDF-1) (Fig. 7H and L), and lower levels of inflammatory proteins (IL-1β/IL-6/TNF-α) (Fig. 7I and M).

Higher vessel formation and hemoglobin content were observed in the plugs from aged mice treated with anti-CCL4 antibodies (Fig. 7N). An enhanced vessel number in the plugs from aged mice treated with anti-CCL4 antibodies was observed in sections analyzed by H&E, CD31, and Ki67 staining (Fig. 7O and Q). Therefore, CCL4 inhibition by anti-CCL4 neutralizing antibody exerted anti-dermal aging and improved neovascularization effects in aged mice.

## Discussion

**Fig. 8:**
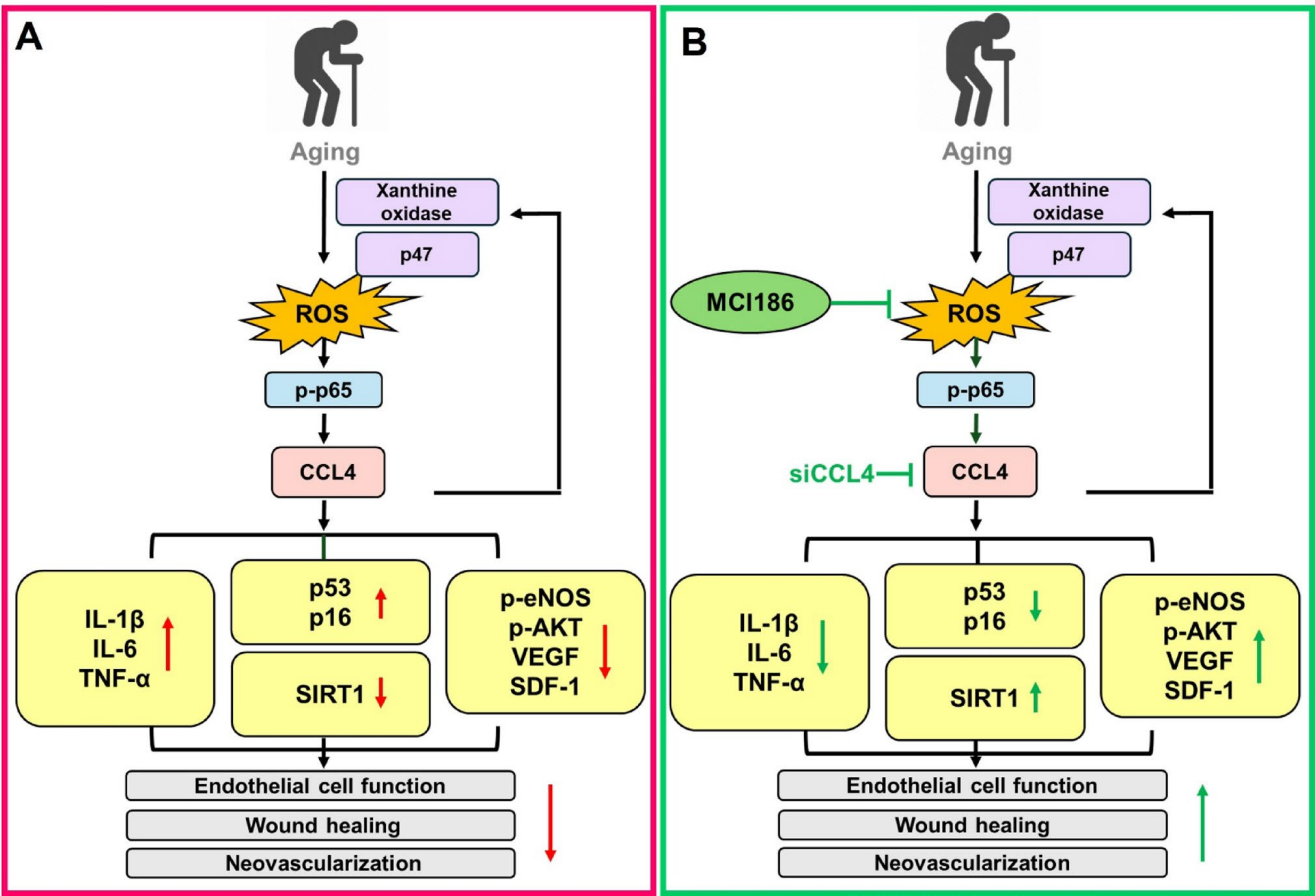
Summary of vital role of CCL4 in aging-impaired vascular diseases. **A** Aging related effect of ROS and CCL4 on the related the molecules and pathways and their effects on endothelial function. **B** The effects of CCL4 inhibition on the related the molecules and pathways and their effects on endothelial function. CCL4 may be a potential therapeutic target for vascular protections during aging

In the current study, there were several interesting findings relating to natural aging: (1) circulating levels of CCL4 were increased in elderly subjects, especially in those over 55 years of age; (2) primary cultured EPCs from elderly subjects had elevated expressions of CCL4 and the inhibition of CCL4 could reverse aging-induced inflammation and cell damage; (3) in vitro CCL4 inhibition reversed aging-related endothelial cell dysfunction through down-regulating ROS generation and inflammatory signaling pathways via p-p65/IL-1β/IL-6/TNF-α, and increasing the expression of angiogenic proteins via p-eNOS/p-AKT/VEGF/SDF-1; (4) the in vitro administration of CCL4 directly caused cell aging and ROS generation in HAECs, with increased expression of inflammatory proteins and reduced expression of angiogenic proteins; (5) in vivo CCL4 knockout/inhibition modulated tissue expression of senescent as well as inflammatory proteins, and promoted wound-repair and neovascularization in aged mice, thereby exerting anti-aging effects on dermal and vascular functions. Collectively, while CCL4 increased with aging and could induce endothelial inflammation, either intrinsic deficiency or external blockage of CCL4 may significantly attenuate aging related vascular impairment both in vitro and in vivo. These findings provide novel evidence for a mechanistic role of CCL4 in endothelial inflammation for vascular impairment in the natural aging process (Fig. 8).

Aging has a significant impact on the dysfunction of blood vessels, causing cardiovascular disease. Aging of the vasculature presents major risk factors for the development of atherosclerosis, hypertension, stroke, impaired angiogenesis, and arterial fibrillation, among others [16]. In the aging process, one of the most obvious phenomena is the up-regulation of ROS production [17]. While oxidative stress is a key inducer of endothelial senescence, levels of inflammatory cytokines, such as TNF-α could be increased with age [18, 19]. On the other hand, SIRTs are NAD^+^-dependent deacetylases, and the activation of SIRT1 leads to lifespan extension [20]. The up-regulation of antioxidant proteins, such as SIRT1, catalase, and manganese superoxide dismutase, could protect against insults from oxidative stress [21]. In addition, senescence is linked with the up-regulation of p53, p16, and senescence-associated β-galactosidase activity [22]. In this study, we showed that CCL4 treatment can lead to cell aging by enhancing oxidative stress and the phosphorylation of p65, and CCL4 blockade could decrease ROS production and reverse aging markers, including SIRT, p53, and p16. Our findings indicated that oxidative stress and p65 phosphorylation are mutually causal related to CCL4 expression, suggesting the direct involvement of CCL4 in natural vascular aging. Given the frequent comorbidities during aging, future studies may be required to elucidate the potential role of CCL4 in other aging models especially with the presence of various chronic diseases.

Angiogenesis is an important mechanism for repairing tissues in ischemic stroke, myocardial infraction, and ischemia. Interestingly, subepithelial angiogenesis may also contribute to skin wound healing, as indicated in the current study. Cellular senescence and excess ROS are leading causes of impaired angiogenesis in the elderly. The aging-related decrease in angiogenesis can be mediated by the down-regulation of VEGF [23]. On the other hand, EPCs can be mobilized from the bone marrow for vascular repair and neovascularization when vascular injury and tissue ischemia occur [24]. However, senescence reduces circulating numbers of EPCs—and their angiogenic activity—and is associated with aging-associated vascular diseases [25]. Kallistatin could decrease TNF-α-induced cellular senescence in EPCs, as indicated by decreased senescence-associated β-galactosidase activity [26]. Bradykinin could reduce increase the relative length of telomeres, reduce mitochondrial DNA copy numbers, and protect against EPC senescence induced by high glucose via PI3K-AKT-eNOS signaling pathways [27]. Furthermore, adiponectin-transfected EPCs have shown improved microvessel density in D-galactose-induced aging rats [28]. In the present study, the EPCs from both the young and elderly subjects, and both the young and old generations of HAECs were used for in vitro experiments. We demonstrated that CCL4 blockade can facilitate angiogenesis and protect vascular endothelial cells during aging via the up-regulation of p-eNOS/p-AKT/VEGF/SDF-1 signaling pathways in both primary cultured EPCs and HAECs. In addition, both the young and aged animals were used for in vivo experiments. The expression of angiogenic proteins were also increased in aortic and wounded dermal tissues in the aged CCL4 knockout mice or by the treatment of an anti-CCL4 neutralizing antibody. Importantly, CCL4 inhibition provides vascular and dermal anti-aging effects, accompanied by the promotion of neovascularization and wound healing. These data strongly suggest that CCL4 inhibition might be a potential strategy to improve aging related impairment of angiogenesis.

It has been suggested that angiogenesis may be divided into physiological and pathological angiogenesis. Physiological angiogenesis is mainly seen in wound repair and endometrial hyperplasia during the menstrual cycle, while pathological angiogenesis is mainly seen in tumors, chronic hepatitis, diabetes, etc. It is shown that angiogenesis could be enhanced in tumors and suppressed in diabetes, suggesting the varied mechanisms in different pathological conditions, tissues, and cell lines, leading to differential effects on neovascularization [29]. On the other hand, studies have shown that in diabetes, peripheral angiogenesis could be impaired even though the expression of VEGF is increased in ischemic muscles [30], whereas the pathological angiogenesis may be enhanced with increased expression of VEGF and SDF-1 in diabetic retinal endothelial cells [29]. These findings suggest that the same angiogenic factors may be associated with differential effects in different tissues even under the same pathological conditions [31, 32]. Interestingly, CCL4 has been found to promote p-MEK, p-ERK1/2, and p-STAT3 activation, leading to increased angiopoietin 2 expression, thereby facilitating angiogenesis in oral squamous cell carcinoma [33]. It seems that the potential effects of CCL4 on angiogenesis may be also different between pathological tumor angiogenesis and the natural aging as that shown in the present study. Further experiments are required to clarify the differential angiogenic role of CCL4 not only in physiological versus pathogenic conditions but also among different types of pathologies individually.

The levels of inflammatory cytokines and chemokines are increased and superoxide dismutase as well as glutathione peroxidase activities are decreased upon aging [13, 34]. Several signaling pathways have been identified that trigger the inflammatory process and stimulate the p65 and IL-1β-mediated inflammatory cascade of cytokines [35]. In fact, both inflammation and oxidative stress are the major factors of cell senescence. Inflammatory cytokines released from senescent cells further stimulate inflammation and senescence in the surrounding tissues [36]. Then, the amplified chronic inflammation may eventually lead to age-related diseases, especially cardiovascular diseases [3]. Moreover, enhanced levels of oxidative stress during physiological aging may promote EPC aging and cause endothelial dysfunction [37]. In this study, we confirmed the role of inflammation in the natural aging process and further showed that administration of CCL4 can lead to amplified down-stream inflammatory processes, including IL-1β/IL-6/TNFα. In addition, CCL4 inhibition provided anti-inflammatory effects in vascular aging. These data strongly suggest CCL4 inhibition could be a potential anti-aging strategy on account of its modulation of inflammation. Interestingly, given the similar benefits of systemic inhibition of CCL4 by genetical deletion and intraperitoneal injection of anti-CCL4 neutralizing antibodies in this study, their anti-aging effects may not be cell- or tissue-specific. Provided the multiple origins of CCL4 as previously indicated, the potential anti-aging effects of CCL4 inhibition in tissues other than vascular or dermal might be evaluated in future studies.

Our data are consistent with previous findings by ourselves and others. It has been suggested that microRNA-125b could modulate CCL4 expression in circulating immune cells—mainly monocytes and naïve CD8 T cells—and that its reduction could cause increases in CCL4 with age ^13^. In the current study, CCL4 was shown to be induced by intracellular oxidative stress via redox-dependent p65 signal pathways in aged endothelial cells. Our previous studies showed that CCL4 could also be induced in mononuclear cells, endothelial cells, and even EPCs, by various stimulations such as high glucose, lipids, cytokines and others via different pathways [11, 12]. Future studies of the aging process might therefore investigate the differing levels and impacts of CCL4 production in different cells and tissues. Furthermore, given the complex networks of inflammation and multiple mechanisms involved in the aging process, CCL4 may not be the sole contributor. The potential role of other inflammatory cytokines as well as chemokines and the potential interaction of CCL4 with them might be of interest in future studies.

In conclusion, the expression of CCL4 was increased in naturally aging subjects and in aged cells as well as animals, suggesting the general involvement of CCL4 in the aging process. In vitro administration of CCL4 directly induced vascular endothelial cell aging and dysfunction by generating ROS, amplifying inflammation, and reducing angiogenesis. CCL4 inhibition improved aging-related endothelial progenitors as well as endothelial cells inflammation and dysfunction in vitro. Both genetical deficiency of CCL4 and therapeutic administration of an anti-CCL4 neutralizing antibody provided vascular and dermal anti-aging effects accompanied by the promotion of neovascularization and wound healing in vivo, which may be possibly via the down-regulation of inflammatory proteins, such as IL-1β, IL-6, and TNF-α, and simultaneously the up-regulation of angiogenic proteins, such as p-eNOS, p-AKT, VEGF, and SDF-1. Accordingly, CCL4 could be critical to vascular dysfunction during the natural aging process by promoting endothelial oxidative stress and inflammation. Future clinical studies may seek to validate whether CCL4 inhibition could be a novel anti-aging strategy mainly for vascular protection.

## Supplementary Information

Below is the link to the electronic supplementary material.

Supplementary material 1 (DOCX 7802 kb)

## Data Availability

No datasets were generated or analysed during the current study.
