# Correction: CCL4 contributes to aging related angiogenic insufficiency through activating oxidative stress and endothelial inflammation

**DOI:** 10.1007/s10456-024-09964-2

**Published:** 2025-02-28

**Authors:** Ting-Ting Chang, Liang-Yu Lin, Ching Chen, Jaw-Wen Chen

**Affiliations:** 1 Department and Institute of Pharmacology, National Yang Ming Chiao Tung University, Taipei, Taiwan; 2 School of Medicine, National Yang Ming Chiao Tung University, Taipei, Taiwan; 3 Biomedical Industry Ph.D. Program, National Yang Ming Chiao Tung University, Taipei, Taiwan; 4 Cardiovascular Research Center, Taipei Medical University Hospital and Taipei Medical University, Taipei, Taiwan; 5 Division of Endocrinology and Metabolism, Department of Medicine, Taipei Veterans General Hospital, Taipei, Taiwan; 6 Division of Cardiology, Department of Medicine, Department of Research, Taipei Medical University Hospital, Taipei, Taiwan; 7 Division of Cardiology, Department of Medicine, Taipei Veterans General Hospital, Taipei, Taiwan; 8 Cardiovascular Research Center, National Yang Ming Chiao Tung University, Taipei, Taiwan; 9 Department and Institute of Pharmacology, School of Medicine, National Yang Ming Chiao Tung University, Taipei, Taiwan; 10 Department of Research, Taipei Medical University Hospital, Taipei, Taiwan

**Correction: Angiogenesis (2024) 27:475–499** 10.1007/s10456-024-09922-y

In the original published article, the images for the Control and siControl were inadvertently duplicated in Fig. 1J and the wrong x-axis group labeling of SIRT1 and p-AKT were made in Fig. 7G and H.

The incorrect and corrected versions of Figs. 1 and 7 were provided in this correction.

The original article has been corrected.

Incorrect version of figures 1 and 7:

**Fig. 1:**
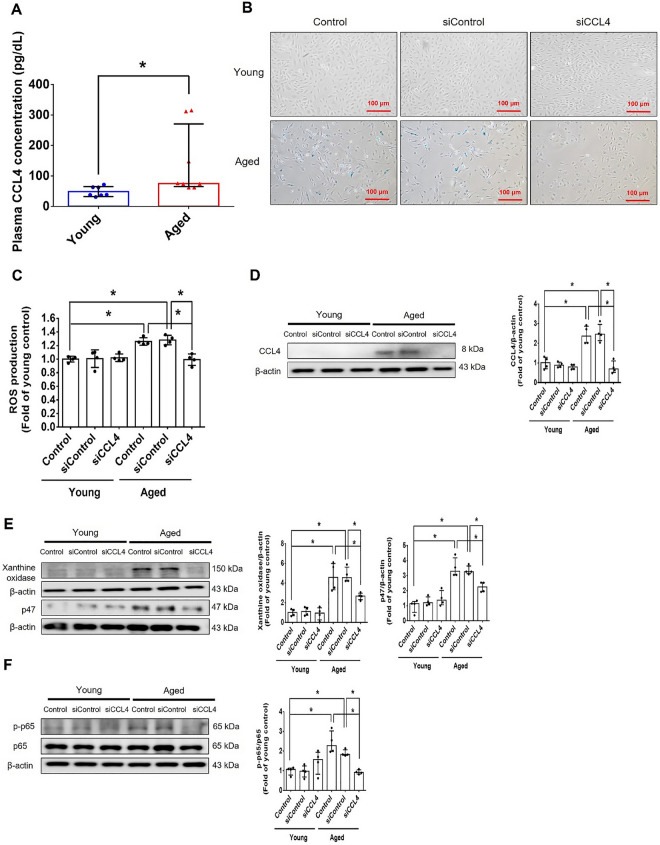

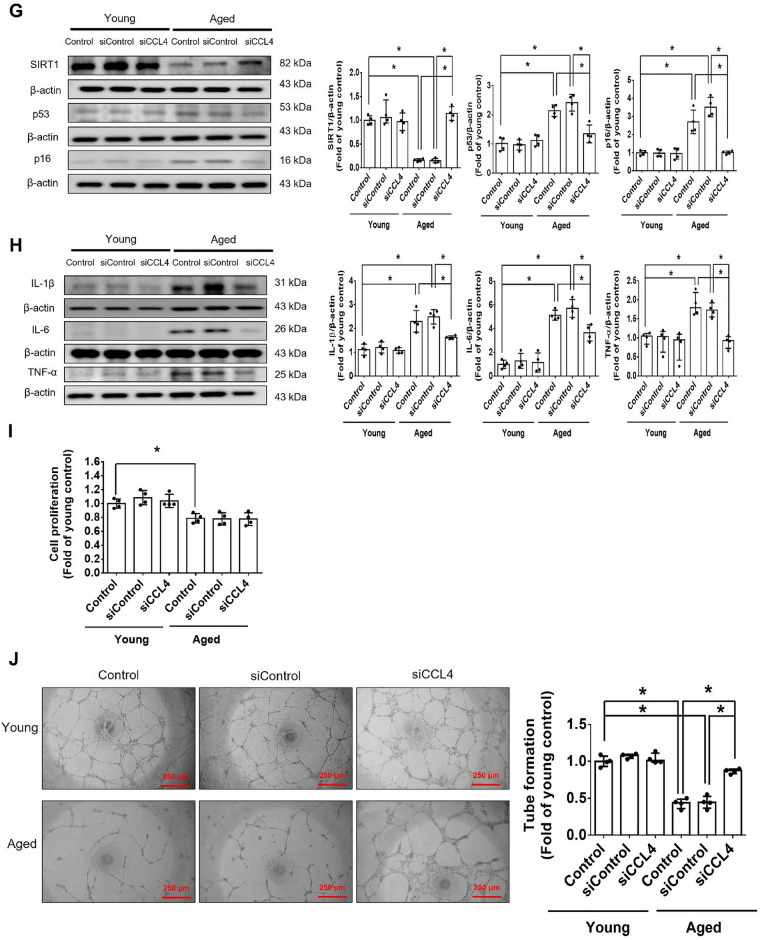

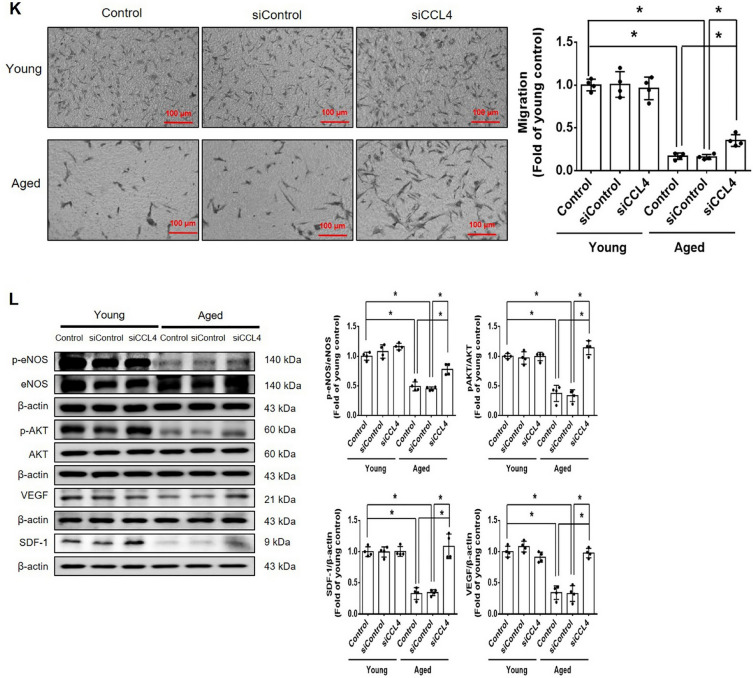
Inhibition of CCL4 reversed cell aging and inflammation in EPCs from aged subjects. **A** Elderly subjects (> 55 years old; *n* = 8) had higher plasma CCL4 concentrations compared to the young subjects (< 30 years old; *n* = 7). **B** Inhibition of CCL4 reduced senescence of EPCs from aged subjects. **C** Inhibition of CCL4 reduced ROS productions in EPCs from aged subjects (*n* = 4). **D** and **F** Western blotting and statistical analysis of CCL4, xanthine oxidase, p47, and p-p65 in primary cultured EPCs (*n* = 4). **G** and **H** Western blotting and statistical analysis of SIRT1, p53, p16, IL-1β, IL-6, and TNF-α expression in primary cultured EPCs (*n* = 4). **I** Inhibition of CCL4 did not affect cell proliferation in HAECs (*n* = 4). **J** and **K** Inhibition of CCL4 improved tube formation and migration abilities in EPCs from aged subjects (*n* = 4). **L** Western blotting and statistical analysis of eNOS, p-AKT, VEGF, and SDF-1 expression in primary cultured EPCs (*n* = 4). *P < 0.05, **P < 0.01

**Fig. 7:**
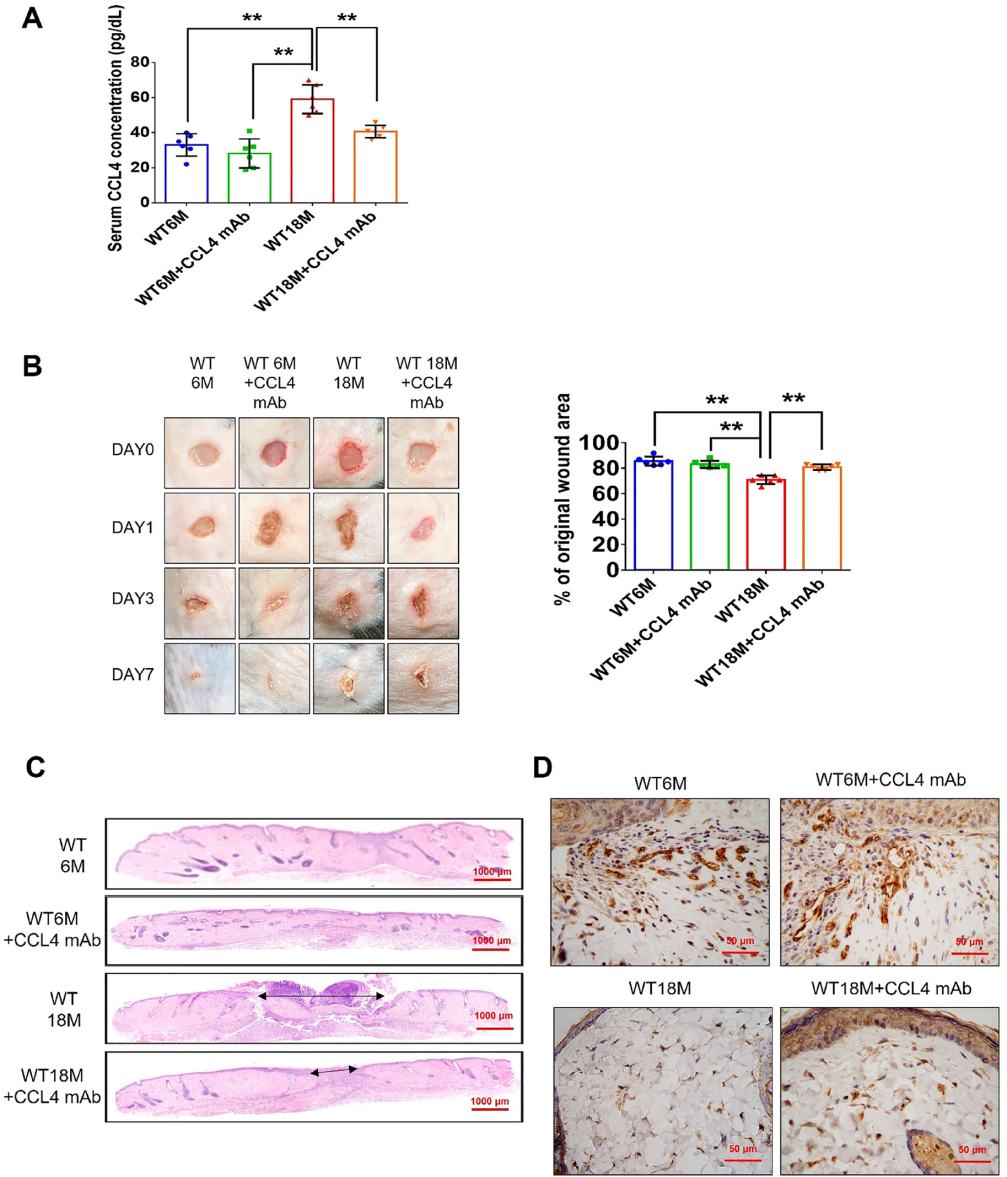

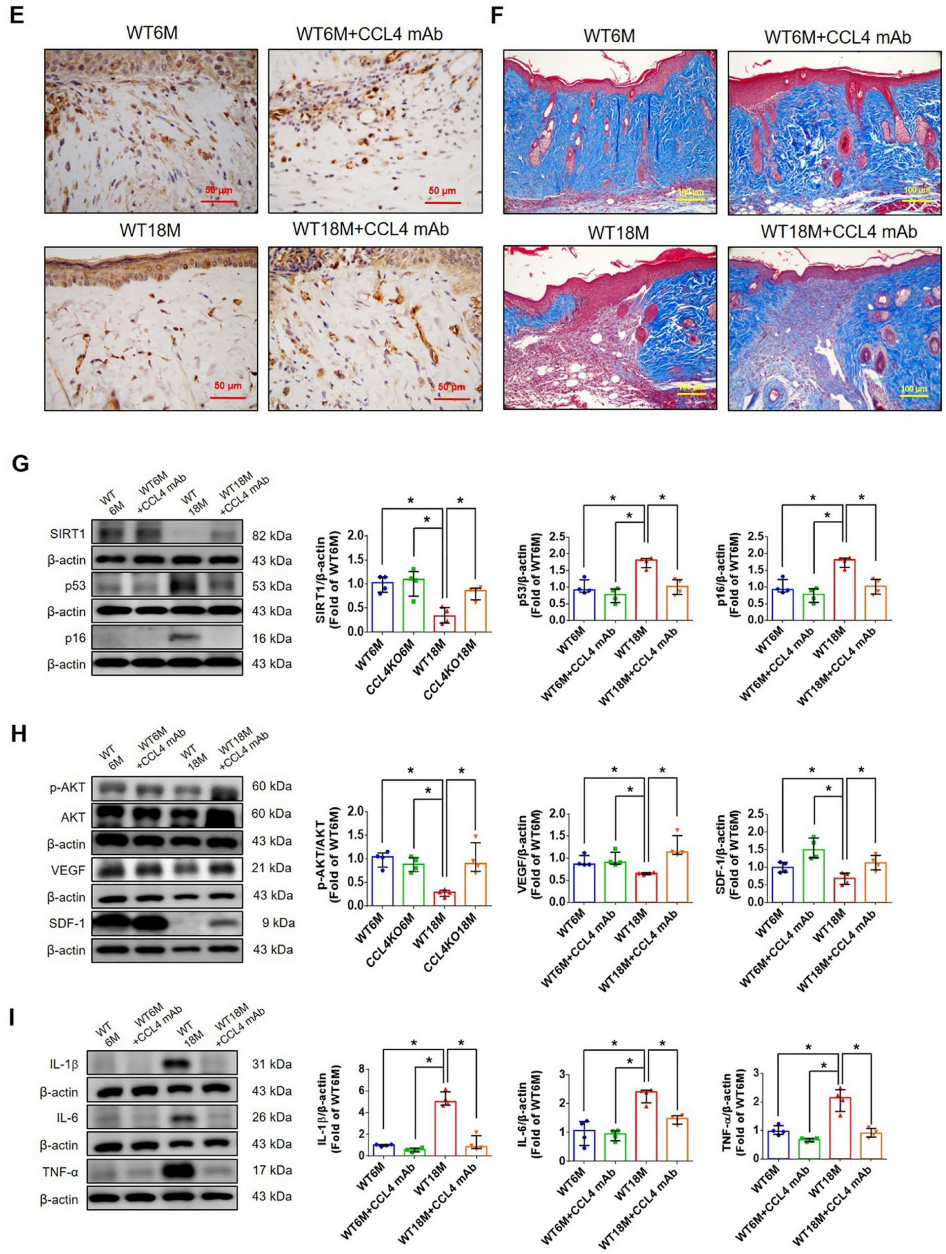

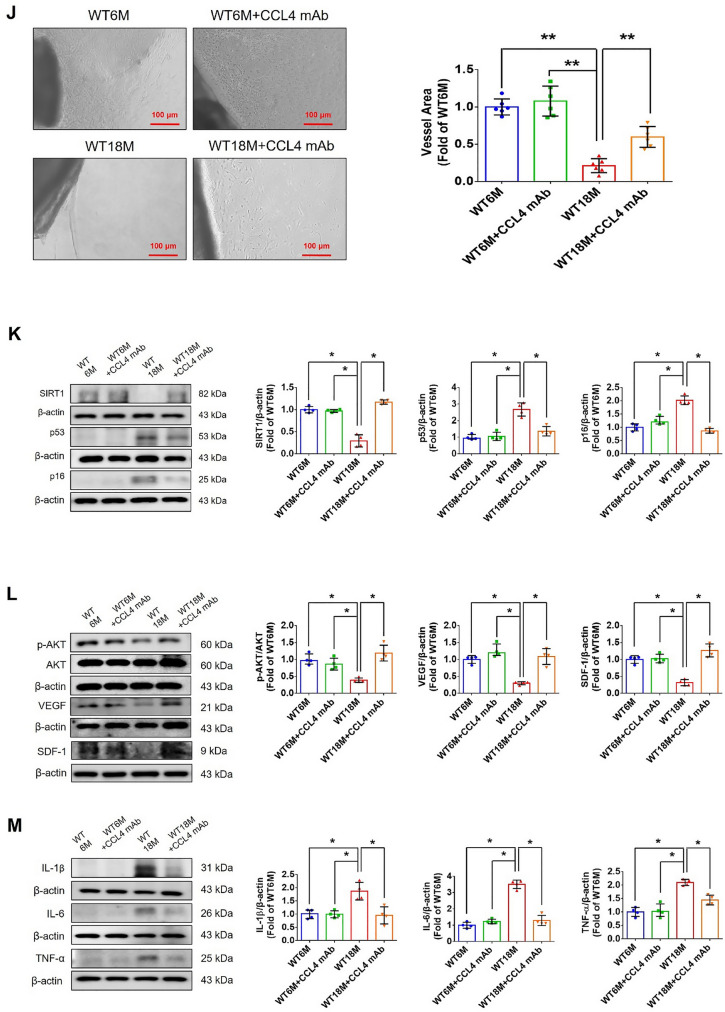

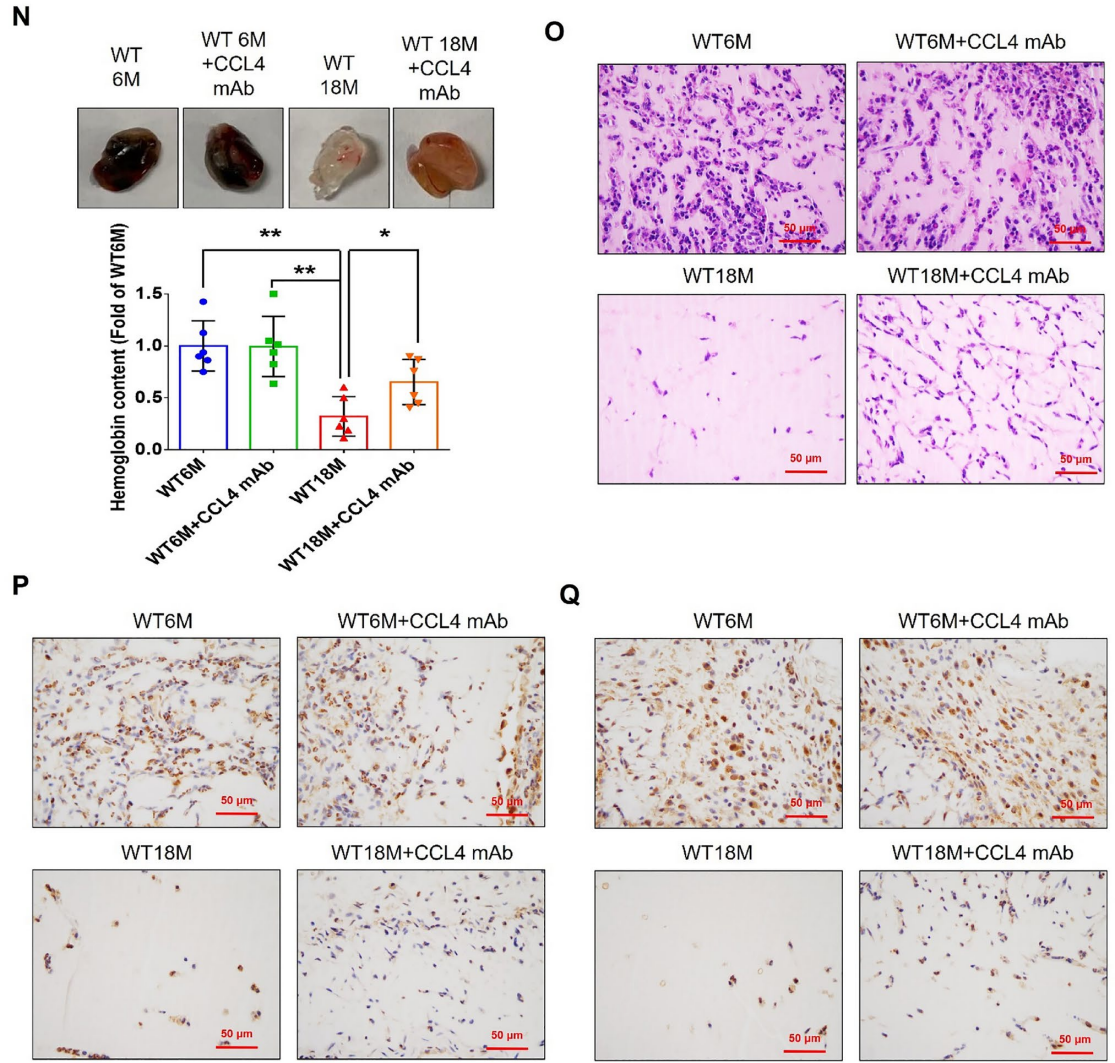
CCL4 inhibition improved wound healing and neovascularization in aged mice. **A** The CCL4 neutralizing antibody-injected group had lower serum CCL4 concentrations (*n* = 6). **B** Representative photographs of wound healing. The wound closure results were quantified on days 7 after wounding (*n* = 6). **C** and **O** Representative images with H&E staining. **D** and **P** Representative images with immunostaining of CD31. **E** and **Q** Representative images with immunostaining of Ki67. Inhibition of CCL4 enhanced both CD31 and Ki67 positive areas in the 18 months old mice. **F** Inhibition of CCL4 enhanced collagen deposition in the 18 months old mice. **G** and **K** Western blotting and statistical analyses of aging factors, such as SIRT1, p53, and p16 (*n* = 4). **H** and **L** Western blotting and statistical analyses of angiogenic factors, such as p-AKT, VEGF, and SDF-1 (*n* = 4). **I** and **M** Western blotting and statistical analyses of inflammatory factors, such as IL-1β, IL-6, and TNF-α (*n* = 4). **J** Aged mice treated with anti-CCL4 antibodies had increased sprouting vessel number (*n* = 4). **N** Representative Matrigel plug and the analysis of hemoglobin contents (*n* = 4). WT6M, wild-type mice at 6 months old; WT18M, wild-type mice at 18 months old. *P < 0.05, **P < 0.01

Corrected version of figures 1 and 7:

**Fig. 1:**
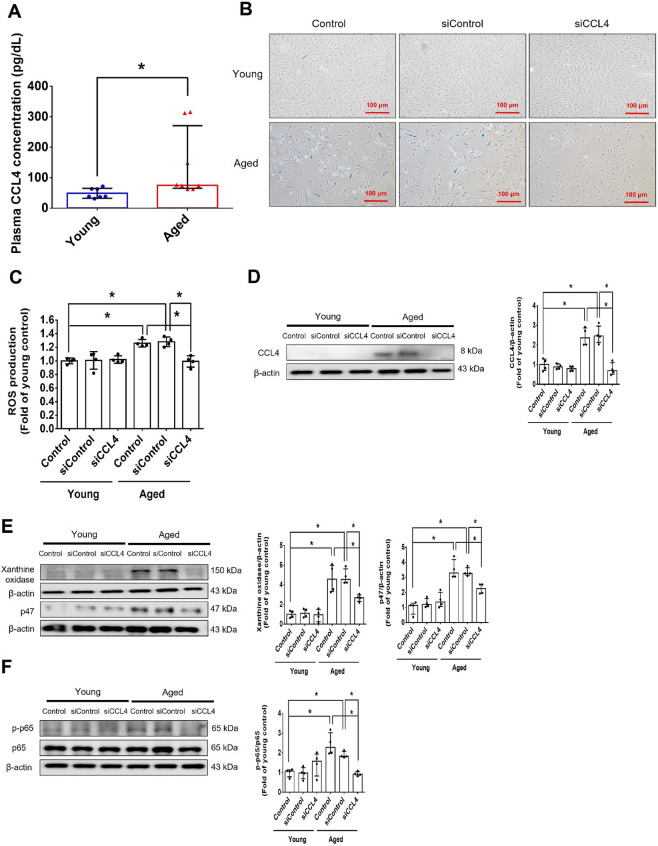

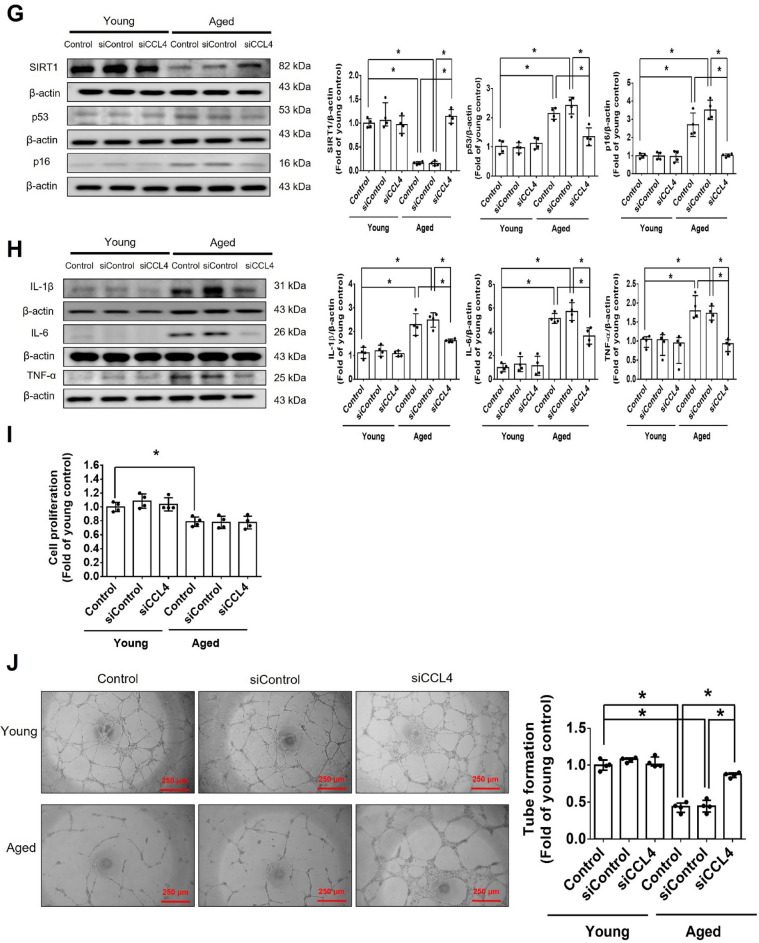

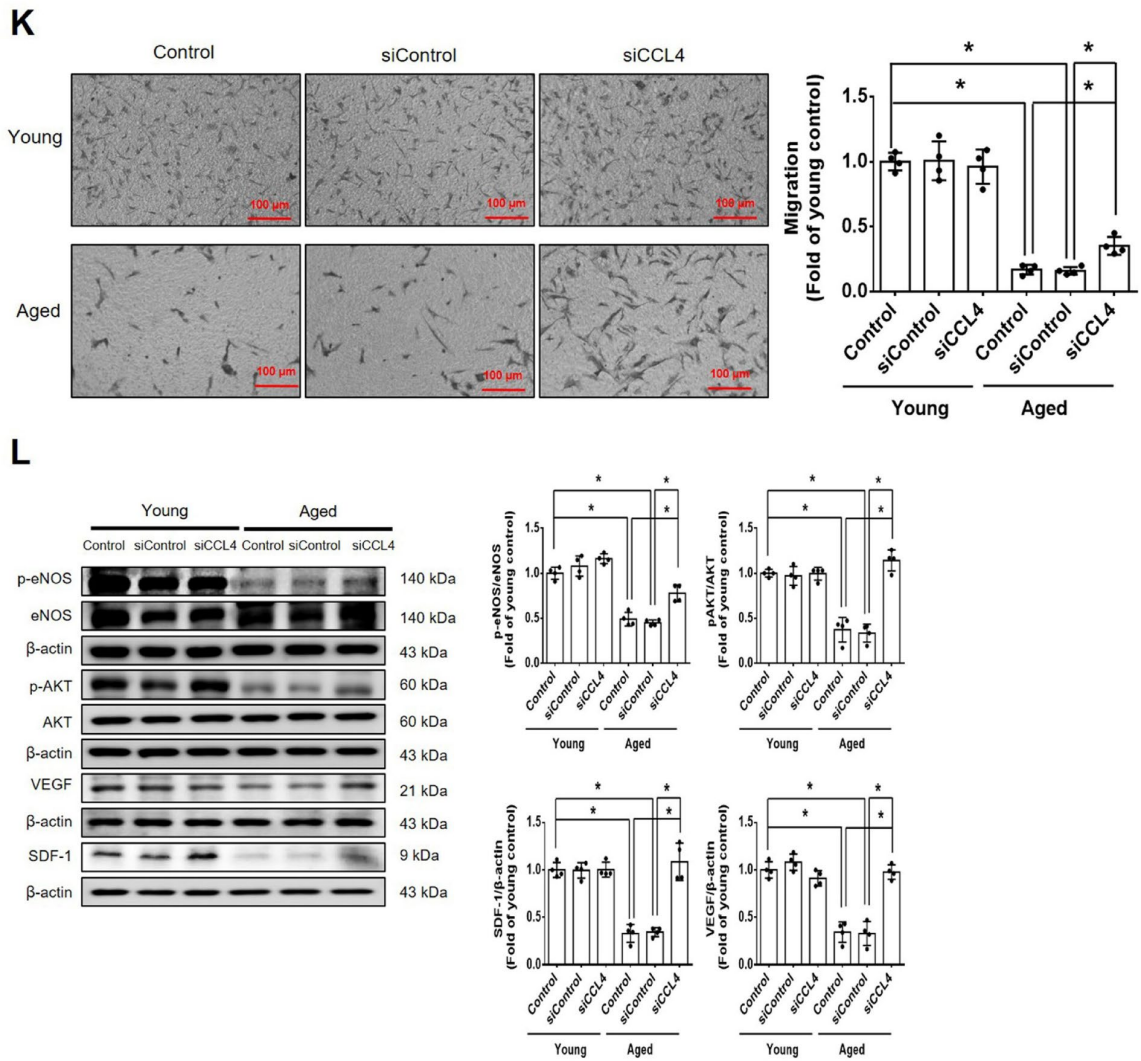
Inhibition of CCL4 reversed cell aging and inflammation in EPCs from aged subjects. **A** Elderly subjects (> 55 years old; *n* = 8) had higher plasma CCL4 concentrations compared to the young subjects (< 30 years old; *n* = 7). **B** Inhibition of CCL4 reduced senescence of EPCs from aged subjects. **C** Inhibition of CCL4 reduced ROS productions in EPCs from aged subjects (*n* = 4). **D** and **F** Western blotting and statistical analysis of CCL4, xanthine oxidase, p47, and p-p65 in primary cultured EPCs (*n* = 4). **G** and **H** Western blotting and statistical analysis of SIRT1, p53, p16, IL-1β, IL-6, and TNF-α expression in primary cultured EPCs (*n* = 4). **I** Inhibition of CCL4 did not affect cell proliferation in HAECs (*n* = 4). **J** and **K** Inhibition of CCL4 improved tube formation and migration abilities in EPCs from aged subjects (*n* = 4). **L** Western blotting and statistical analysis of eNOS, p-AKT, VEGF, and SDF-1 expression in primary cultured EPCs (*n* = 4). *P < 0.05, **P < 0.01

**Fig. 7:**
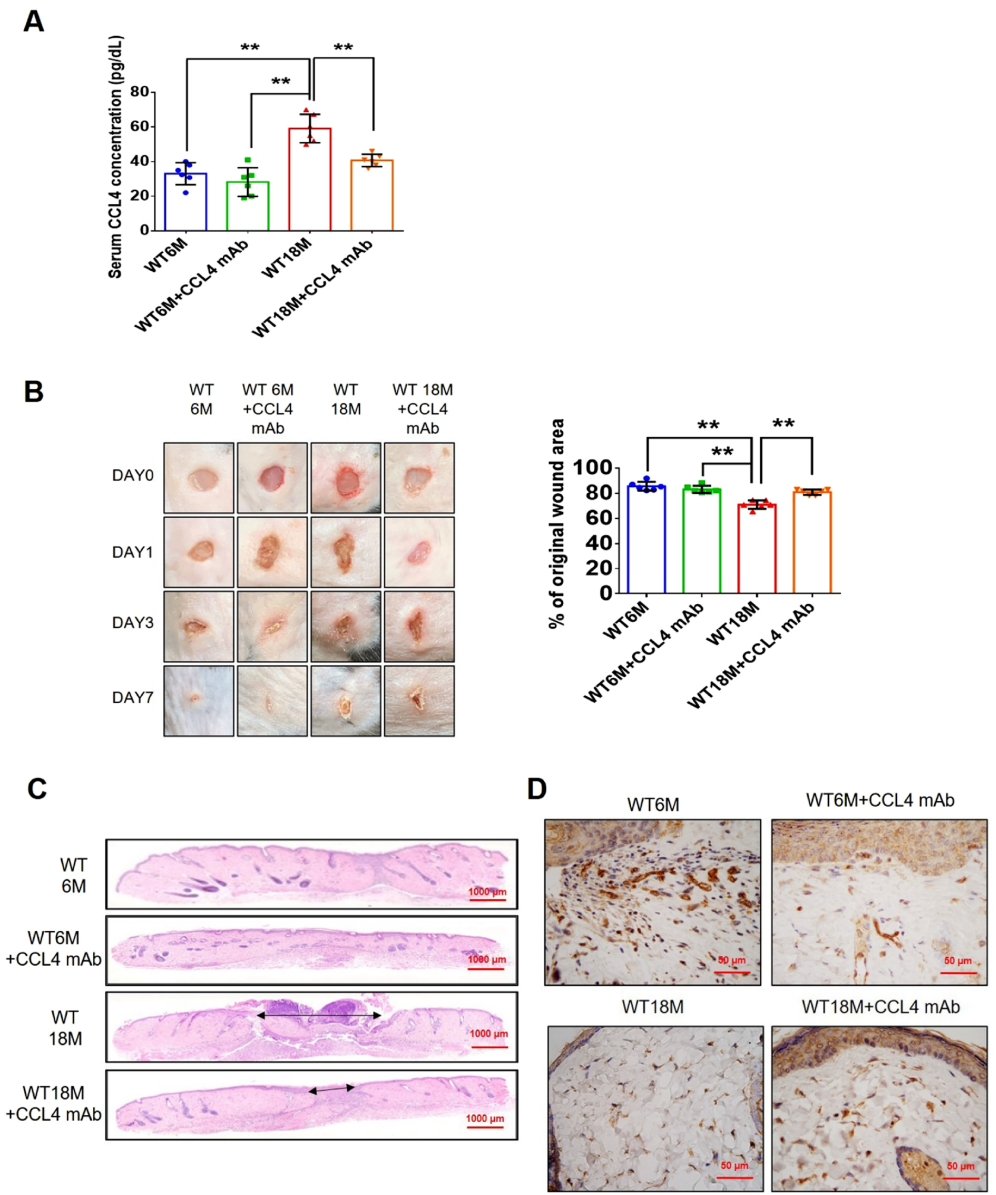

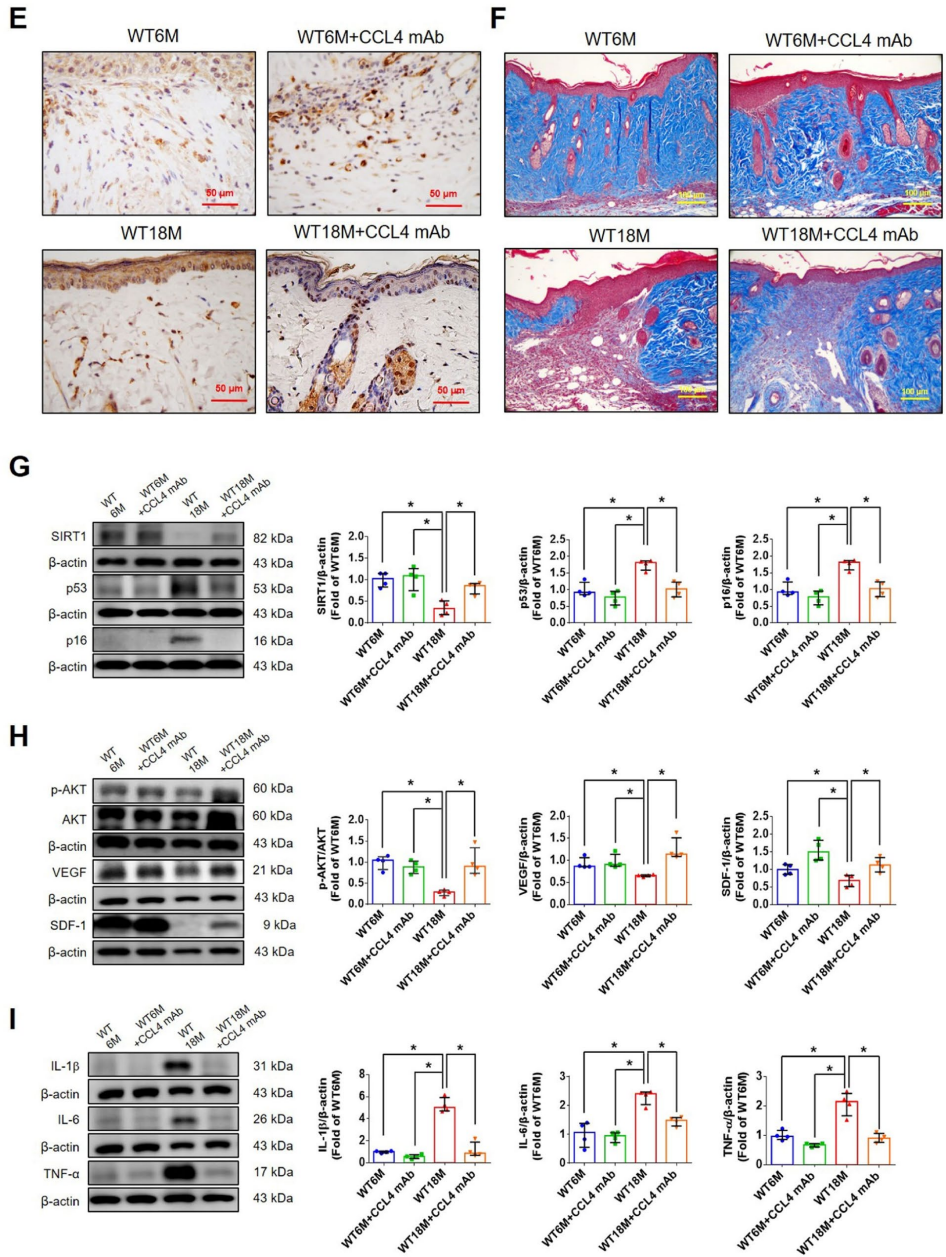

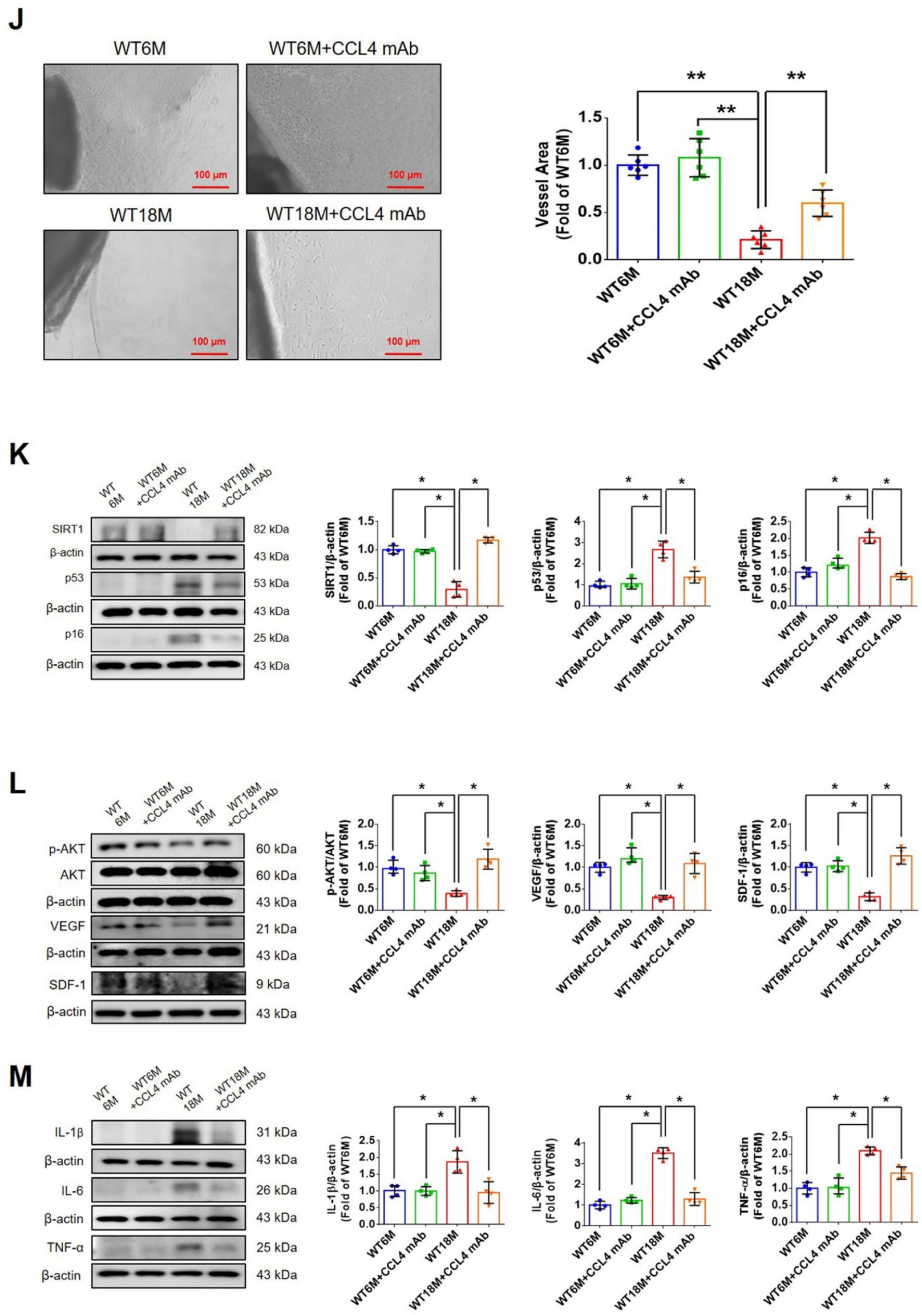

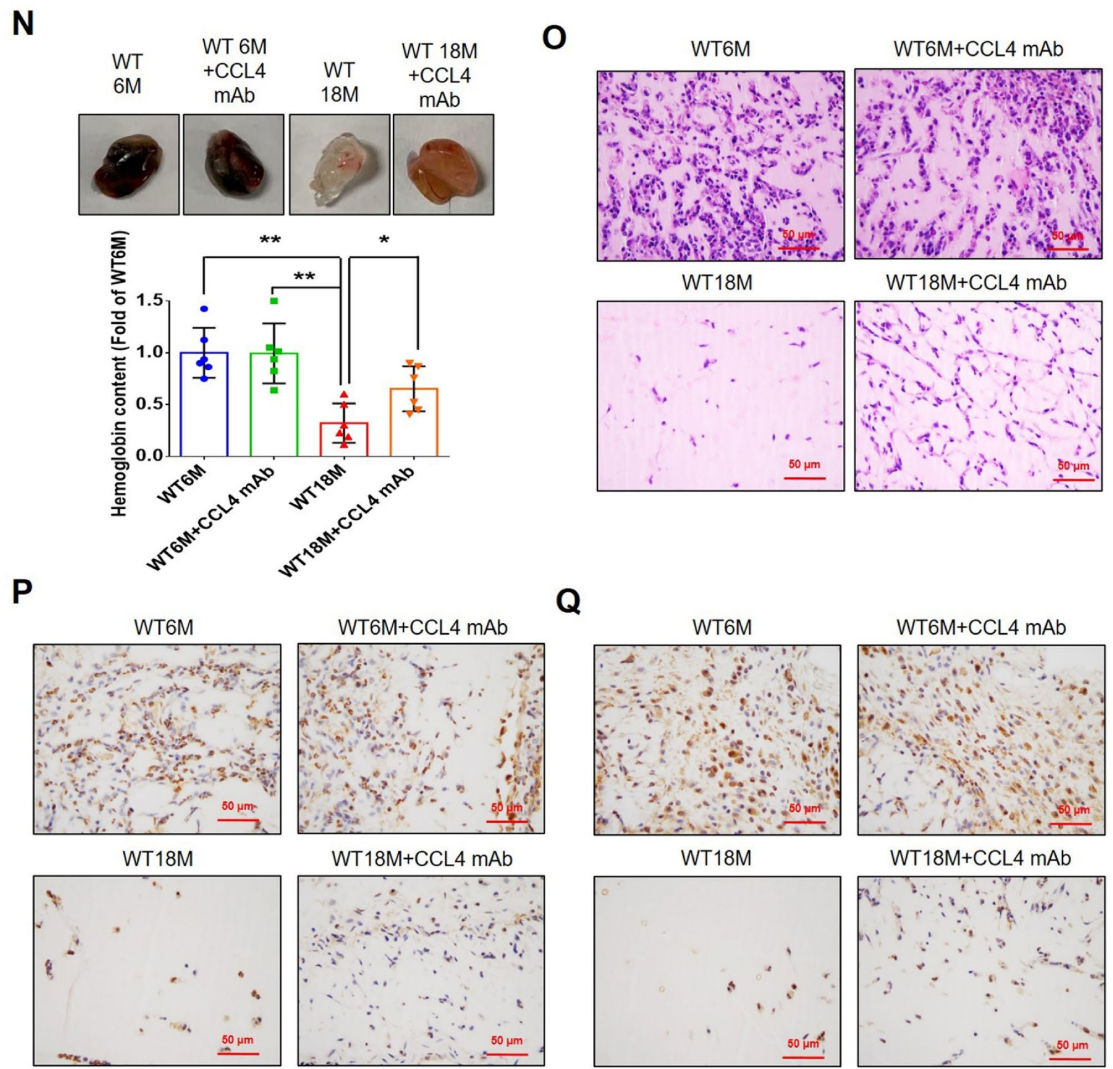
CCL4 inhibition improved wound healing and neovascularization in aged mice. **A** The CCL4 neutralizing antibody-injected group had lower serum CCL4 concentrations (*n* = 6). **B** Representative photographs of wound healing. The wound closure results were quantified on days 7 after wounding (*n* = 6). **C** and **O** Representative images with H&E staining. **D** and **P** Representative images with immunostaining of CD31. **E** and **Q** Representative images with immunostaining of Ki67. Inhibition of CCL4 enhanced both CD31 and Ki67 positive areas in the 18 months old mice. **F** Inhibition of CCL4 enhanced collagen deposition in the 18 months old mice. **G** and **K** Western blotting and statistical analyses of aging factors, such as SIRT1, p53, and p16 (*n* = 4). **H** and **L** Western blotting and statistical analyses of angiogenic factors, such as p-AKT, VEGF, and SDF-1 (*n* = 4). **I** and **M** Western blotting and statistical analyses of inflammatory factors, such as IL-1β, IL-6, and TNF-α (*n* = 4). **J** Aged mice treated with anti-CCL4 antibodies had increased sprouting vessel number (*n* = 4). **N** Representative Matrigel plug and the analysis of hemoglobin contents (*n* = 4). WT6M, wild-type mice at 6 months old; WT18M, wild-type mice at 18 months old. *P < 0.05, **P < 0.01

