# Supplementary material for: CCL4 contributes to aging related angiogenic insufficiency through activating oxidative stress and endothelial inflammation
Source: Angiogenesis. 2024 May 13;27(3):475–99. doi: 10.1007/s10456-024-09922-y (PMC11303582; doi:10.1007/s10456-024-09922-y)
Supplement: Supplementary file 1 — Supplementary material 1 (DOCX 7802 kb) [file 10456_2024_9922_MOESM1_ESM.docx]

**Figure S1**


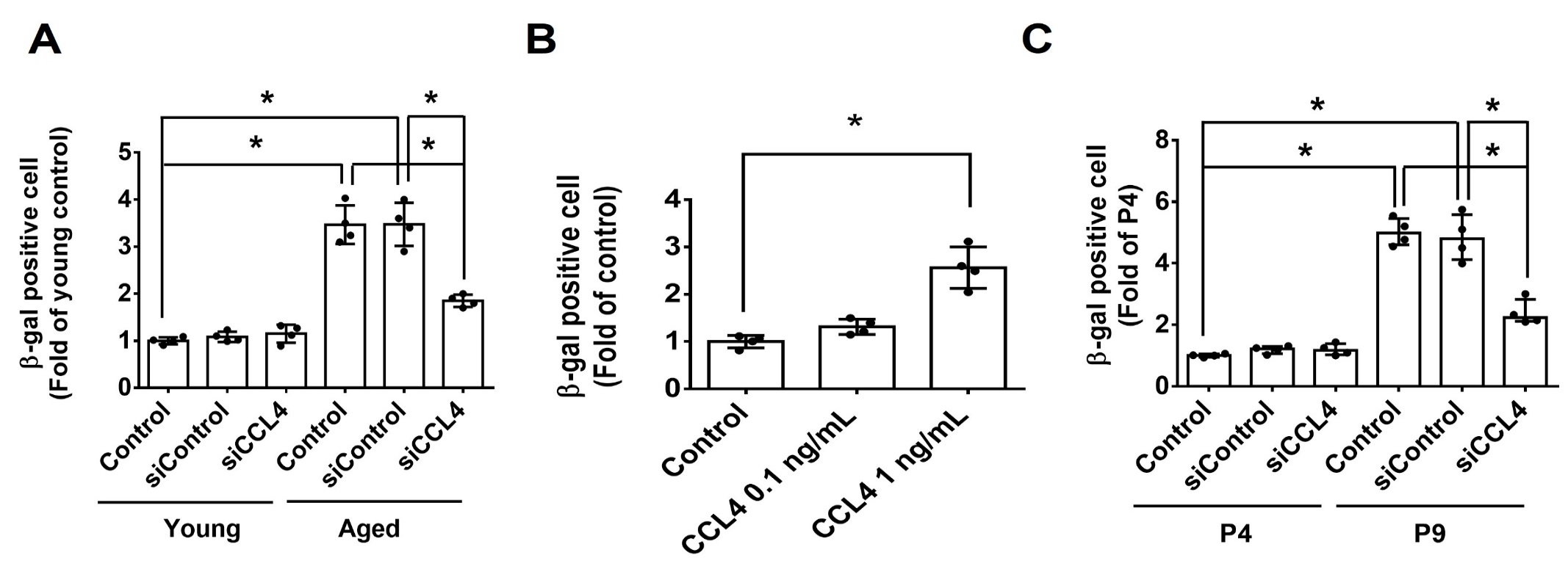


**Figure S1. The quantitative results of senescence. (A)** Inhibition of CCL4 reduced senescence of EPCs from aged subjects (n=4). **(B)** Cell senescence was increased in CCL4-treated HAECs (n=4). **(C)** Inhibition of CCL4 reduced senescence of aged HAECs (n=4). *P < 0.05, **P < 0.01.

**Figure S2**


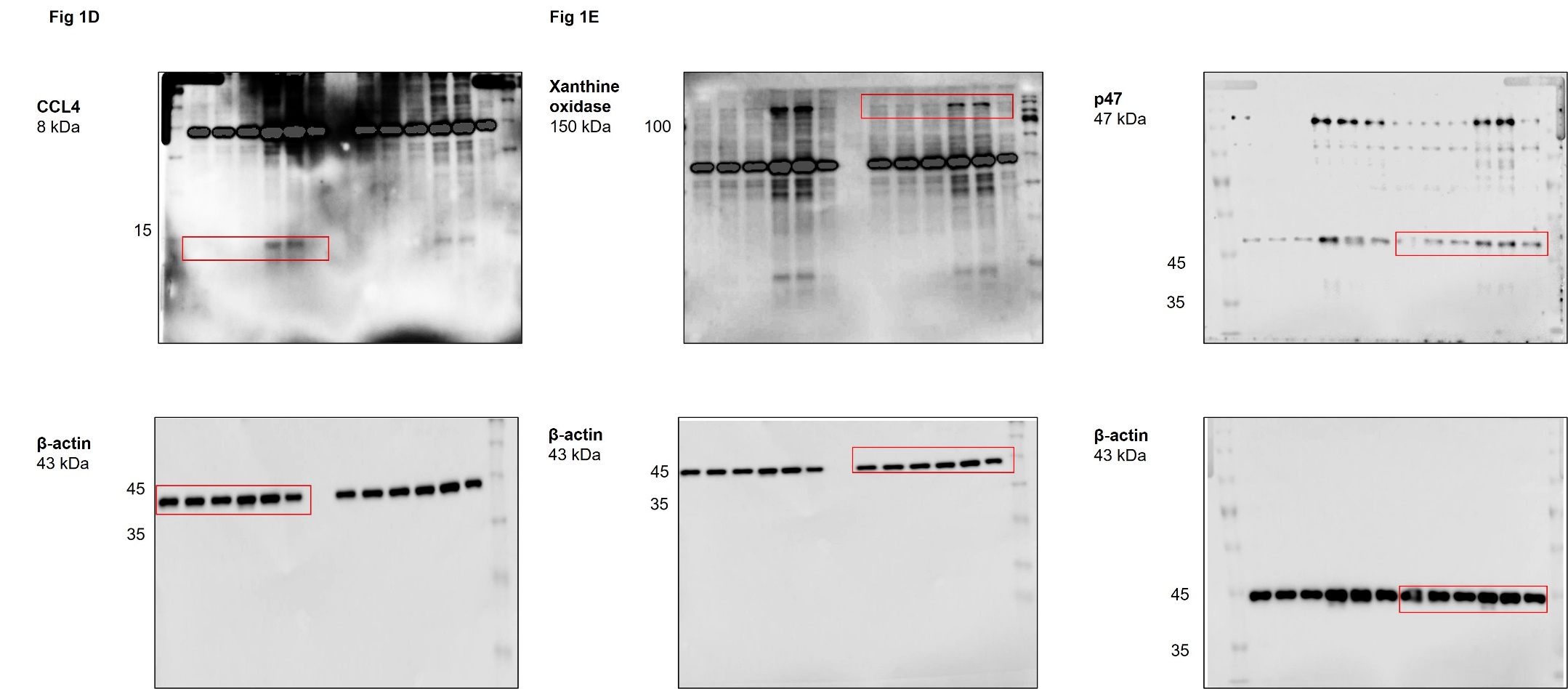

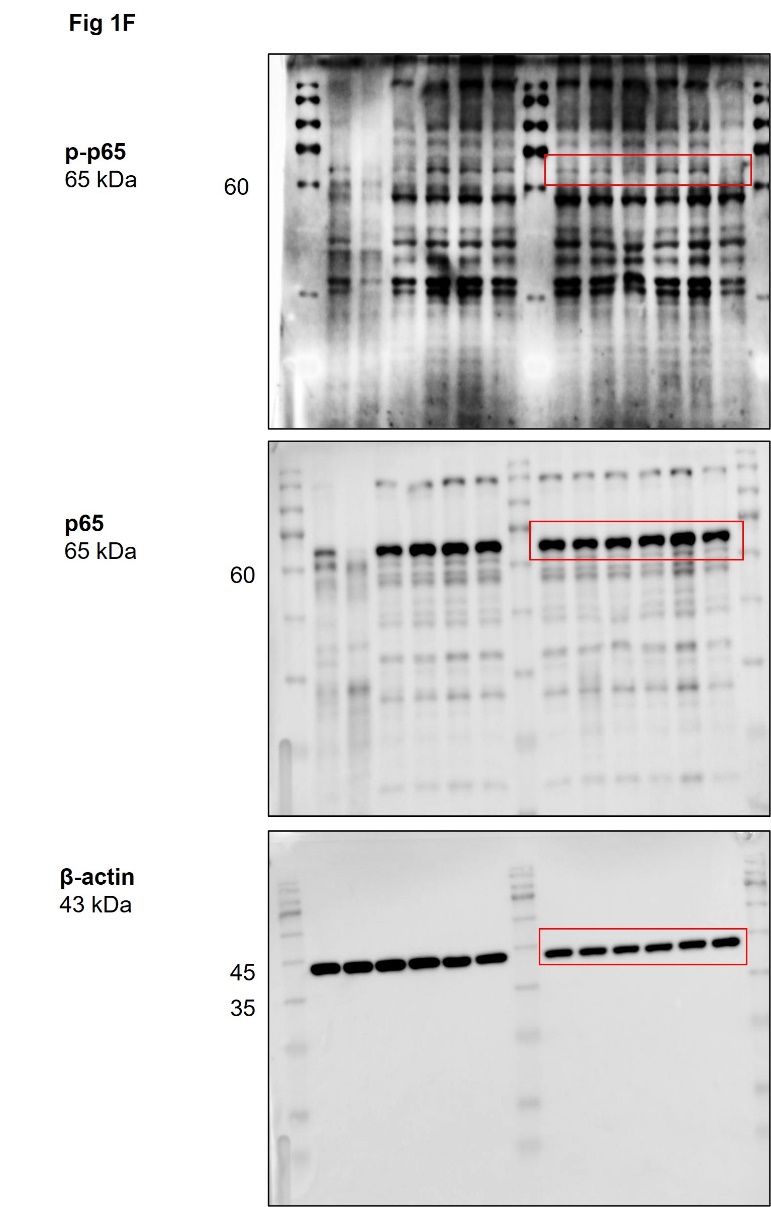

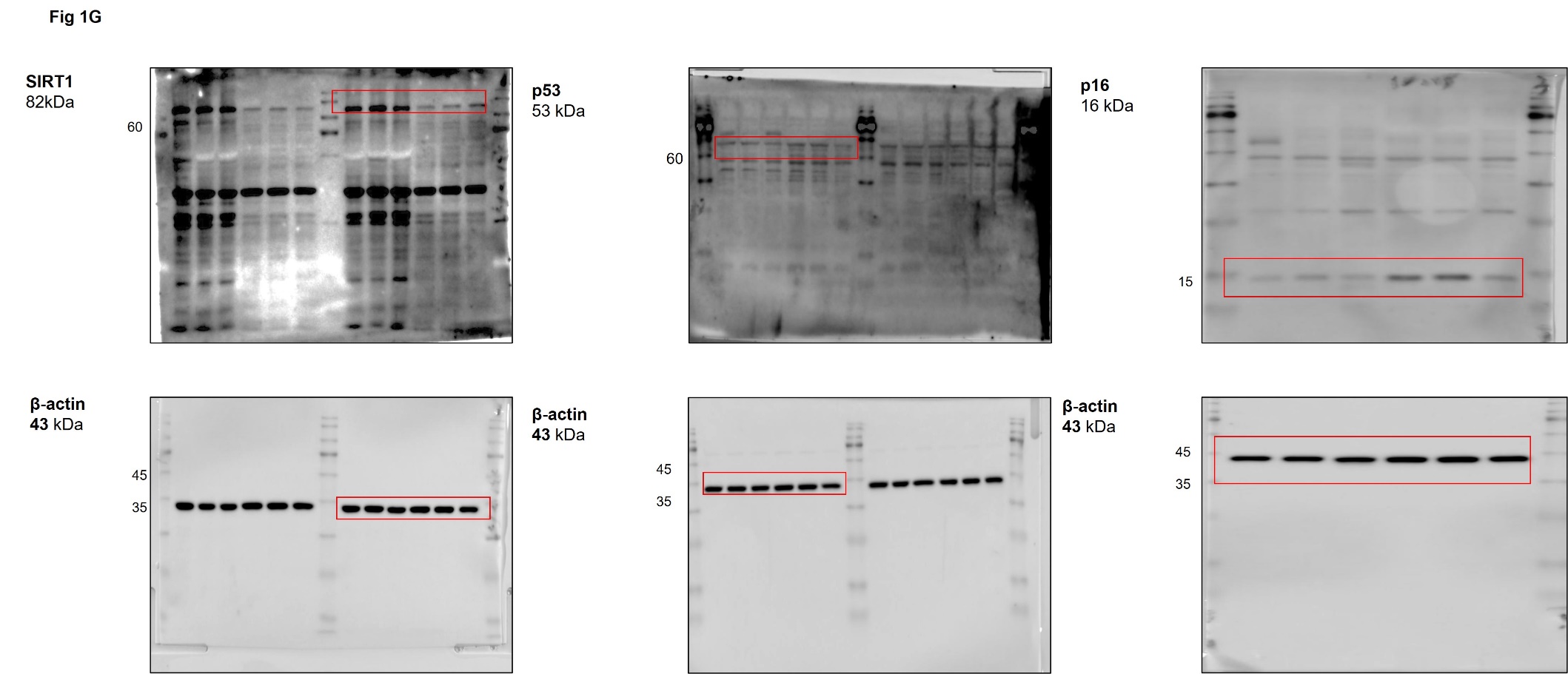


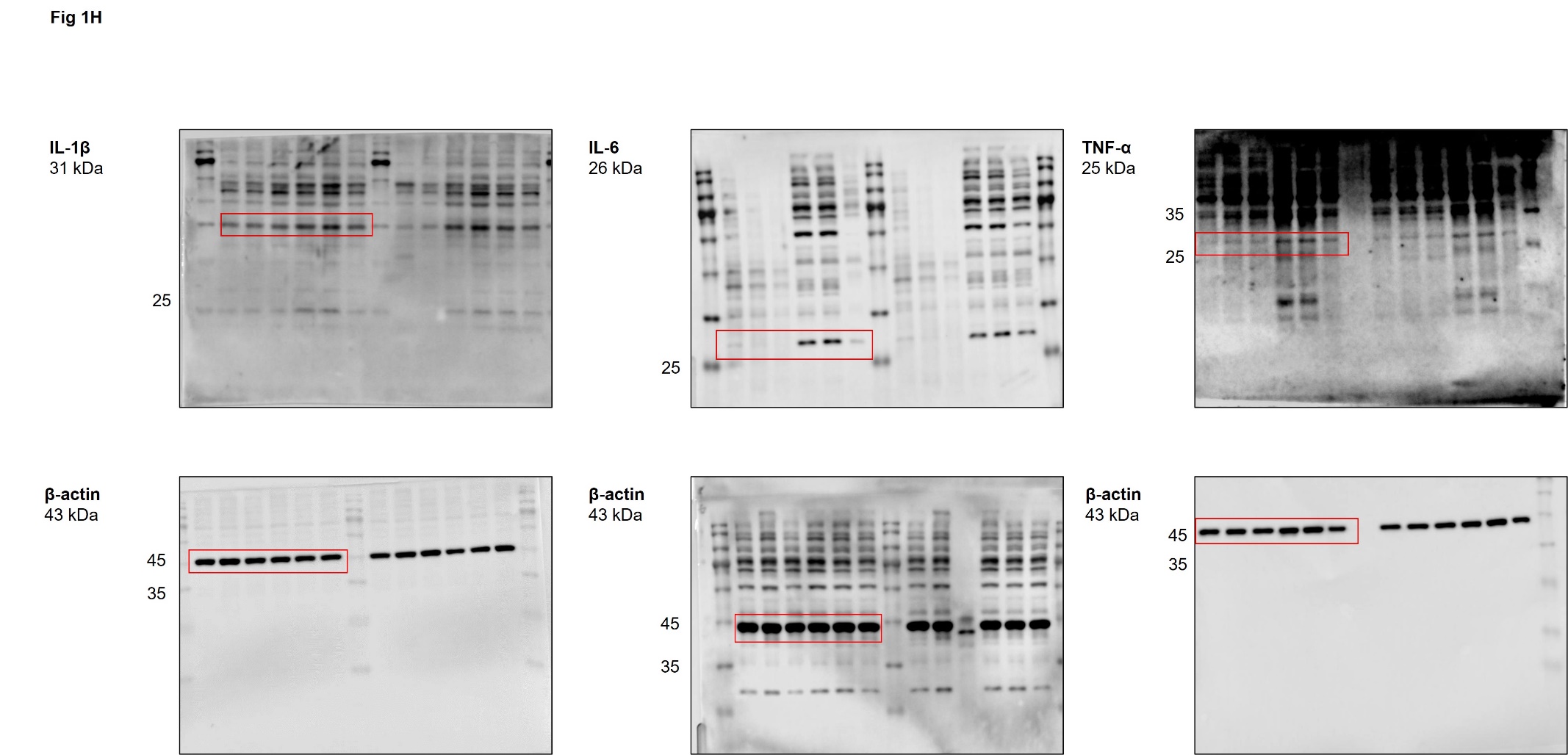

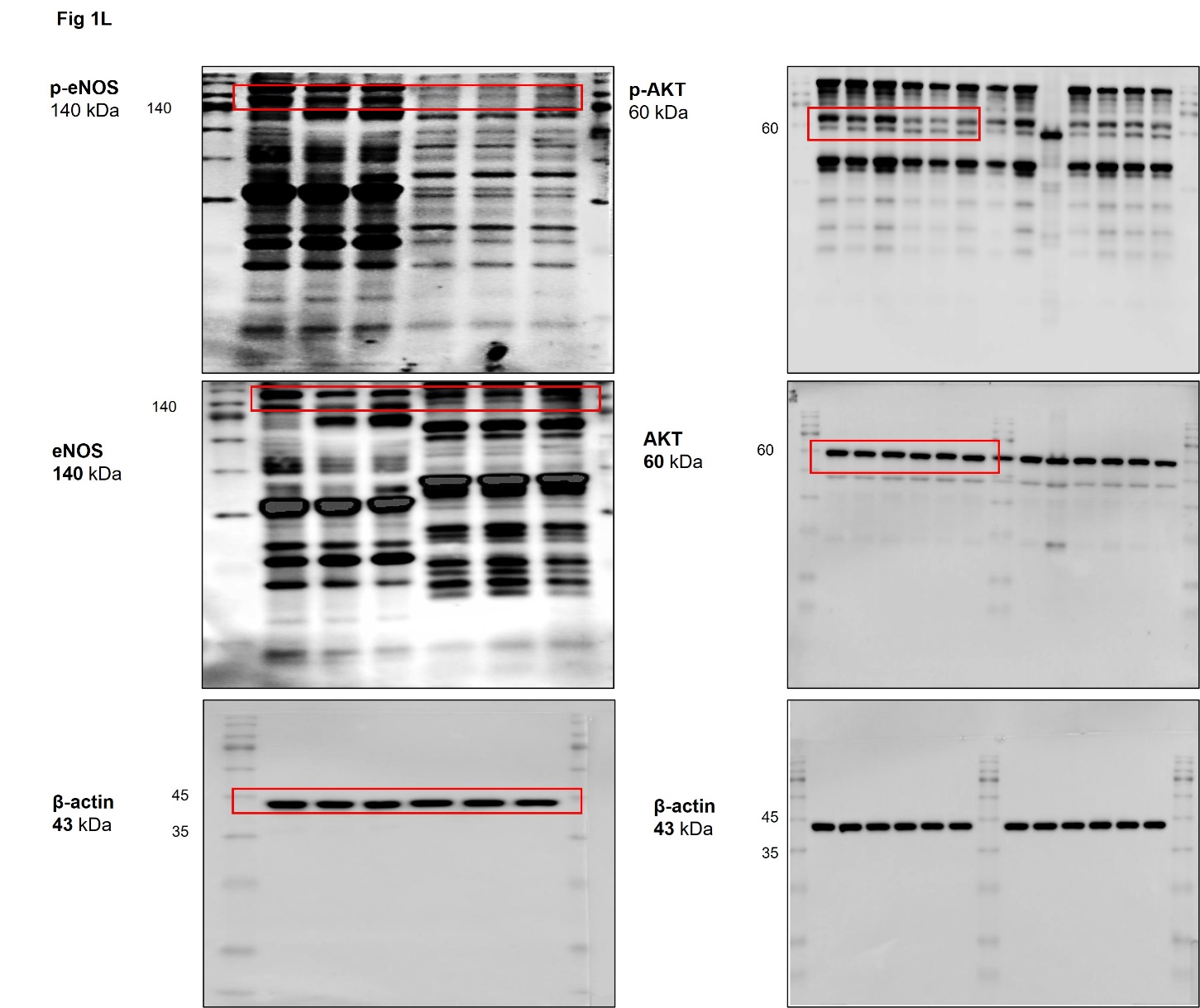

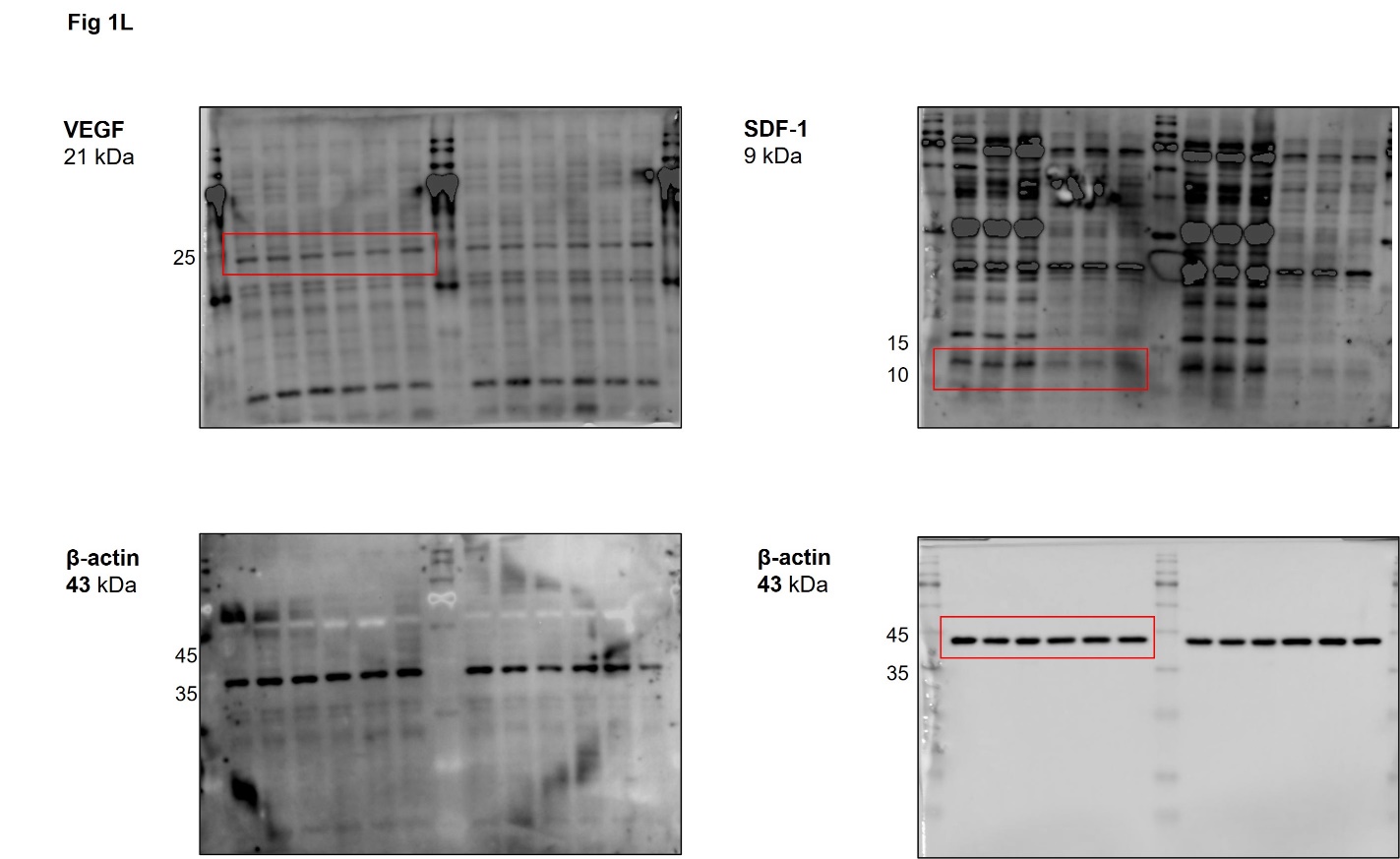

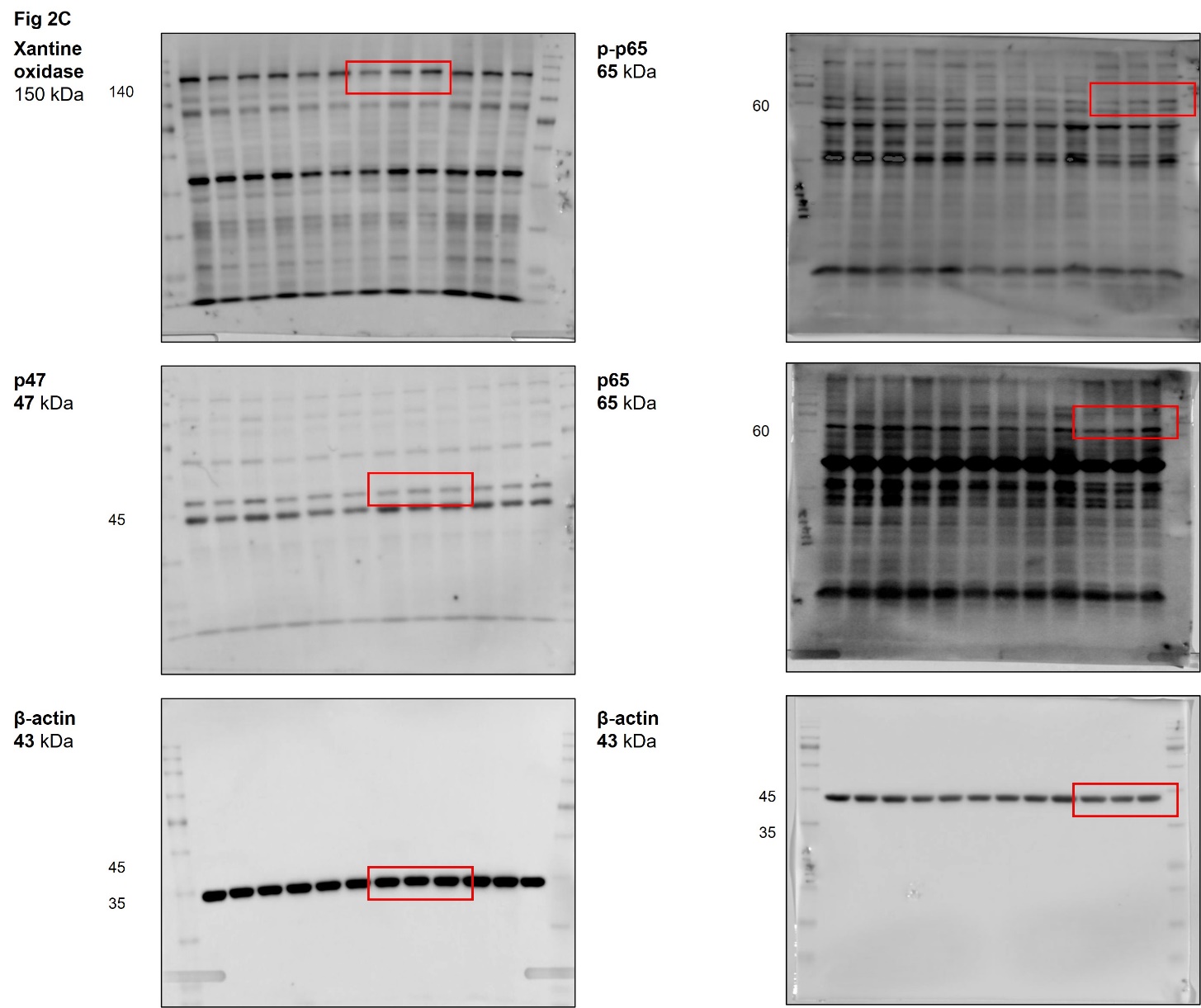

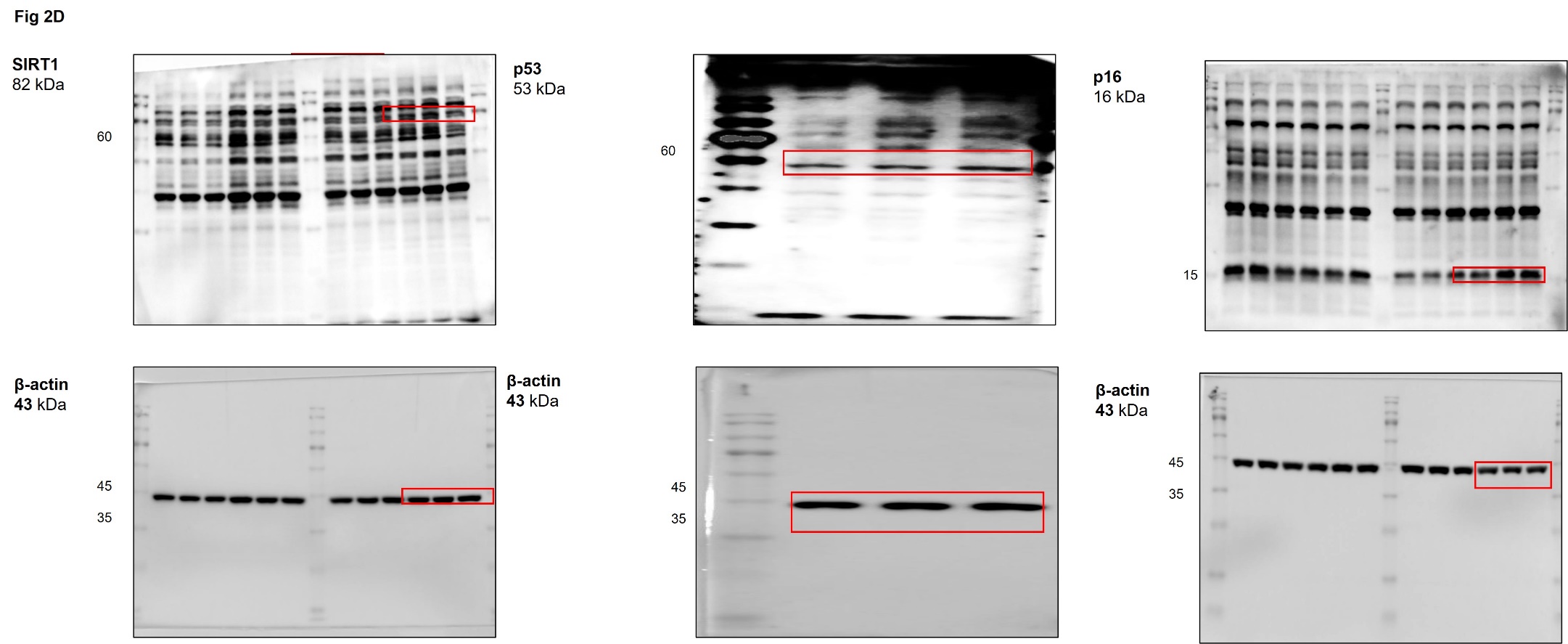

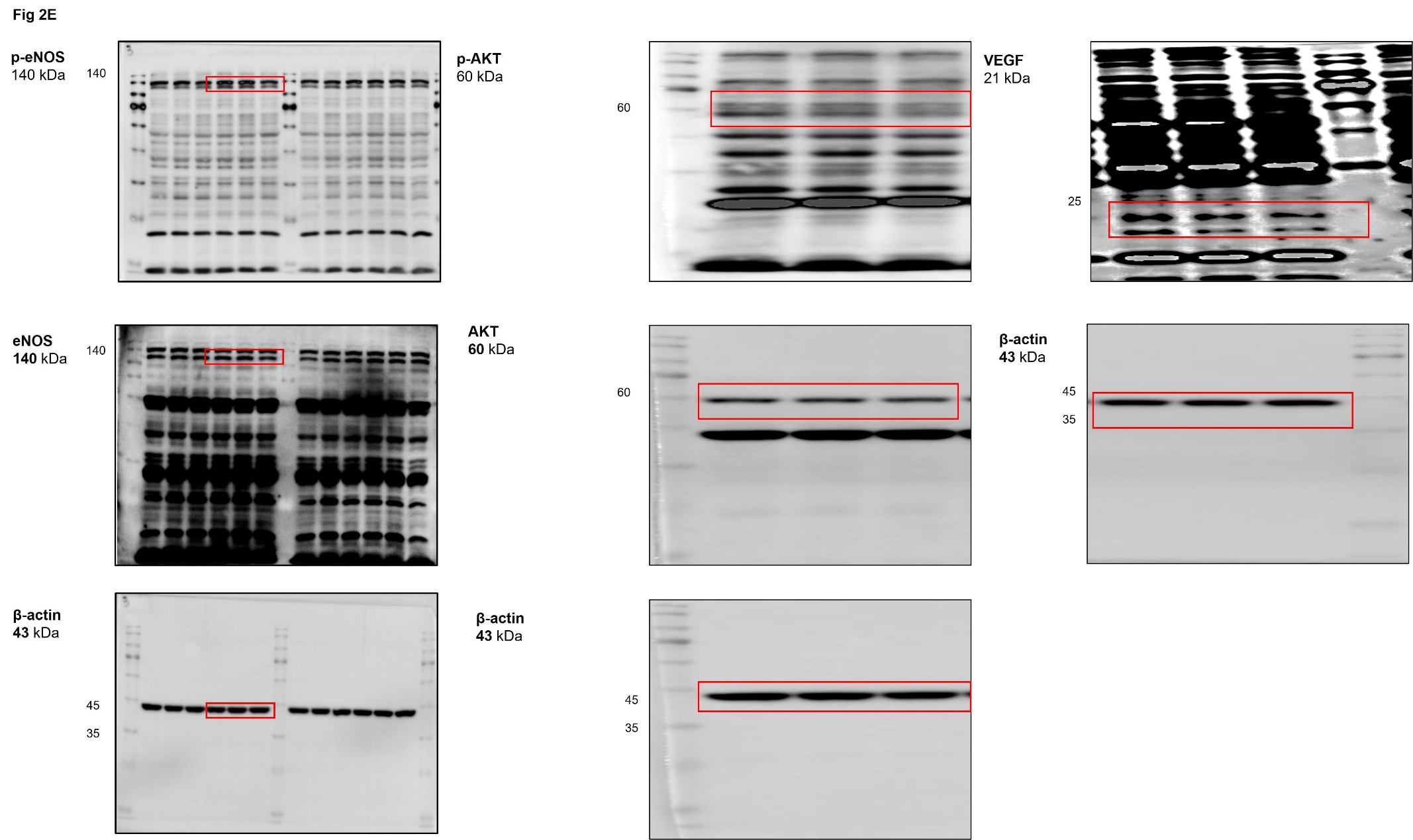

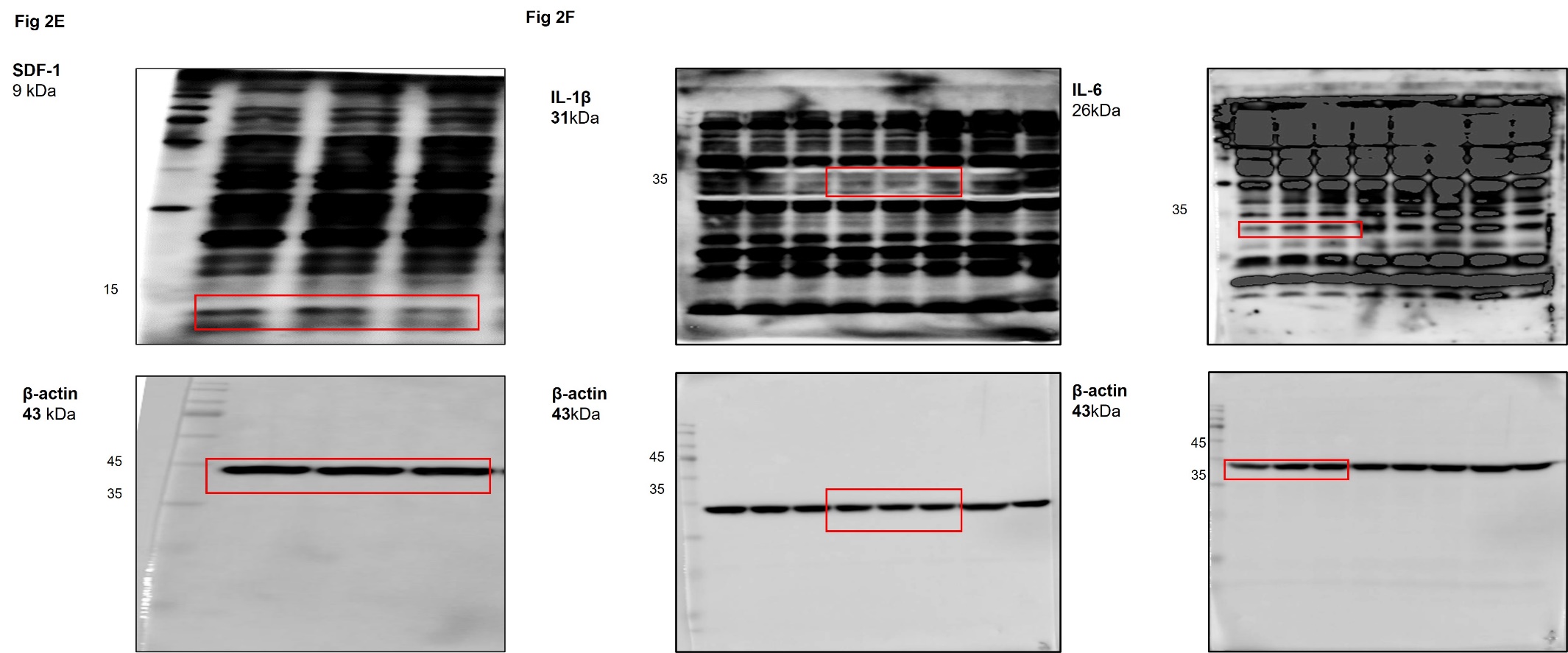

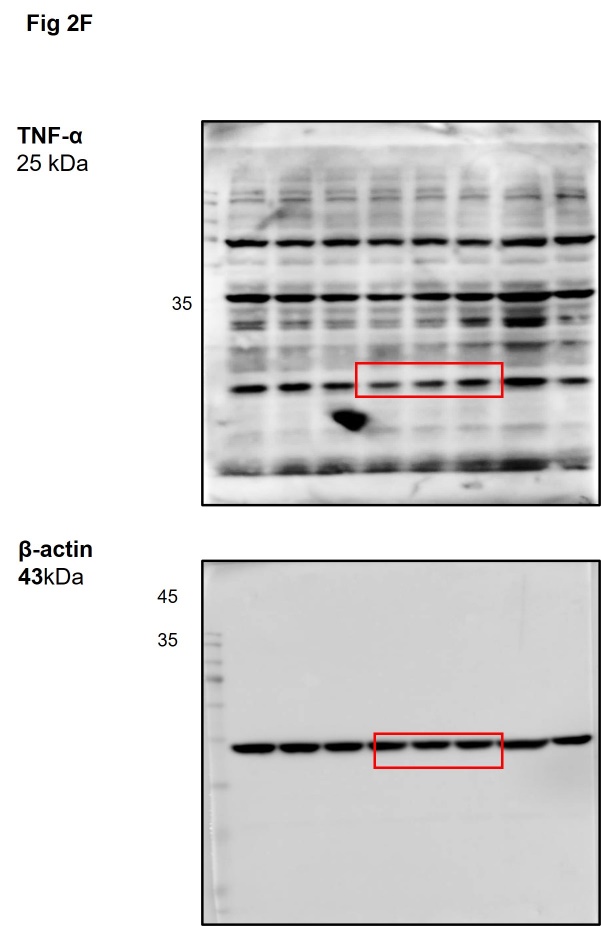

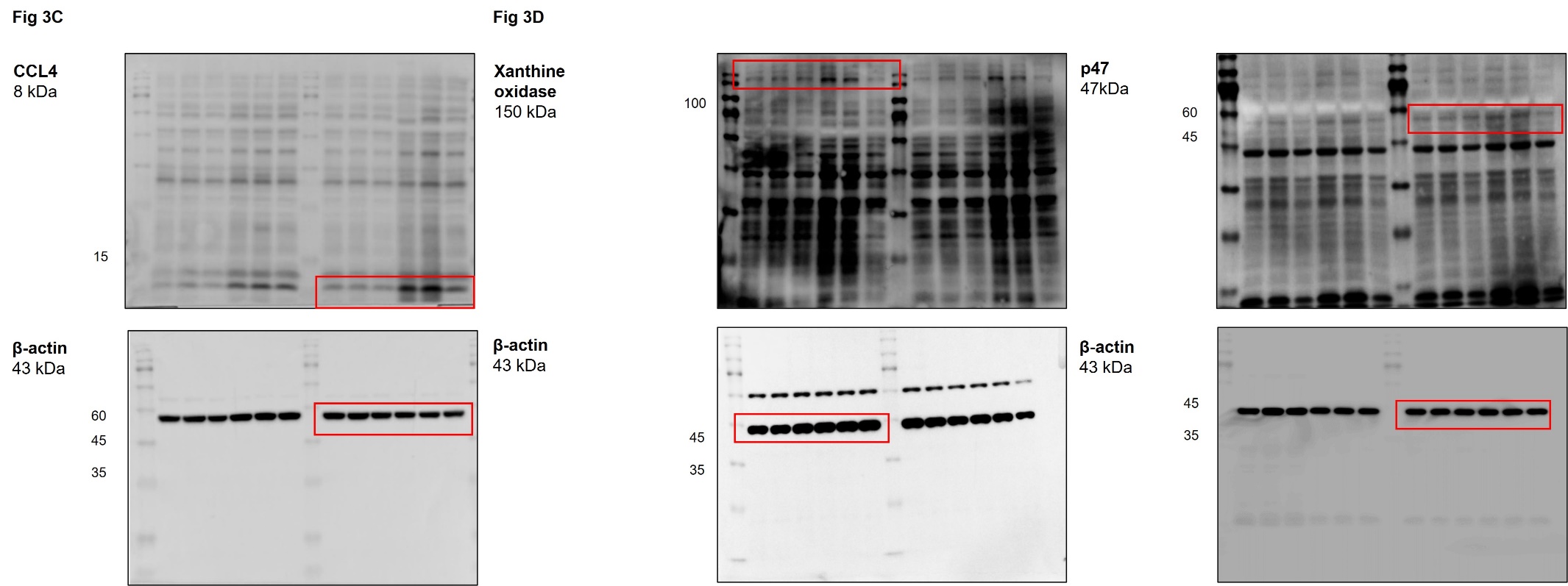

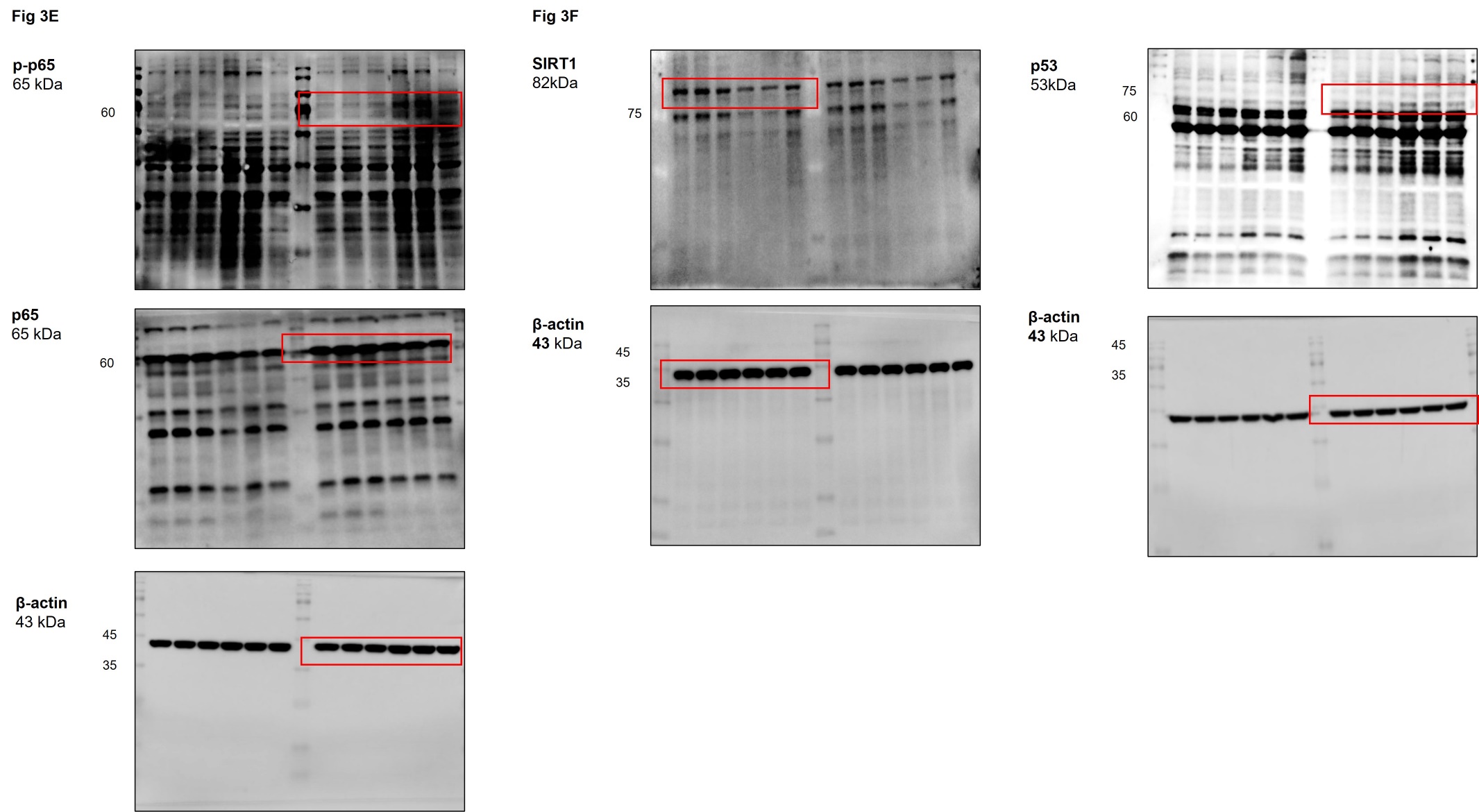

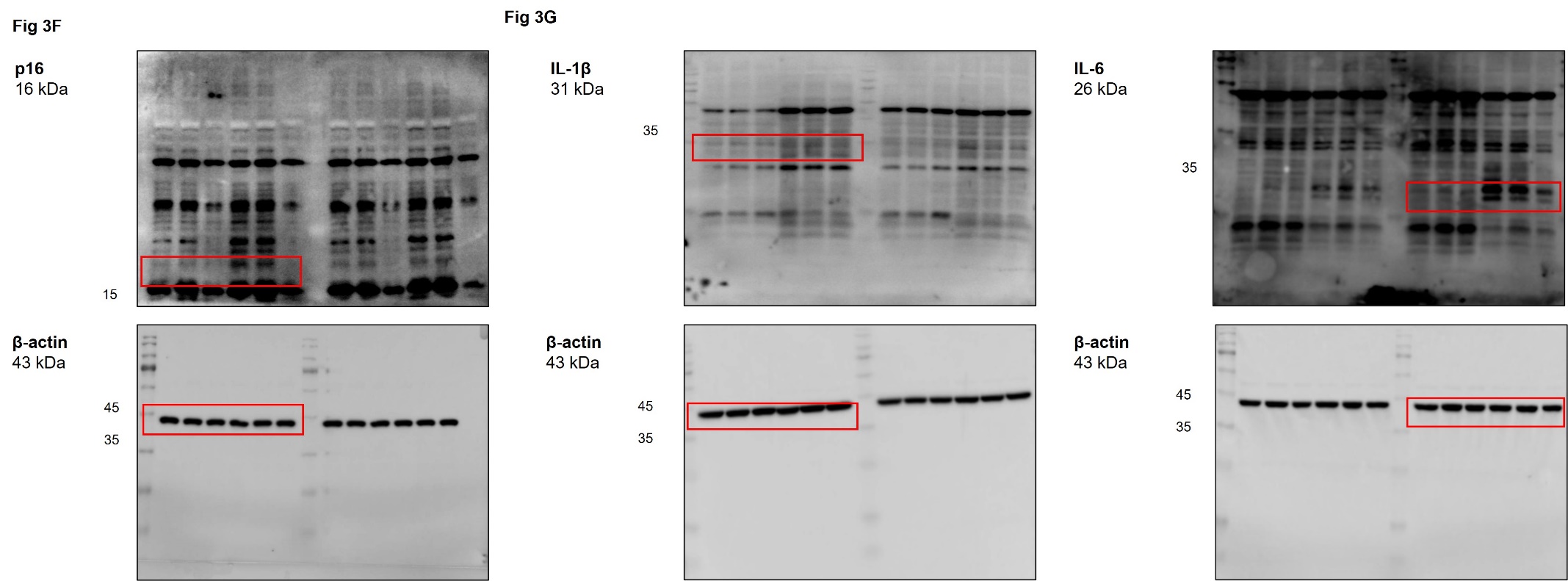

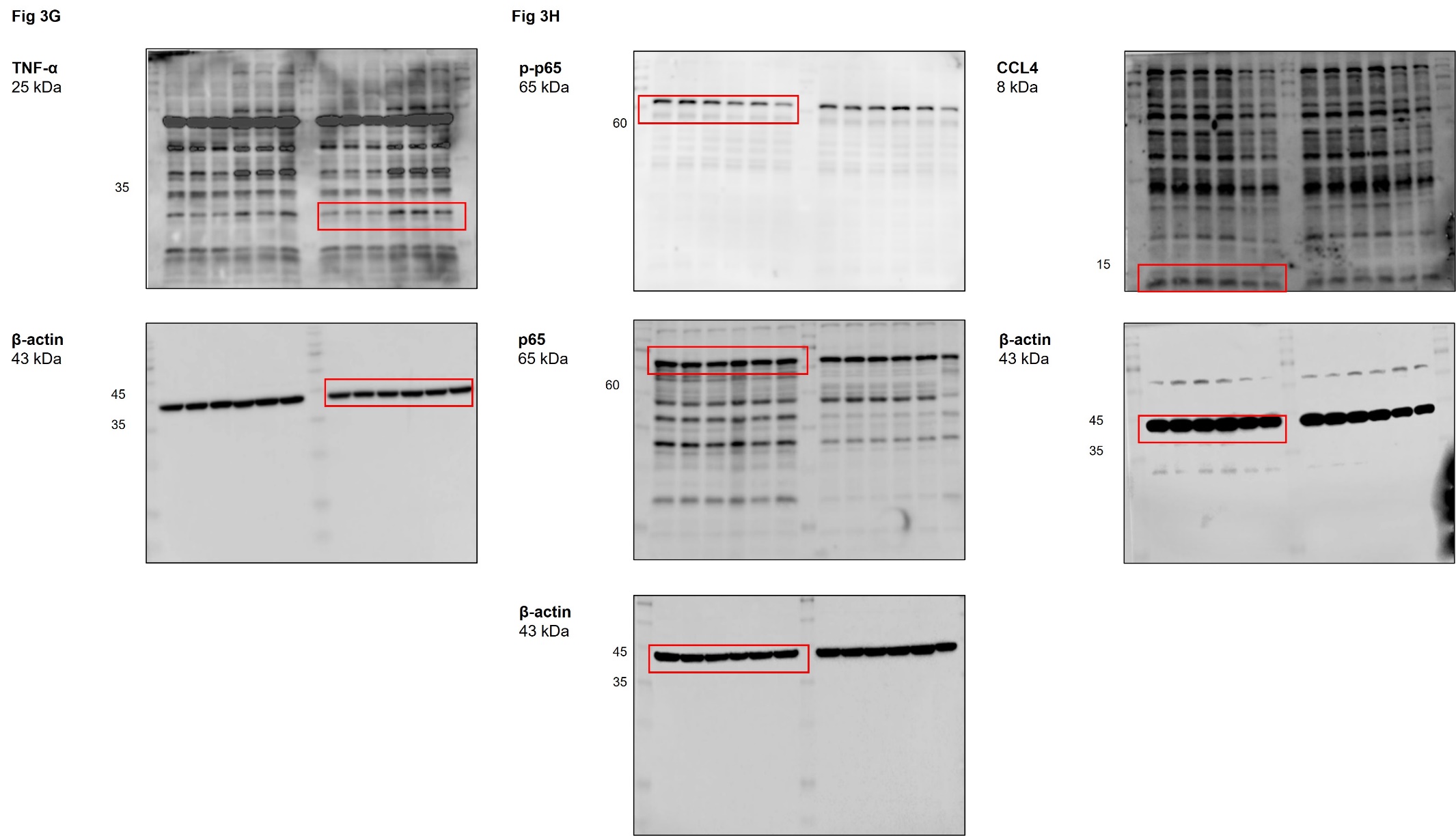

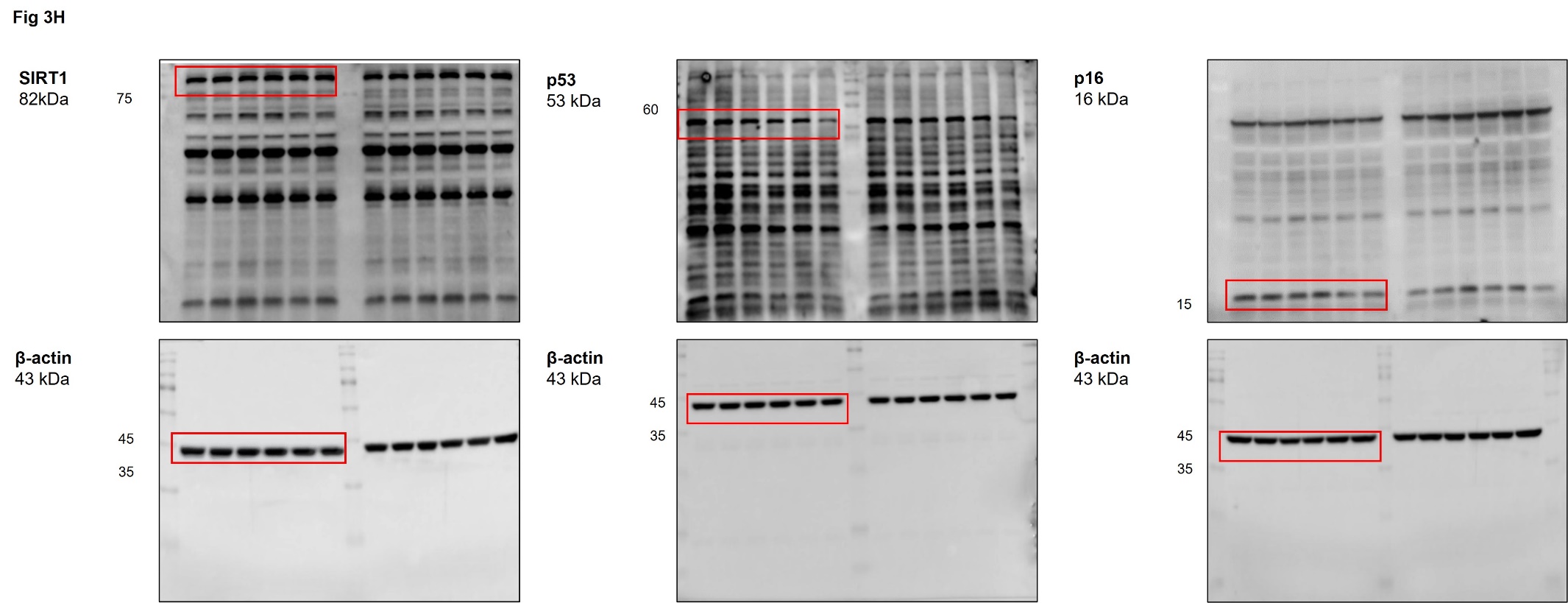

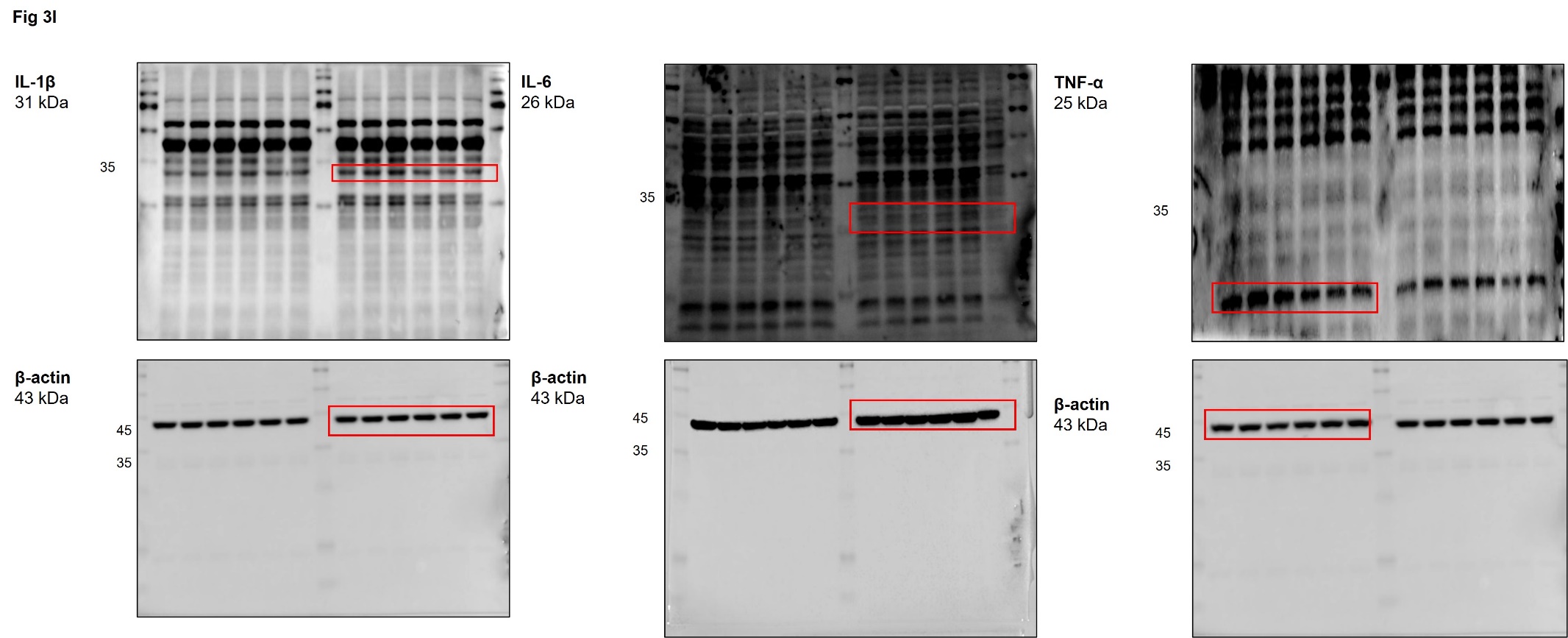

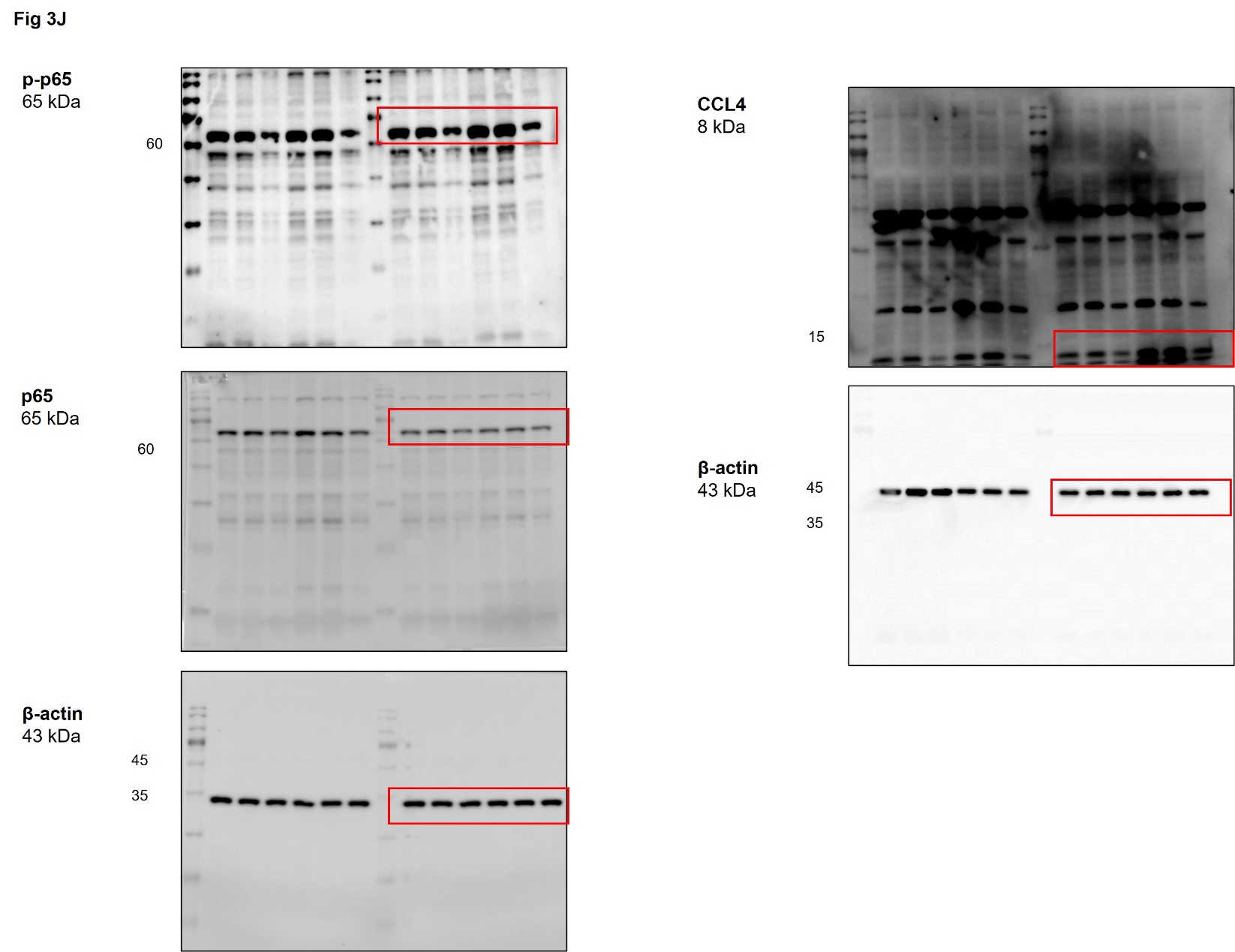

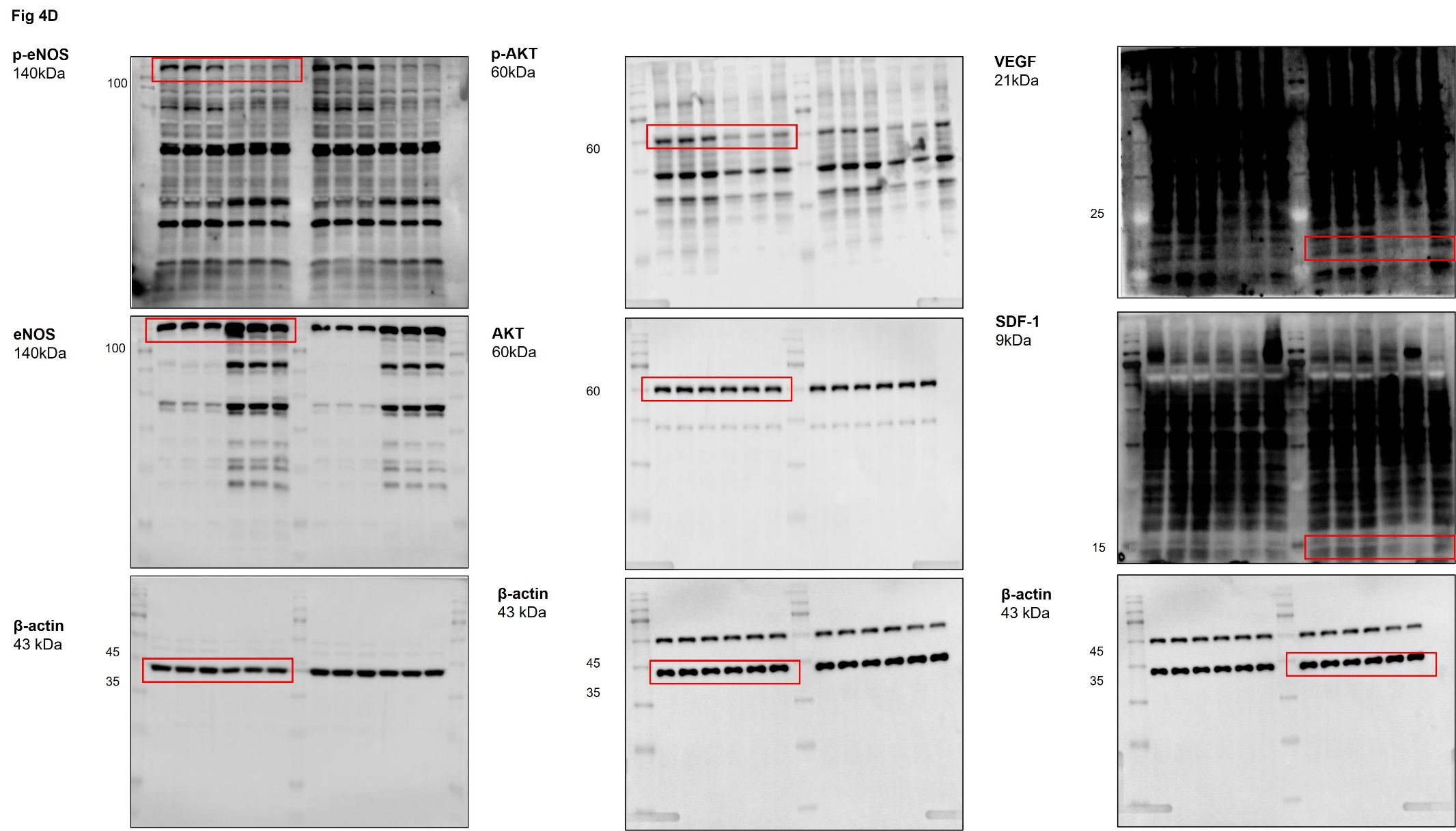

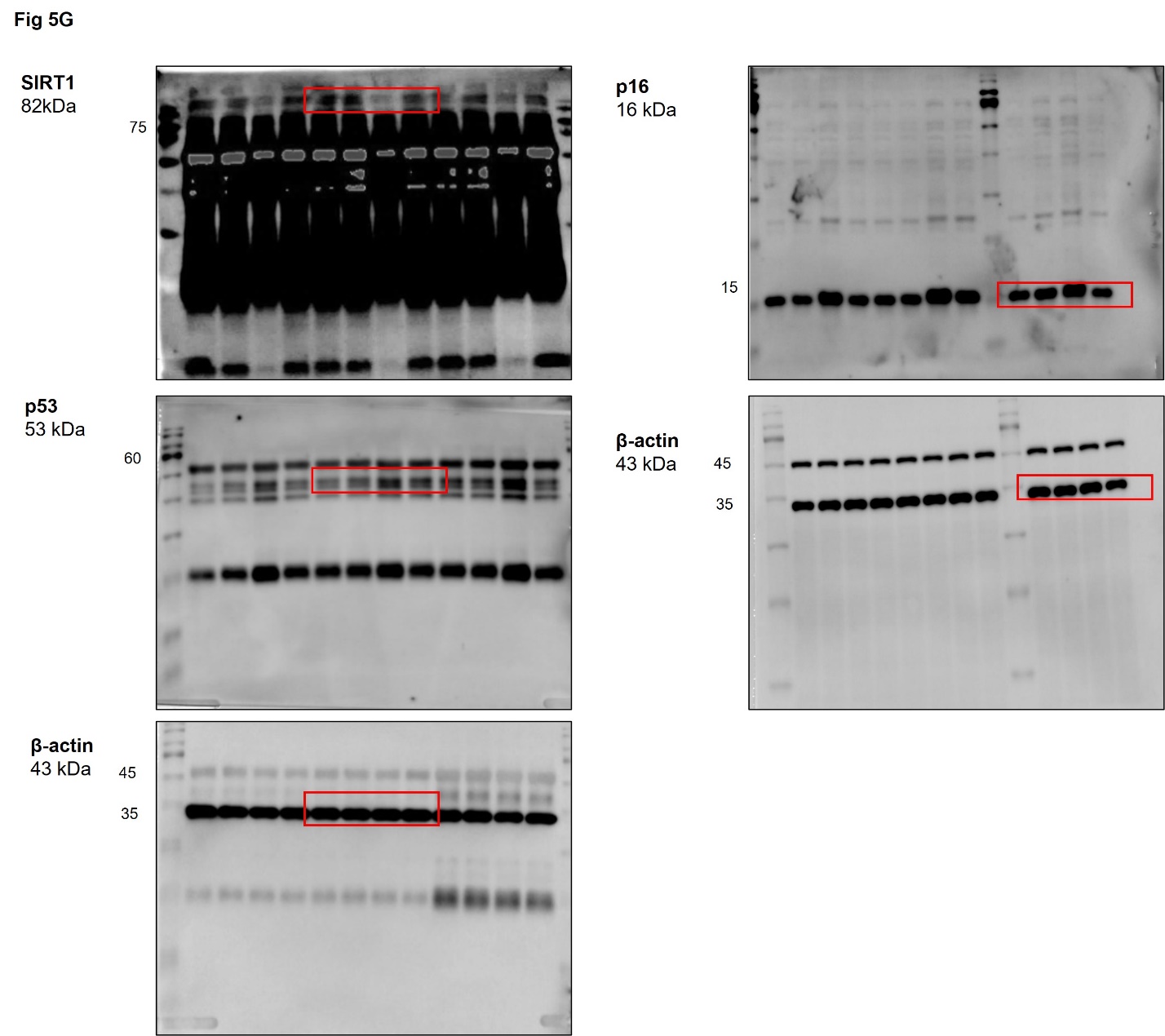

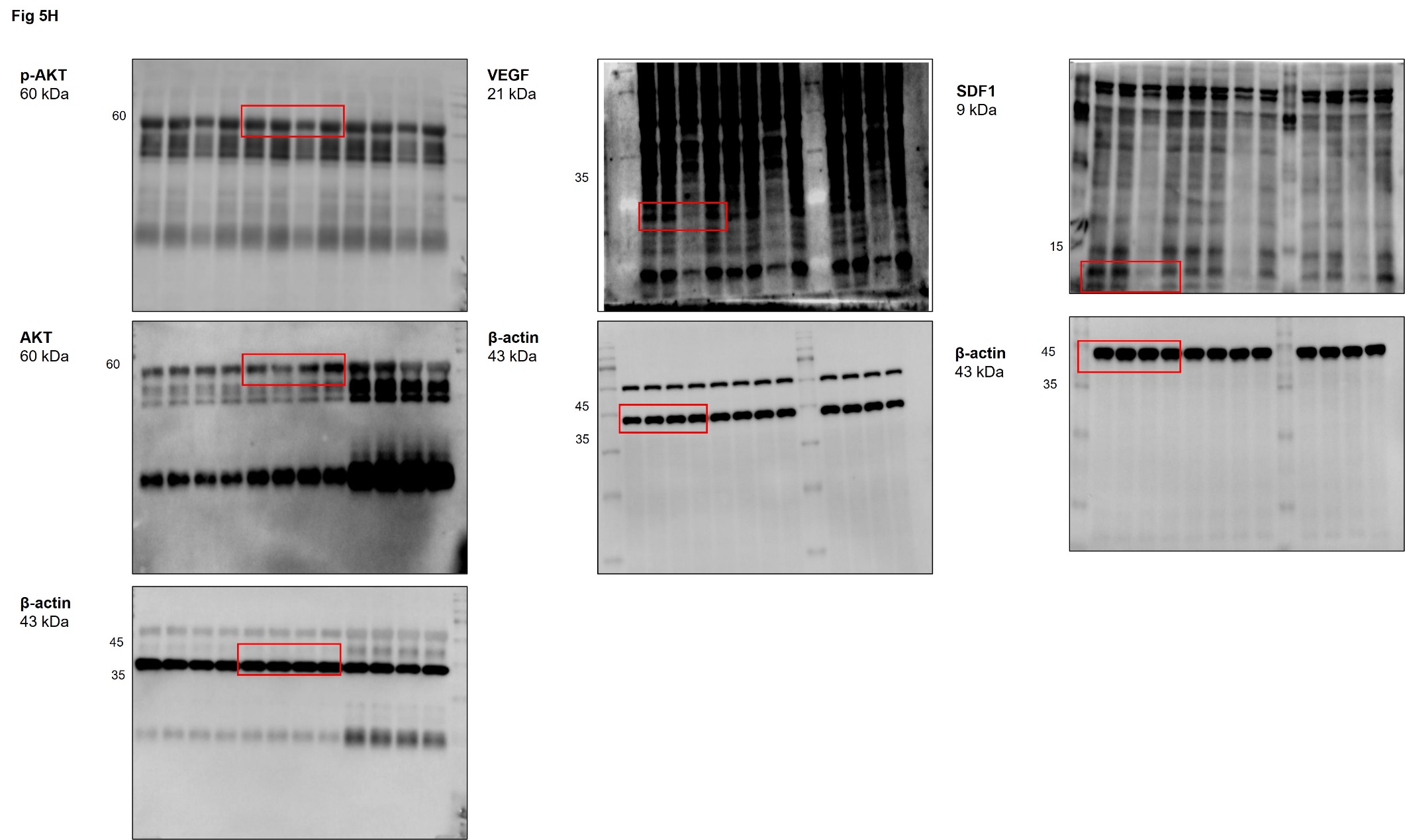

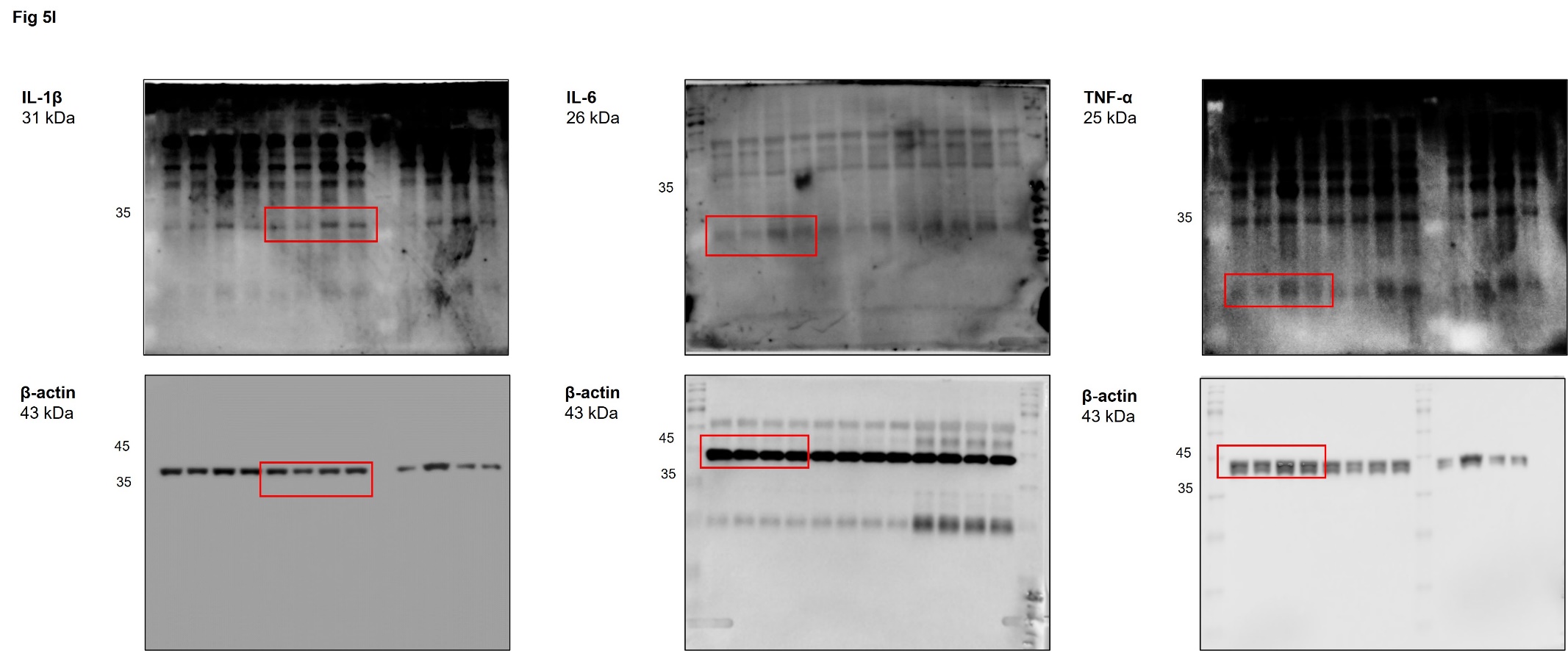

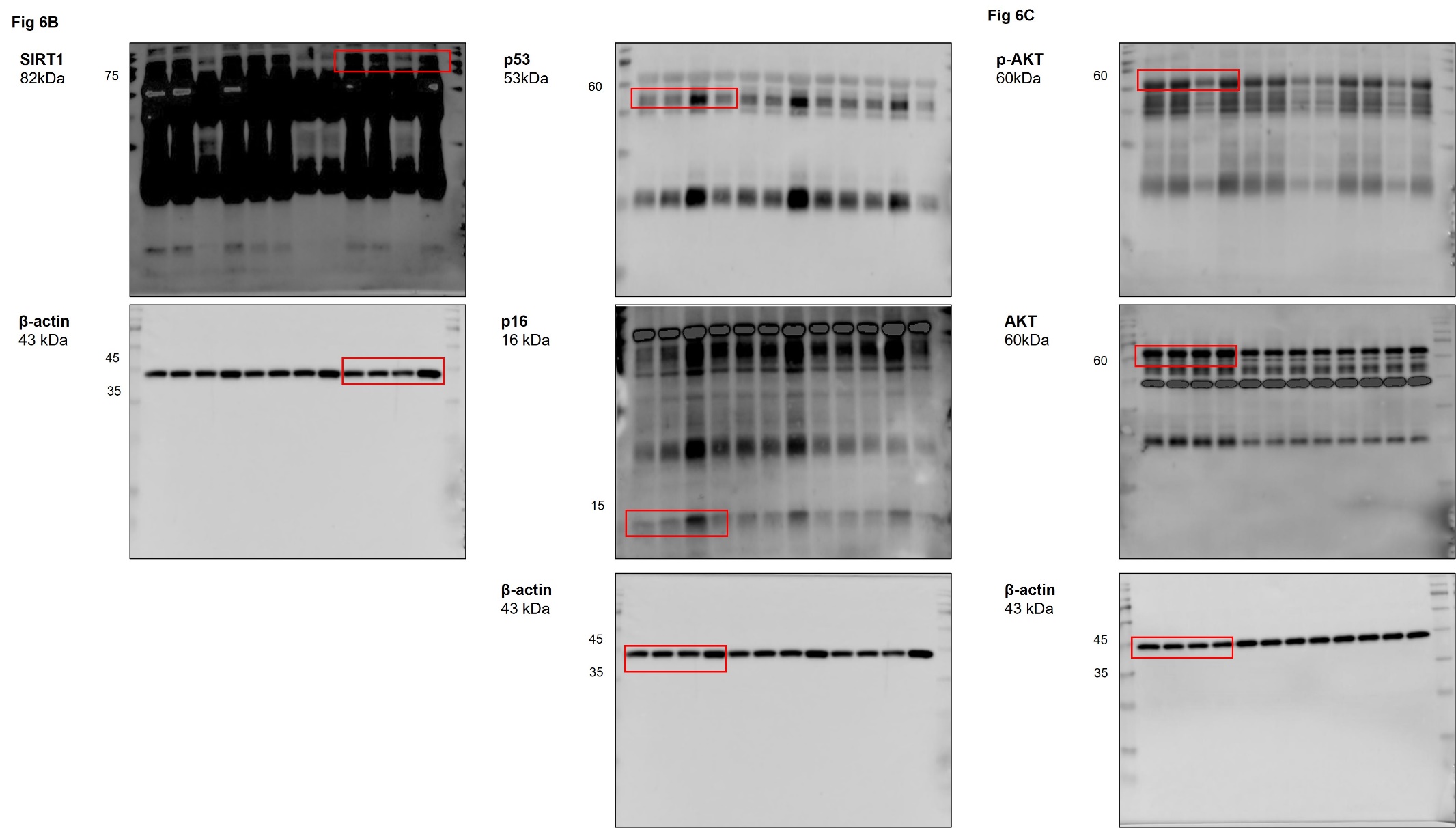

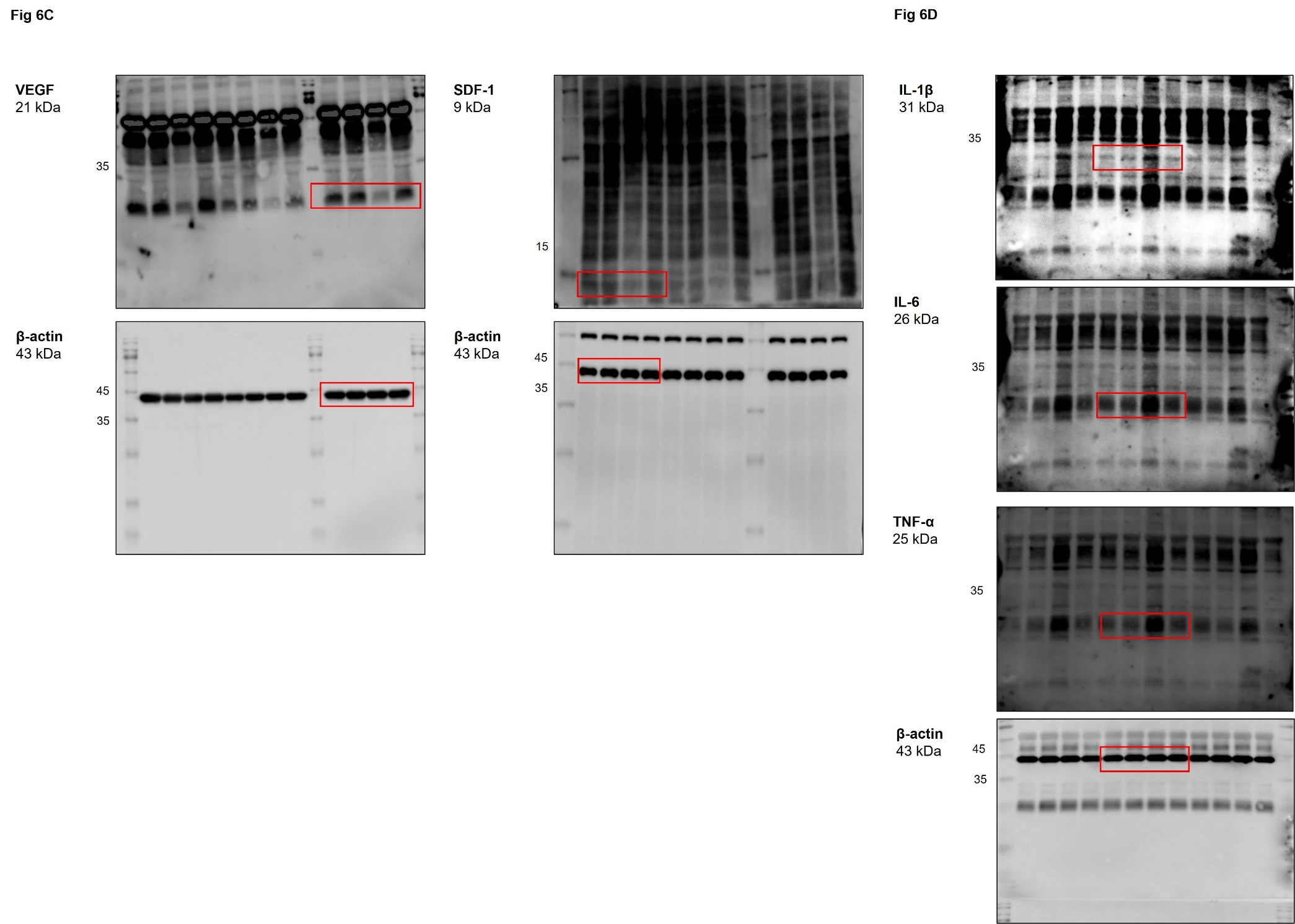

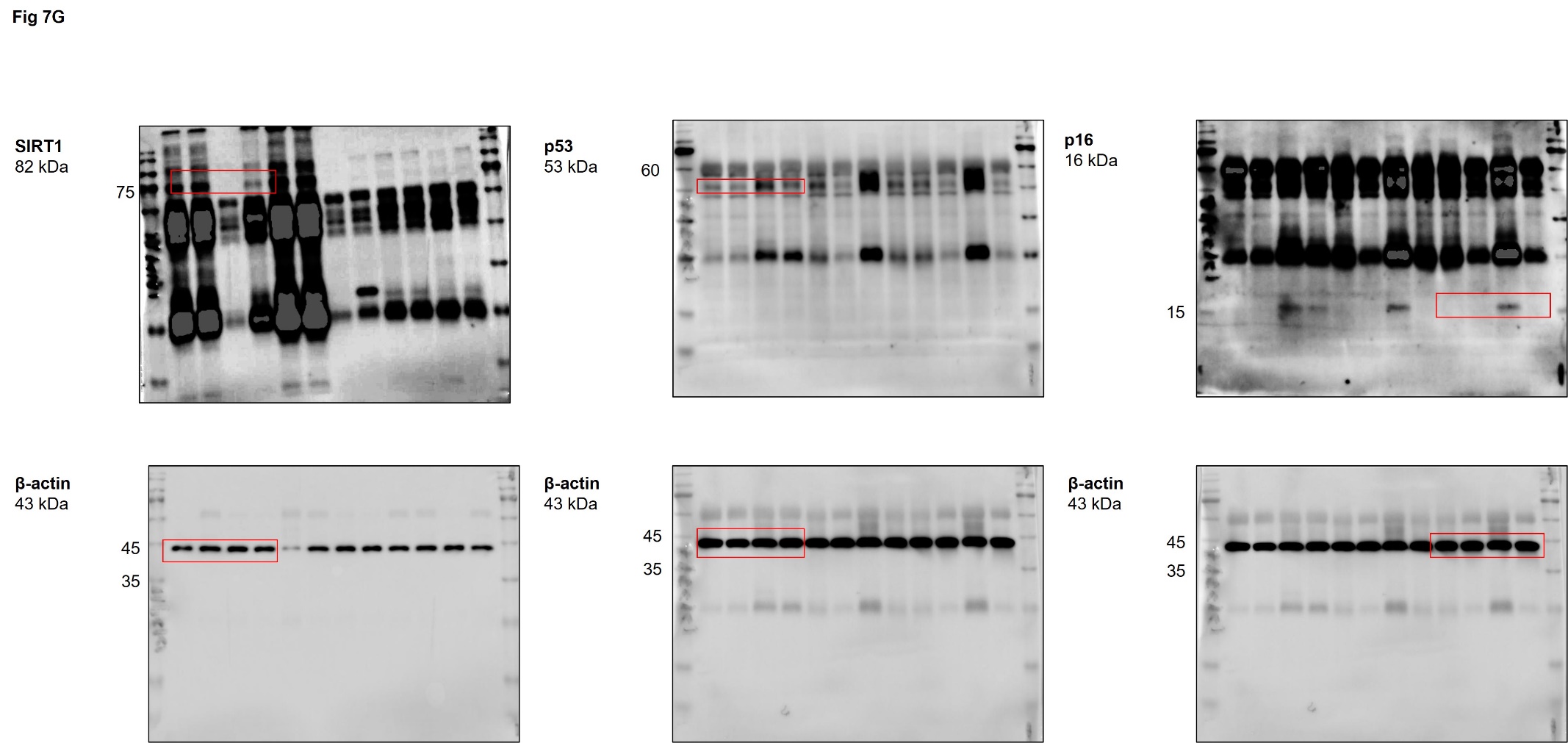

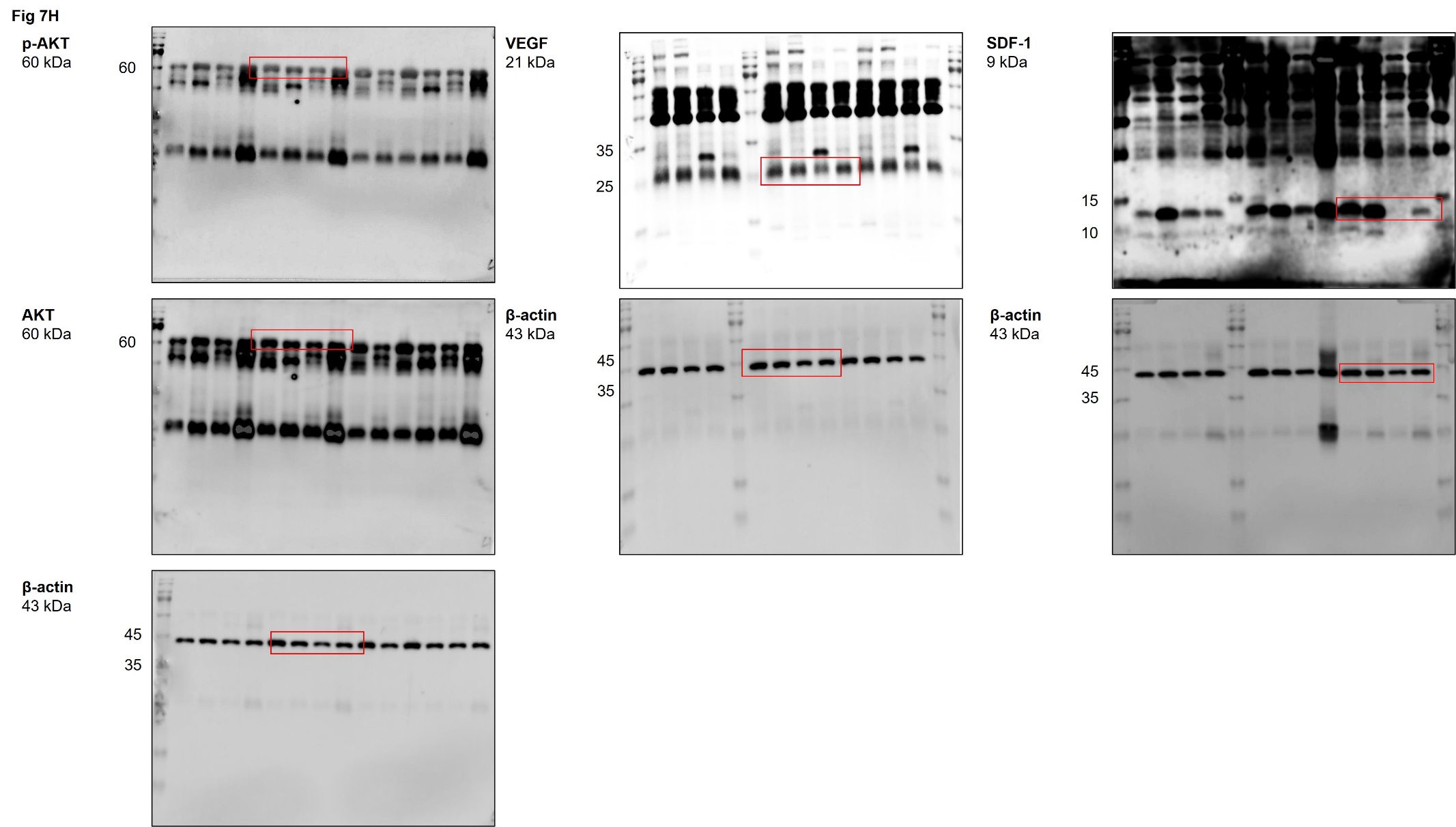

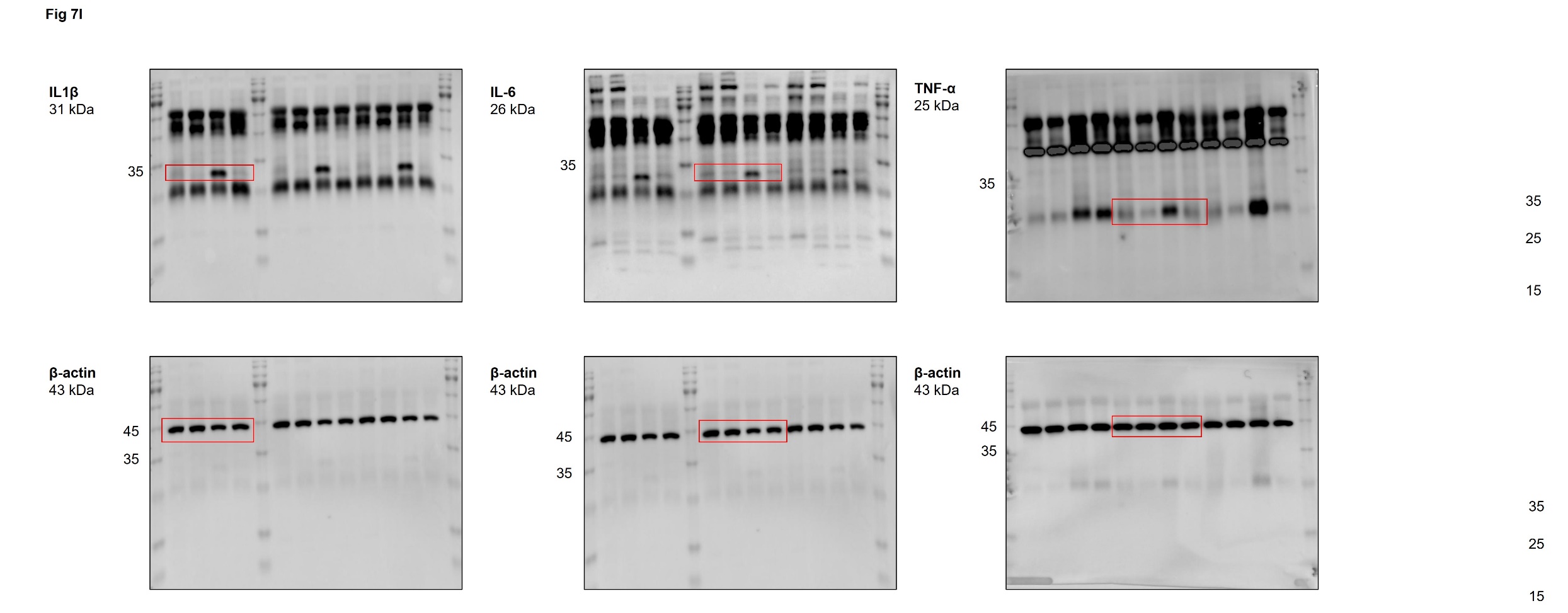

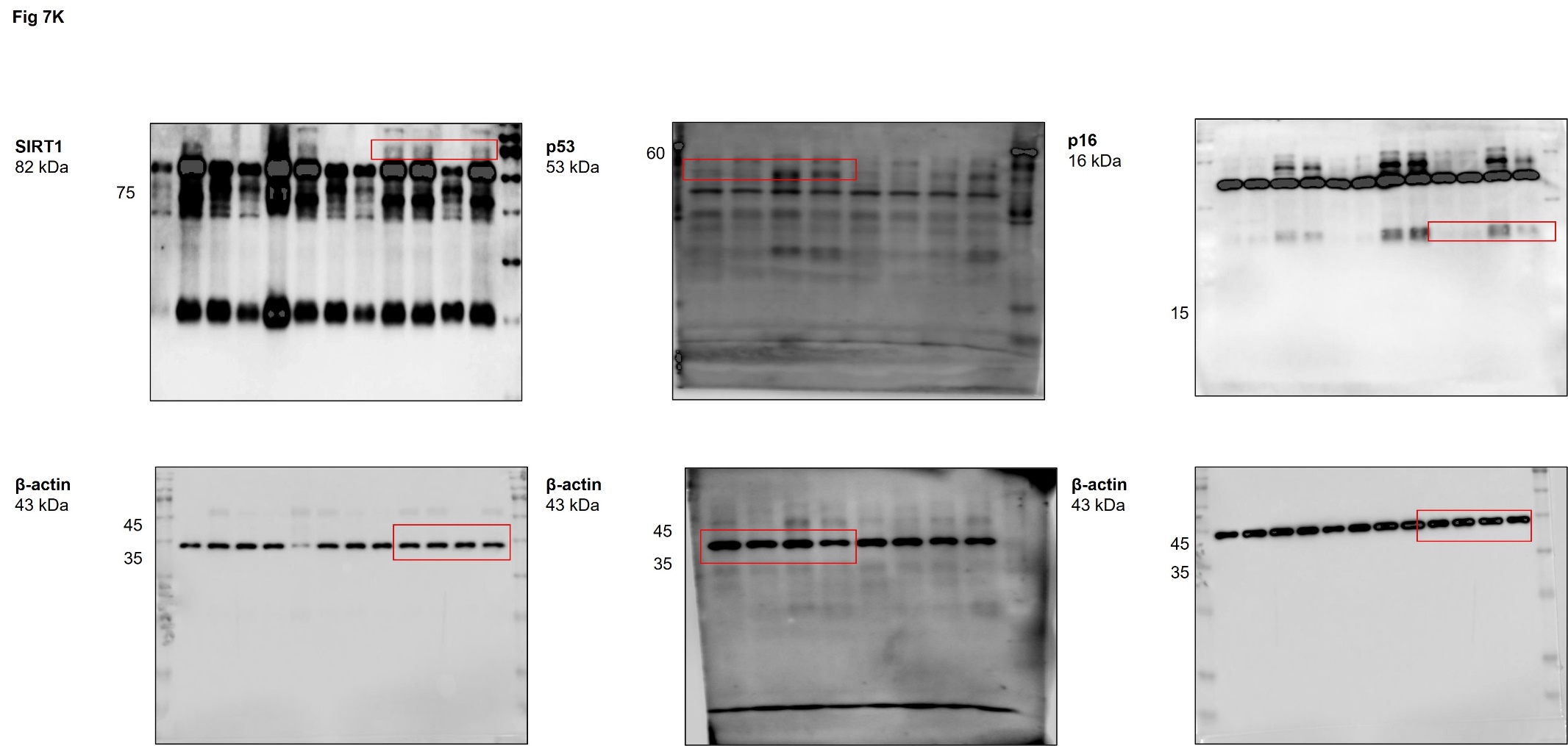

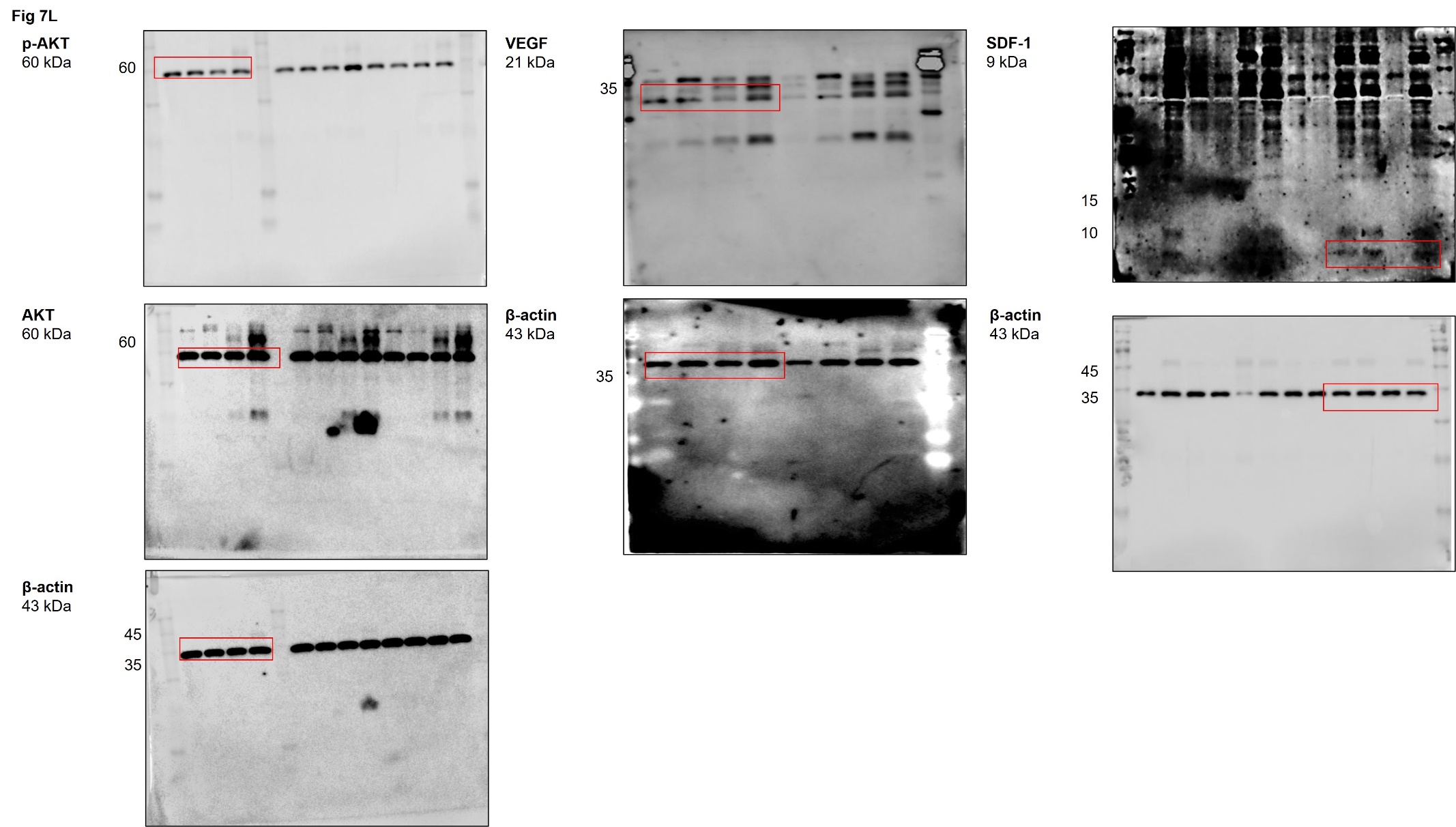

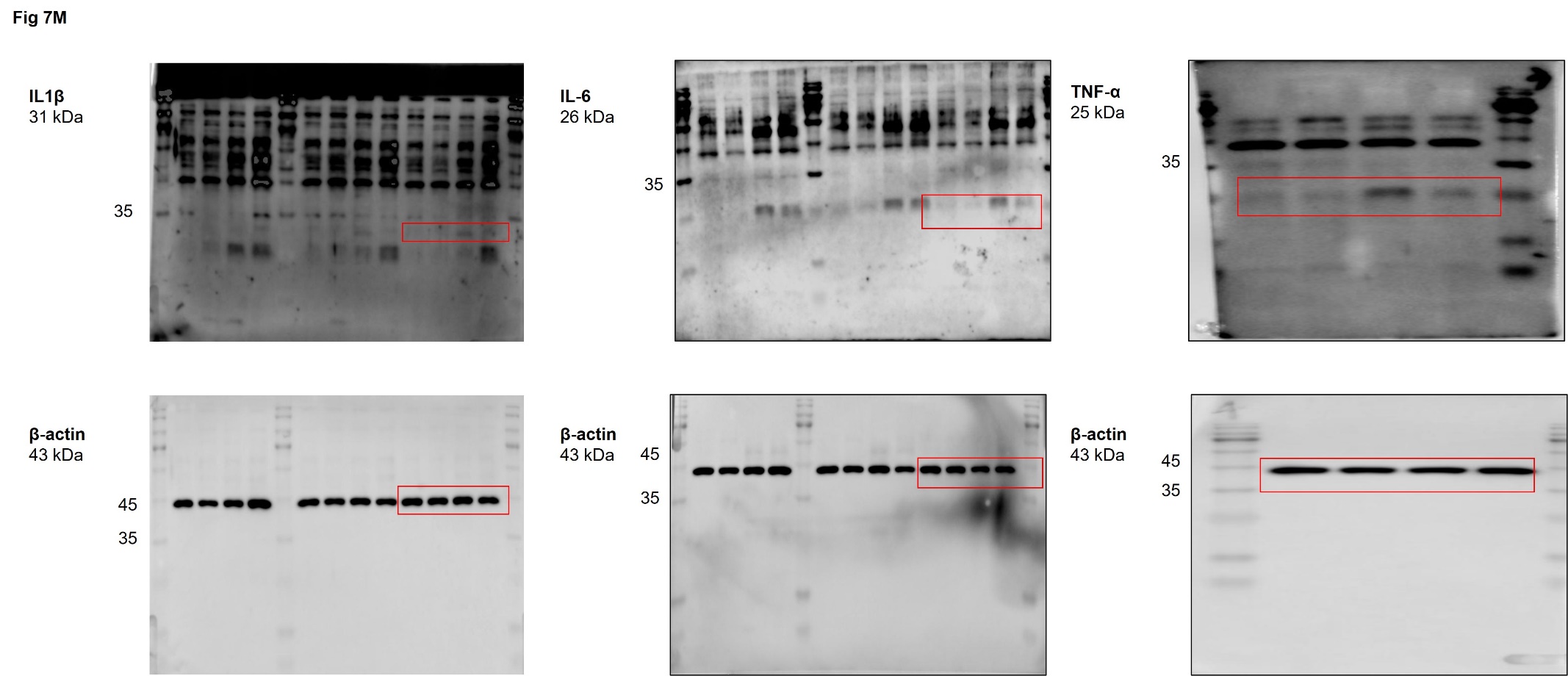


**Figure S2. Original Western blot images.**
